# Liquid Crystalline Fluorene‐2,1,3‐Benzothiadiazole Oligomers with Amplified Spontaneous Emission

**DOI:** 10.1002/marc.202500189

**Published:** 2025-04-14

**Authors:** Philipp J. Welscher, Ulrich Ziener, Alexander J. C. Kuehne

**Affiliations:** ^1^ Institute of Macromolecular and Organic Chemistry Ulm University Albert‐Einstein‐Allee 11 89081 Ulm Germany

**Keywords:** benzothiadiazoles, liquid crystals, oligofluorenes, organic lasers, photophysics

## Abstract

Conjugated fluorene‐based molecules are a powerful class of materials for optoelectronic applications, known for their outstanding photoluminescence quantum yields and easily tunable optical properties. While conjugated polymers like poly(fluorene‐*co*‐benzothiadiazole) have been extensively studied, their performance is often hindered by product inhomogeneity and morphological constraints. By contrast, well‐defined oligofluorenes offer precise molecular structures and better morphological control, making them an attractive alternative to conjugated polymers. Among them, benzothiadiazole (BT)‐centered oligofluorenes exhibit strong yellow emission with remarkably high photoluminescence quantum yields, yet their morphological properties remain largely unexplored. In this study, a straightforward synthesis of BT‐cored pentafluorenes is reported, where the alkyl side chains are systematically varied to investigate their impact on the morphology. These pentamers demonstrate amplified spontaneous emission (ASE) with thresholds as low as 1.64 µJ cm⁻^2^. By fine‐tuning the alkyl chains, crystalline, amorphous, and liquid crystalline morphologies are achieved, while maintaining consistent optical properties, paving the way for defined materials in advanced optoelectronic applications.

## Graphical abstract

Conjugated oligofluorenes with a benzothiadiazole (BT) core exhibit strong photoluminescence and promising optoelectronic properties. This study presents a streamlined synthesis of BT‐cored pentafluorenes with varied alkyl side chains to explore their morphological diversity. By achieving crystalline, amorphous, and liquid crystalline states while preserving optical performance, these materials offer a pathway to enhanced optoelectronic applications, including low‐threshold amplified spontaneous emission.

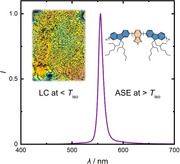

## 1 Introduction

Fluorene‐based conjugated oligomers and polymers combine strong fluorescence with exceptional optical properties and tunable emission colors, making them versatile candidates for light‐emitting diodes and optically pumped lasers. Conjugated polyfluorenes are typically prepared by step‐growth polycondensation reactions using transition metal catalysts. These reactions produce polymers with broad molecular mass distributions and significant batch‐to‐batch variations.^[^ ^1^, ^2^, ^3^ ^]^ Polymers are challenging to purify, often leading to residual metal catalyst impurities, traces of ligands, leaving groups and end‐groups, and undesired byproducts in the final product.^[^ ^4^, ^5^, ^6^ ^]^ On the contrary, oligofluorenes have well‐defined structures, due to their step‐by‐step synthesis.^[^ ^7^, ^8^, ^9^, ^10^, ^11^ ^]^ Their purification via column chromatography ensures high purity effectively removing metal and organic impurities and residues. Such high‐purity oligofluorenes exhibit high photoluminescence quantum yields *ϕ* and tunable emission, rendering them attractive as alternatives to their polymeric counterparts.^[^ ^12^ ^]^ Fluorene‐based oligomers and polymers exhibit a diverse set of morphological phases and characteristics. For example, polyfluorenes can adopt several metastable α‐ and β‐phases,^[^ ^13^, ^14^, ^15^ ^]^ while oligofluorenes display similar phase behavior.^[^ ^8^, ^16^, ^17^, ^18^, ^19^ ^]^ Furthermore, the morphology of pure oligofluorenes can be tuned from crystalline and amorphous to mesophases by varying the oligomer length or by incorporating suitable alkyl side chains.^[^ ^20^, ^21^, ^22^, ^23^, ^24^ ^]^ The alkyl side chains do not directly influence the luminescent properties of the emitters in solution, but they influence the molecular packing of the molecules in the solid state.^[^ ^25^ ^]^ Typically, longer alkyl side chains decrease the glass transition temperature *T*_g_ and serve as a spacer to prevent intermolecular π‐π stacking and quenching of the excited state.^[^ ^26^, ^27^ ^]^

**Scheme 1:**
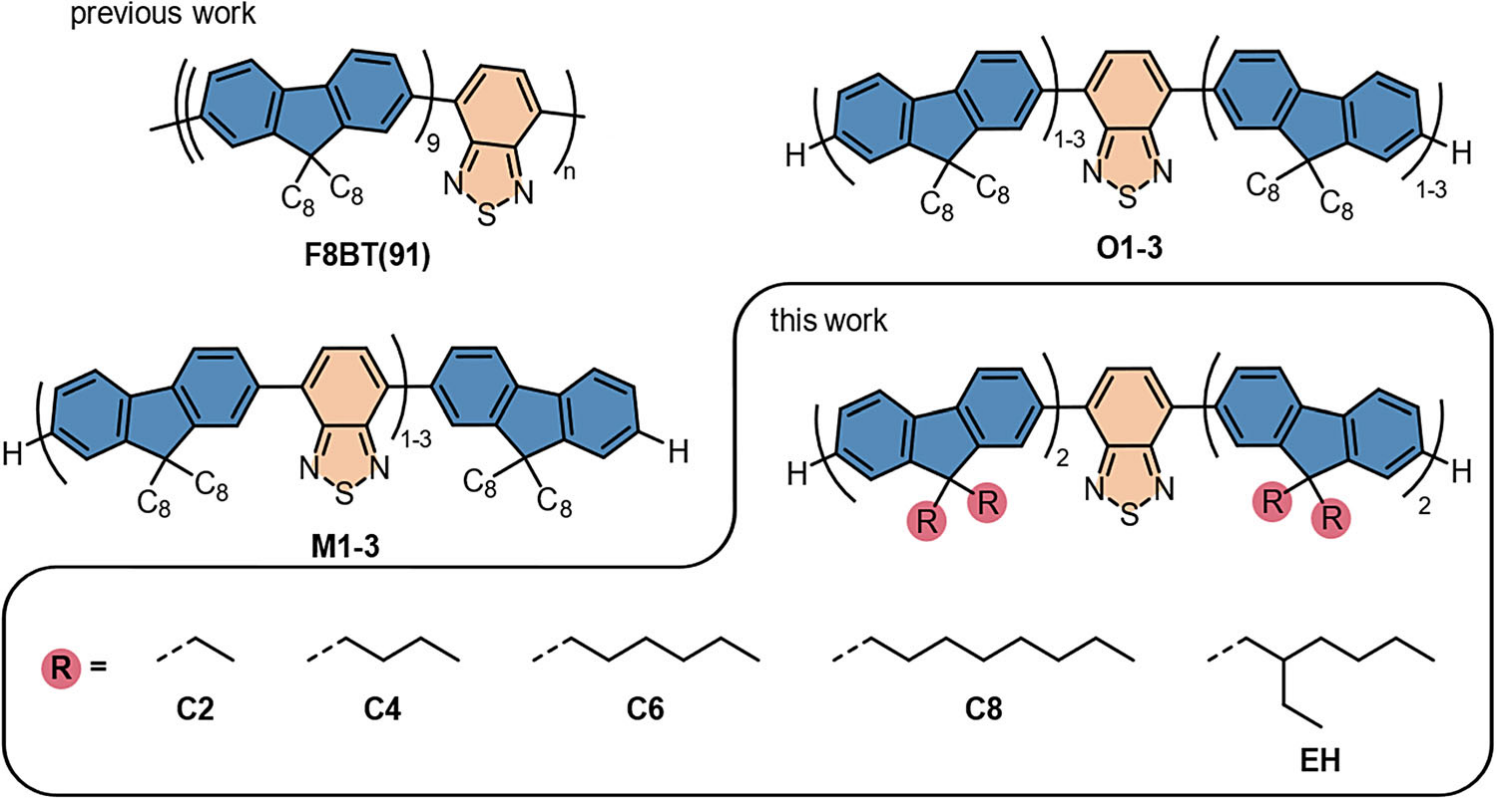
Molecular structures of poly(fluorene‐*co*‐benzothiadiazole) with a comonomer ratio of 9:1, termed **F8BT(91)**. The corresponding oligomers – trimer (**M1**), pentamer (**M2**), and heptamer (**M3**) – consist of alternating fluorene and BT units. Additionally, oligomers with a central BT core flanked by mono‐, bi‐, or trifluorene arms, designated as **O1**, **O2**, and **O3**, respectively, are presented. In this work, we explore the influence of alkyl sidechains (termed **C2**, **C4**, **C6**, **C8**, and **EH**) for pentamers with bifluoryl arms and a BT core.

Therefore, these highly tunable materials provide attractive properties for applications in optoelectronic devices, such as organic light‐emitting diodes (OLEDs) or organic solid‐state lasers (OSSL).^[^ ^8^, ^28^, ^29^, ^30^ ^]^ Using a benzothiadiazole (BT) core together with flanking dioctylfluorene units shifts the luminescence into the yellow spectrum as opposed to the deep blue fluorescence of homofluorene systems. BT‐incorporation can be achieved to yield alternating BT and fluorene units (see M1‐3 in **Scheme** **1**)^[^ ^11^, ^31^ ^]^ or BT can be doped into the fluorene‐based chains at reduced fractions (see F8BT(91) and O1‐3 in Scheme 1).^[^ ^32^, ^33^, ^34^, ^35^, ^36^ ^]^ Both material families (with alternating or doped BT units) offer excellent optical characteristics such as high *ϕ* as well as amplified spontaneous emission (ASE) with thresholds *E*_th_ down to 1.9 µJ cm^−2^. However, these BT‐infused materials lack the variety in morphological phases, as known from homofluorene oligomers and polymers.^[^ ^35^, ^37^ ^]^ Instead, only crystallization is observed for shorter oligomers and amorphous phases for the longer oligomers, but no metastable phases or mesophases are detected. However, in homofluorenes such metastable phases often entail better optoelectronic properties^[^ ^13^, ^14^ ^]^ and liquid crystalline mesophases have been used to realize attractive applications such as in polariton lasers realized in the nematic phase of a polyhomofluorene.^[^ ^38^ ^]^ However, to date, these morphologies appear inaccessible in fluorene‐*co*‐BT oligomers and polymers.

In this work, we make use of the bis(difluorenyl)‐benzothiadiazole pentamer with high *ϕ* and low *E*_th_ and systematically vary the alkyl side‐chain periphery to investigate the influence on the morphology and mesophases. We examine the optical and morphological properties and explore structure‐property relationships. Our fluorene‐BT oligomers exhibit morphological diversity ranging from crystalline and amorphous to liquid crystalline. We record their bright yellow emission in solution and solid‐state and determine their high *ϕ* and low ASE thresholds, with *E*_th_ as low as 1.68 µJ cm⁻^2^.

## 2 Results and Discussion

We start our synthetic endeavor by alkylation of mono‐ and dibrominated fluorenes with ethyl (C2), butyl (C4), hexyl (C6), octyl (C8), and 2‐ethylhexyl (EH) at the 9,9 positions under mild conditions in a base mediated S_N_ reaction. For this purpose, we employ the respective brominated alkanes and obtain the products in quantitative yields (see Supporting Information). The monobrominated fluorenes are then converted to boronic acid esters and subsequent Suzuki‐Miyaura cross coupling reactions between stoichiometric amounts of the monoborylated and dibrominated fluorenes yields the respective bifluorenes. These bifluorenes with different 9,9‐alkyl side chains are then attached to a bis(boronic acid ester) BT in a second Suzuki‐Miyaura reaction, yielding the desired BT‐cored pentamers. An overview scheme and the full synthetic details are provided in the Supporting Information.

Thermogravimetric analysis (TGA) provides insight into the thermal stability of our pentamers. The oligomers exhibit high thermal stability up to around 400 °C, above which degradation occurs (see Figure S2, Supporting Information). The mass loss increases systematically with the increasing length of the alkyl chains, indicating their involvement in the degradation process (for further analysis and discussion see the Supporting Information).

**Figure 1:**
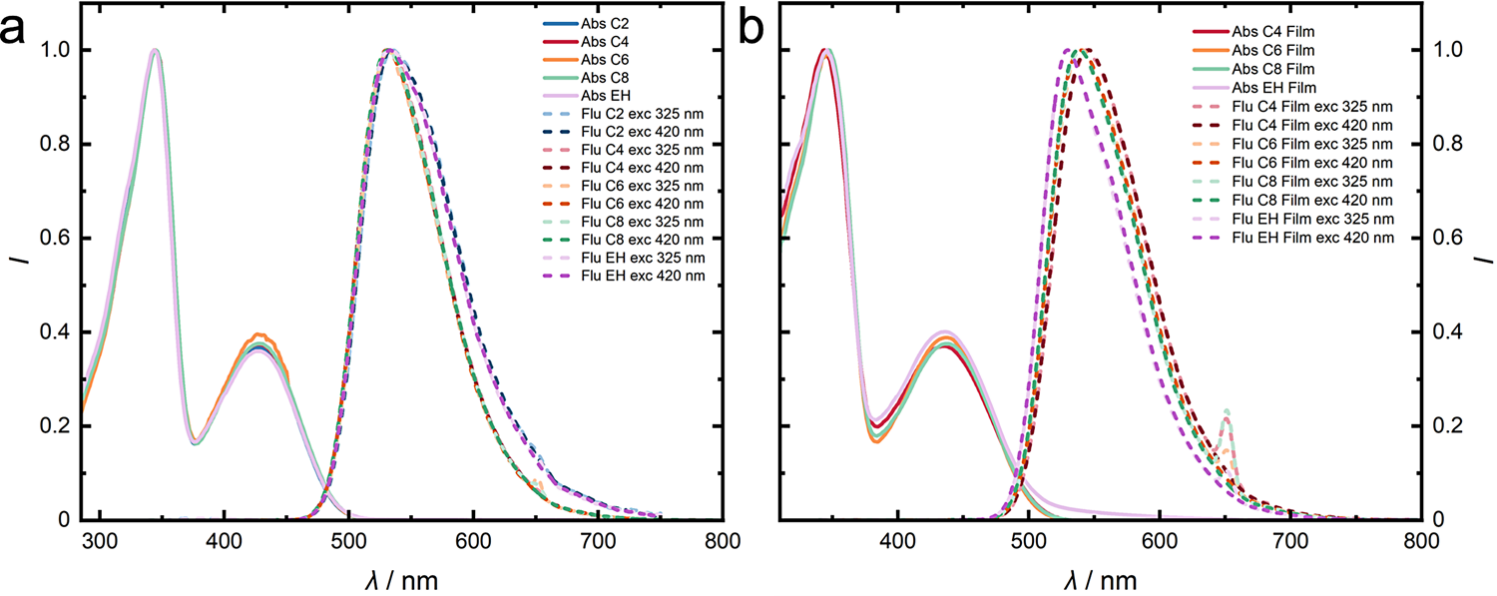
UV–vis absorption (solid lines) and photoluminescence spectra (dashed lines) of the alkylated oligomers in toluene solution (a) and as thin films (b). For photoluminescence, the samples are excited at either 325 or 420 nm, as indicated in the figure legends. The small band at 650 nm represents a remnant of the excitation at 325 nm (twice the excitation wavelength).

To investigate the optical properties, we conduct UV–vis absorption spectroscopy in toluene solution. We observe the same spectral features for all five oligomers with characteristic bifluorene and fluorene‐BT absorption bands at *λ*_A,s_ = 344–345 nm and 426–428 nm, respectively (see **Figure** **1**a and **Table** **1**). When exciting either the bifluorene arms at 325 nm or the conjugated fluorene‐BT moiety at 420 nm,^[^ ^39^ ^]^ almost identical bright yellow emission spectra with maxima between *λ*_PL,s_ = 531–534 nm are observed in all cases (see Figure 1a). This proves complete exciton transfer from excited fluorenes to the BT core. The pentamer solutions exhibit almost identical *ϕ*_s_ for all the different side chains between 87% and 91% (see Table 1). As expected, varying the alkyl substituents does not significantly influence the optoelectronic properties of the oligomers in solution. Yet, we expect a more significant influence of the alkyl chains on the optoelectronic properties and the morphology in the solid state, while the absorption profiles of thin films of our pentamers are again very similar (see Figure 1b and Table 1).

**Table 1:** Molecular analytical and spectroscopic characteristics of our conjugated oligomers.

compound	substituent	*λ*_A,s_ /nm	*λ*_PL,s_ /nm	*λ*_A,f_ /nm	*λ*_PL,f_ /nm	*T*_g_ or *T*_iso_ /°C	*ϕ*_s_	*ϕ*_f_	*R*_a_ /nm	*E*_th_ /µJ cm^−2^	*λ*_ASE_ /nm	*FWHM* /nm
C2	Ethyl	344, 426	534	–	–	–	0.89	–	–	–	–	–
C4	Butyl	345, 428	531	344, 435	545	104.1	0.91	0.54	0.32	1.99^b)^ 2.33^c)^	565	16.6^d)^
C6	Hexyl	345, 428	531	346, 437	539	55.8	0.90	0.62	0.20	1.64^b)^ 3.00^c)^	561	18.2^d)^
C8	Octyl	345, 428	532	347, 437	538	19.8	0.87	0.85	0.28	1.80^b)^ 1.46^c)^	555	17.2^d)^
EH	2‐Ethylhexyl	344, 427	533	346, 436	530	0.27, 43.8^a)^	0.91	0.85	0.28	8.47^b)^ 7.40^c)^	553	31.4^d)^

^a)^ This value refers to the transition from the nematic mesophase to the isotropic phase;

^b)^ Determined by the intersection of intensity linear fits;

^c)^ Determined by the intersection of *FWHM* linear fits;

^d)^ At *E*_P_ of 20.5 µJ cm^−2^. Values for C8 taken from Ref. [35].

*λ*_A,s_: maximum absorption wavelength in toluene, *λ*_PL,s_: maximum photoluminescence wavelength in toluene, *λ*_A,f_: maximum absorption wavelength of thin films, *λ*_PL,f_: maximum photoluminescence wavelength of thin films, *T*_g_: glass transition temperature, *T*_iso_: nematic‐isotropic phase transition temperature, *ϕ*_s_: photoluminescence quantum yield in toluene solution, *ϕ*_f_: photoluminescence quantum yield of thin films, *R*_a_: surface roughness of thin films, *E*_th_: ASE threshold, *λ*_ASE_: maximum wavelength of the ASE signal, *FWHM*: Full width at half maximum of the ASE spectra.

**Figure 2:**
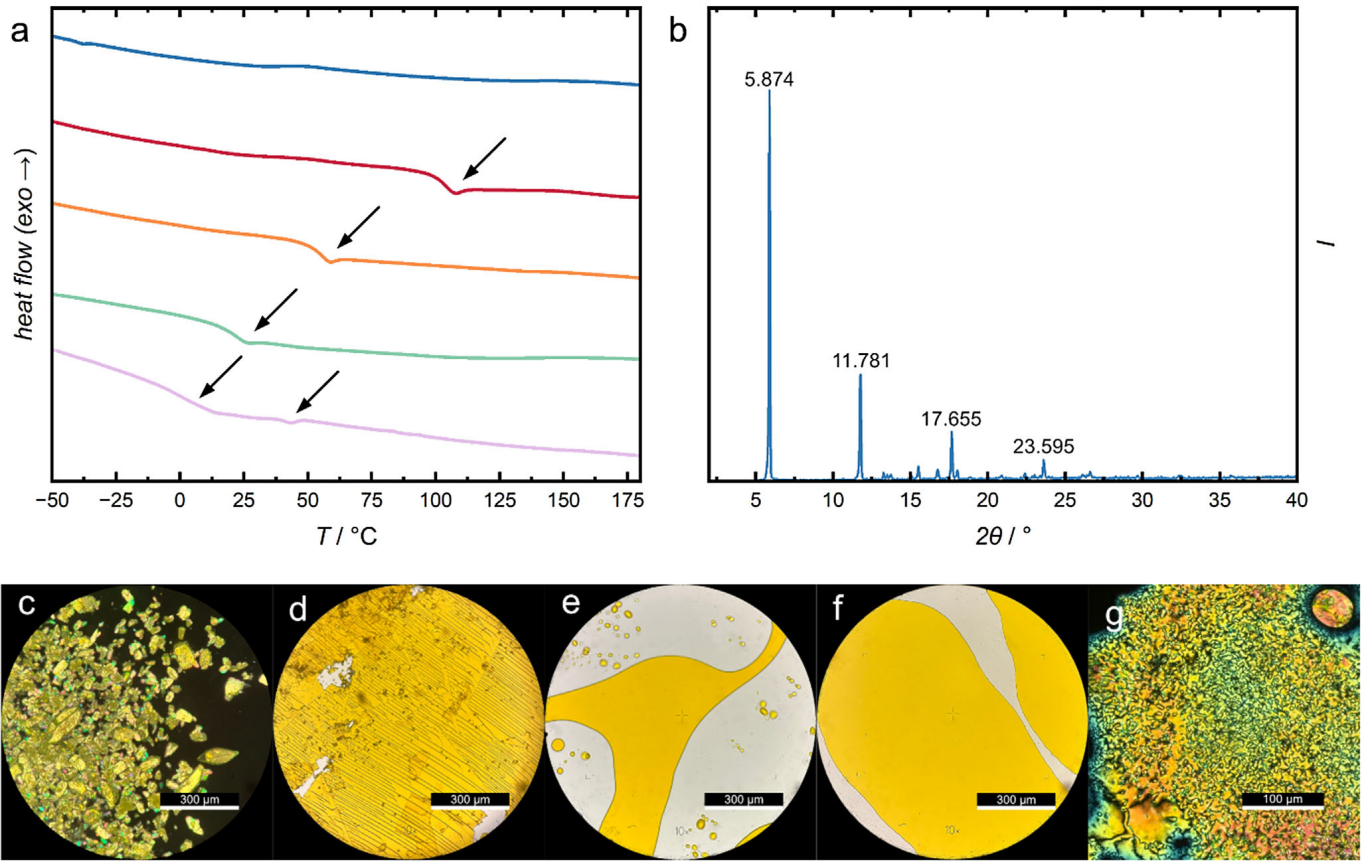
a) Differential scanning calorimetry (DSC) of C2 (blue), C4 (red), C6 (orange), C8 (green), and EH (purple) at heating rates of 15 K min^−1^. The second heating curves are shown after the thermal history has been erased in the first cycle. b) Powder X‐ray diffractogram of C2. POM images of C2 (c), C4 (d), C6 (e), C8 (f) and EH (g) at room temperature after an initial annealing step at 150 °C. For C4, C6, and C8 the polarization filters are aligned in parallel, while C2 and EH reveal birefringence with crossed polarization filters.

Only the EH pentamer film shows a broadened BT absorption band with a small shoulder extending into the red region. This shoulder could be an effect of scattering from a morphological feature. In contrast to the steady behavior of the photoluminescence in solution, the photoluminescence maxima of the films (*λ*_PL,f_) shift hypsochromically for increasing alkyl chain lengths (see Figure 1b and Table 1). The *λ*_PL,f_ decreases from 545 nm for C4 to 538 nm for C8. This could be explained by interchain interactions, where shorter alkyl chains allow the conjugated chains to pack more closely in a certain morphology. To corroborate this claim, we perform differential scanning calorimetry (DSC) of our different samples (see **Figure** **2a**). The pentamer with short C2 ethyl side chains is crystalline and shows no melting in the temperature range up to 250 °C (see the Supporting Information for all cycles across the entire temperature range). The pentamer with C4 butyl chains exhibits a glass transition *T*_g_ at 104.1 °C and a melting point *T*_m_ at 226 °C with corresponding cold crystallization at 202 °C. Observing both *T*_g_ and *T*_m_ implies that C4 exhibits amorphous and crystalline domains. Further elongation to C6 hexyl and C8 octyl side chains leads to purely amorphous materials while decreasing the *T*_g_ of our pentamers to 55.8 and 19.8 °C, respectively. Branching of the octyl chains to EH 2‐ethylhexyl, further reduces the *T*_g_ to 0.3 °C, while an additional endothermic signal arises at *T*_iso_ = 43.8 °C (see arrows in Figure 2a). This latter transition hints at a melting event possibly from a mesophase to an isotropic phase with a specific enthalpy of *H*_iso_ = 0.287 kJ mol^−1^, which is typical for nematic to isotropic phase transitions.^[^ ^40^ ^]^ To take a deeper look into the alkyl chain movement at varying temperatures, we perform solid‐state ^1^H‐NMR measurements. We observe the rise of additional alkyl signals above the *T*_g_ and the sharpening of these signals above *T*_iso_ (See Figure S6, Supporting Information and the following discussion in the Supporting Information). We conduct X‐ray diffractometry for a more detailed look into potential crystal structures. Only C2 shows reflections (see Figure 2b), while all other compounds show only amorphous scattering halos. The pattern in the diffractogram of the C2 pentamer can be analyzed using Bragg's equation delivering the diffraction order *n* allowing us to determine the crystal lattice distance *d* = 1.50 nm (see Equation S1, Supporting Information). To take an in‐depth look into the morphology and the crystalline and amorphous phases, we monitor the samples each using a polarization microscope during a temperature ramp from 25 to 180 °C. As expected, C2 exhibits stable yellow birefringent crystals that do not melt in the chosen temperature interval (see Figure 2c). The C4, C6, and C8 pentamers exhibit amorphous morphology at room temperature, without any apparent birefringence visible through crossed polarization filters (see Figure 2d,e,f with polarization filters aligned parallel). C4 behaves like a brittle glass at room temperature, while C8 is a viscous oil, which is in line with the determined *T*_g_ from the DSC measurements discussed above. By contrast, the EH functionalized pentamer shows birefringence with a Schlieren texture at room temperature. The Schlieren texture clears at 44 °C into a phase without birefringence and reoccurs during cooling of the sample. This phase transition is fully reversible and in accordance with the DSC measurement. We attribute this behavior to the clearing of a nematic liquid crystalline phase into an isotropic melt, consistent with the above‐determined *H*_iso_, the observed Schlieren texture, and previous observations reported for pure oligofluorenes.^[^ ^20^, ^22^, ^41^, ^42^, ^43^ ^]^ Interestingly, its constitutional isomer – the C8 pentamer – does not exhibit thermotropic liquid crystalline behavior (see Figure 2f). We attribute this to the influence of the branched 2‐ethylhexyl group in contrast to the linear octyl chain. Branching alkyl chains seems to better induce liquid crystalline phases in oligofluorenes, probably because of less side‐chain interdigitation and a more calamitic shape of the pentamers.^[^ ^20^, ^22^ ^]^ Accordingly, pure pentafluorenes with 2‐ethylhexyl side chains also exhibit a nematic phase with a *T*_g_ = 29 °C and *T*_iso_ = 126 °C.^[^ ^20^, ^24^ ^]^

To test the oligomers for their potential for optical amplification, we spin‐coat thin films of the oligomers with thicknesses of around 280–320 nm on glass substrates. Only for the C2 pentamer, we are unable to prepare films with reasonable thicknesses, due to the limited solubility of the pentamer with the short ethyl side groups.

**Figure 3:**
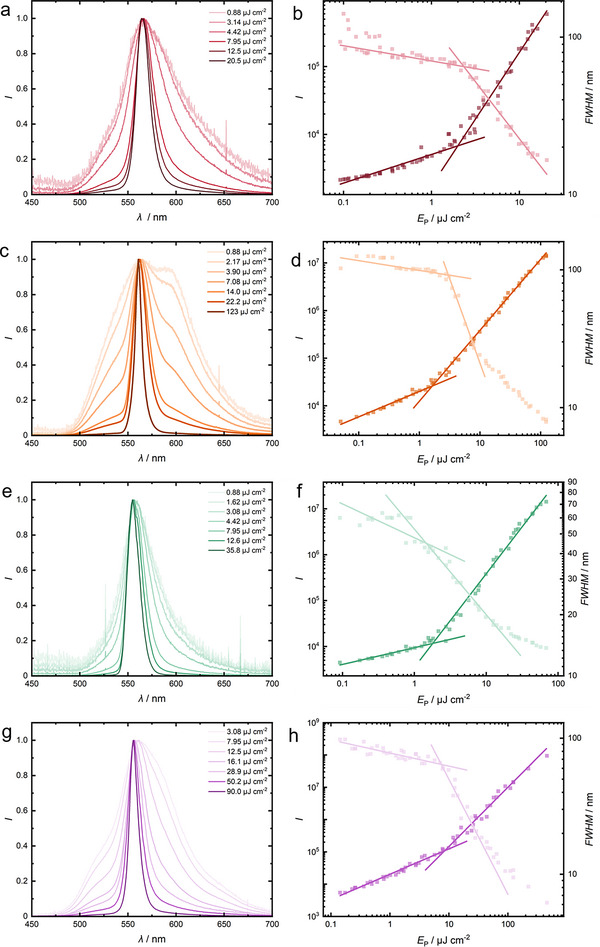
Amplified spontaneous emission (ASE) measurements of solid films of the respective oligomers. Normalized emission spectra with varying *E*_P_ of pentamers C4 (a), C6 (c), C8 (e) and EH (g). Plots of output intensity *I* (dark squares, left axis) and emission linewidth as *FWHM* (light squares, right axis) versus *E*_P_ for C4 (b), C6 (d), C8 (f), and EH (h). The data segments are linearly fitted, and the intersection points of the fits indicate the ASE thresholds. Values for C8 taken from Ref. [35].

All prepared films exhibit smooth surfaces with similar roughnesses *R*_a_ between 0.32 and 0.20 nm as determined by atomic force microscopy (AFM) (see Figure S5, Supporting Information). The *ϕ*_f_ of the films increases with the length of the alkyl side chain, rising from 54% for C4 over 62% for C6 and reaching 85% for C8. As expected, *ϕ*_f_ remains constant at 85% for its constitutional isomer with EH chains (see Table 1). This behavior suggests again that shorter alkyl chains promote intermolecular (charge transfer and quenching) interactions within the films, thereby reducing *ϕ*. We pump the oligomer films using a laser beam shaped into a stripe (355 nm, 5–7 ns, 20 Hz) and record the emission spectra perpendicular to the film edge to capture the waveguided emission (see Figure S1, Supporting Information for the used setup). By varying the pump energy *E*_P,_ we test the materials for stimulated emission. At low *E*_P_, we observe broad emission similar to the fluorescence spectra of the films (cf. spectra in Figures 1b and 3). However, when increasing the energy, the emission bands significantly narrow and drastically increase in intensity (see **Figure** **3**a,c,e).

We attribute this spectral narrowing and increase in intensity to amplified spontaneous emission (ASE). By plotting the intensity and the full width at half maximum *FWHM* against *E*_P_, we obtain trends that can be fitted with two linear branches. The intersection of the two linear segments allows us to determine the ASE threshold *E*_th_ (see Figure 3b,d,f). For the intensity fits, the threshold of all compounds is nearly identical and lies between 1.64 and 1.99 µJ cm^−2^, which is in accordance with previously reported BT‐containing oligofluorenes (alternating and with C8 side‐chains).^[^ ^31^, ^35^ ^]^ Only for the EH pentamer we do not observe ASE at room temperature. However, when we heat the film to above the nematic‐isotropic phase transition temperature *T*_iso_, we observe the same behavior as with the other amorphous films with C4, C6, and C8 side chain lengths (see Figure 3g,h). Plotting *I* and *FWHM* against *E*_P_ lets us again determine *E*_th_ for the EH pentamer and we record *E*_th_ = 8.47 µJ cm^−2^, slightly above the threshold of the other BT oligomers and even its constitutional isomer C8 (see Table 1). This increased *E*_th_ might be attributed to the elevated temperatures, which may support the nonradiative decay pathway, such as vibrational relaxation. The stimulated emission of our EH pentamer can be turned on and off fully reversibly upon heating or cooling. We perform temperature‐dependent measurements of the spectra and observe a significant increase in the intensity and narrowing of the *FWHM* near and beyond *T*_iso_, which confirms that this phase transition is responsible for the presence of ASE (see Figure S7, Supporting Information). The alkyl chain length of C4 to C8 seems not to influence *E*_th_ significantly, only the presence of the liquid crystalline phase in EH appears to increase *E*_th_ or even inhibit ASE. We hypothesize that grain boundaries in the liquid crystalline phase inhibit the waveguiding of the stimulated emission which suppresses ASE. Compared to the amorphous films C4 – C8, the EH film is slightly turbid (see comparison in Figure S4, Supporting Information). The emission wavelength of the ASE spectra *λ*_ASE_ shifts slightly hypsochromically from 565 nm for C2 to 553 nm for EH, which is in accordance with the emission maxima of the photoluminescence spectra of the thin films. The *FWHM* at *E*_P_ = 20.5 µJ cm^−2^ is similar between 16.6 and 18.2 nm for C4, C6 and C8. Only for EH, the *FWHM* is at 31.4 nm at this pump energy, which results from the higher *E*_th_.

## 3 Conclusion

This work explores the influence of various alkyl substituents on the morphological properties of bisdifluorene‐BT pentamers as potential laser gain materials. Using branched alkyl chains enables the formation of liquid crystalline phases at room temperature. ASE can be obtained by heating above the nematic‐isotropic transition temperature and will vanish again upon cooling. Due to the high *ϕ* and low *E*_th_, the presented materials offer high potential to be used in optoelectronic devices, such as OLEDs or novel organic laser geometries.

## Conflict of Interest

The authors declare no conflict of interest.

## Supporting information



Supporting Information

## Data Availability

The data that support the findings of this study are available from the corresponding author upon reasonable request.

## References

[1] U. Scherf , E. J. W. List , Adv. Mater. 2002, 14, 477.

[2] K. Hosoi , T. Mori , T. Mizutani , T. Yamamoto , N. Kitamura , Thin Solid Films 2003, 438–439, 201.

[3] M. Knaapila , R. Stepanyan , M. Torkkeli , B. P. Lyons , T. P. Ikonen , L. Almásy , J. P. Foreman , R. Serimaa , R. Güntner , U. Scherf , A. P. Monkman , Phys. Rev. E 2005, 71, 041802. 10.1103/PhysRevE.71.041802 15903692

[4] J. Frahn , B. Karakaya , A. Schäfer , A. D. Schlüter , Tetrahedron 1997, 53, 15459.

[5] M. Y. C. Ting , L. P. E. Yunker , I. C. Chagunda , K. Hatlelid , M. Vieweg , J. S. McIndoe , Catal. Sci. Technol. 2021, 11, 4406.

[6] J. Sakamoto , M. Rehahn , G. Wegner , A. D. Schlüter , Macromol. Rapid Commun. 2009, 30, 653. 21706656 10.1002/marc.200900063

[7] Q. Wang , Y. Qu , H. Tian , Y. Geng , F. Wang , Macromolecules 2011, 44, 1256.

[8] L. S. Chinelatto , J. del Barrio , M. Piñol , L. Oriol , M. A. Matranga , M. P. De Santo , R. Barberi , J. Photochem. Photobiol. A Chem. 2010, 210, 130.

[9] R. Anémian , J. C. Mulatier , C. Andraud , O. Stéphan , J. C. Vial , Chem. Commun. 2002, 15, 1608. 10.1039/b201414a 12170805

[10] Y. Koizumi , S. Seki , A. Acharya , A. Saeki , S. Tagawa , Chem. Lett. 2004, 33, 1290.

[11] C. R. Belton , A. L. Kanibolotsky , J. Kirkpatrick , C. Orofino , S. E. T. Elmasly , P. N. Stavrinou , P. J. Skabara , D. D. C. Bradley , Adv. Funct. Mater. 2013, 23, 2792.

[12] E. Y. Choi , L. Mazur , L. Mager , M. Gwon , D. Pitrat , J. C. Mulatier , C. Monnereau , A. Fort , A. J. Attias , K. Dorkenoo , J. E. Kwon , Y. Xiao , K. Matczyszyn , M. Samoc , D.‐W. Kim , A. Nakao , B. Heinrich , D. Hashizume , M. Uchiyama , S. Y. Park , F. Mathevet , T. Aoyama , C. Andraud , J. W. Wu , A. Barsellad , J. C. Ribierre , Phys. Chem. Chem. Phys. 2014, 16, 16941. 25005146 10.1039/c4cp01134a

[13] M. Grell , D. D. C. Bradley , G. Ungar , J. Hill , K. S. Whitehead , Macromolecules 1999, 32, 5810.

[14] S. H. Chen , A. G. Su , C. H. Su , S. A. Chen , Macromolecules 2005, 38, 379.

[15] M. N. Yu , H. Soleimaninejad , J. Y. Lin , Z. Y. Zuo , B. Liu , Y. F. Bo , L. B. Bai , Y. M. Han , T. A. Smith , M. Xu , X. P. Wu , D. E. Dunstan , R. D. Xia , L. H. Xie , D. D. C. Bradley , W. Huang , J. Phys. Chem. Lett. 2018, 9, 364. 29298074 10.1021/acs.jpclett.7b03148

[16] C. Chi , G. Lieser , V. Enkelmann , G. Wegner , Macromol. Chem. Phys. 2005, 206, 1597.

[17] W. C. Tsoi , D. G. Lidzey , J. Phys. Condens. Matter 2008, 20, 125213.

[18] W. C. Tsoi , A. R. Buckley , D. G. Lidzey , Chem. Phys. Lett. 2009, 468, 32.

[19] Y. Deng , W. Yuan , Z. Jia , G. Liu , J. Phys. Chem. B 2014, 118, 14536. 25402824 10.1021/jp510520m

[20] J. Jo , C. Chi , S. Höger , G. Wegner , D. Y. Yoon , Chem. Eur. J. 2004, 10, 2681. 15195299 10.1002/chem.200305659

[21] Y. Geng , A. Trajkovska , D. Katsis , J. J. Ou , S. W. Culligan , S. H. Chen , J. Am. Chem. Soc. 2002, 124, 8337. 12105915 10.1021/ja026165k

[22] Y. Geng , S. W. Culligan , A. Trajkovska , J. U. Wallace , S. H. Chen , Chem. Mater. 2003, 15, 542.

[23] M. J. Xiong , Z. H. Li , M. S. Wong , Aust. J. Chem. 2007, 60, 608.

[24] P. Papadopoulos , G. Floudas , C. Chi , G. Wegner , J. Chem. Phys. 2004, 120, 2368. 15268376 10.1063/1.1637339

[25] O. D. Bernardinelli , G. C. Faria , L. A. De Oliveira Nunes , R. M. Faria , E. R. Deazevedo , M. F. S. Pinto , J. Phys. Chem. A 2012, 116, 4285. 22471613 10.1021/jp210953m

[26] C. Orofino , A. L. Kanibolotsky , P. J. Skabara , Arkivoc 2021, 2021, 268.

[27] P. O. Morawska , Y. Wang , A. Ruseckas , C. Orofino‐Penia , A. L. Kanibolotsky , R. Santhanagopal , N. Fröhlich , M. Fritsch , S. Allard , U. Scherf , P. J. Skabara , I. D. W. Samuel , G. A. Turnbull , J. Phys. Chem. C 2015, 119, 22102.

[28] K. S. Daskalakis , S. A. Maier , R. Murray , S. Kéna‐Cohen , Nat. Mater. 2014, 13, 271. 24509602 10.1038/nmat3874

[29] D. Xia , C. Duan , S. Liu , D. Ding , M. Baumgarten , M. Wagner , D. Schollmeyer , H. Xu , K. Müllen , New J. Chem. 2019, 43, 3788.

[30] Y. Wang , G. Tsiminis , Y. Yang , A. Ruseckas , A. L. Kanibolotsky , I. F. Perepichka , P. J. Skabara , G. A. Turnbull , I. D. W. Samuel , Synth. Met. 2010, 160, 1397.

[31] M. Mamada , R. Komatsu , C. Adachi , ACS Appl. Mater. Interfaces 2020, 12, 28383. 32453542 10.1021/acsami.0c05449

[32] R. Xia , G. Heliotis , Y. Hou , D. D. C. Bradley , Org. Electron. 2003, 4, 165.

[33] R. Xia , G. Heliotis , P. N. Stavrinou , D. D. C. Bradley , Appl. Phys. Lett. 2005, 87, 031104.

[34] A. J. C. Kuehne , M. C. Gather , Chem. Rev. 2016, 116, 12823. 27501192 10.1021/acs.chemrev.6b00172

[35] P. J. Welscher , U. Ziener , A. J. C. Kuehne , Macromolecules 2025, 58, 2730.

[36] D. Amarasinghe , A. Ruseckas , A. E. Vasdekis , G. A. Turnbull , I. D. W. Samuel , Adv. Mater. 2009, 21, 107.

[37] M. J. Banach , R. H. Friend , H. Sirringhaus , Macromolecules 2004, 37, 6079.

[38] F. Le Roux , A. Mischok , F. Tenopala‐Carmona , M. C. Gather , Nat. Commun. 2025, 16, 811. 39827165 10.1038/s41467-025-55875-1 PMC11743153

[39] J. C. Denis , A. Ruseckas , G. J. Hedley , A. B. Matheson , M. J. Paterson , G. A. Turnbull , I. D. W. Samuel , I. Galbraith , Phys. Chem. Chem. Phys. 2016, 18, 21937. 27439750 10.1039/c6cp02059c

[40] W. E. Acree , J. S. Chickos , J. Phys. Chem. Ref. Data 2006, 35, 1051.

[41] T. Yasuda , K. Fujita , T. Tsutsui , Y. Geng , S. W. Culligan , S. H. Chen , Chem. Mater. 2005, 17, 264.

[42] S. W. Culligan , Y. Geng , S. H. Chen , K. Klubek , K. M. Vaeth , C. W. Tang , Adv. Mater. 2003, 15, 1176.

[43] A. Trajkovska , C. Kim , J. U. Wallace , S. H. Chen , Liq. Cryst. XI 2007, 6654, 665409.

