# Supplementary material for: Liquid Crystalline Fluorene‐2,1,3‐Benzothiadiazole Oligomers with Amplified Spontaneous Emission
Source: Macromol Rapid Commun. 2025 Apr 14;46(16):2500189. doi: 10.1002/marc.202500189 (PMC12360127; doi:10.1002/marc.202500189)
Supplement: Supplementary file 1 — Supporting Information [file MARC-46-2500189-s001.pdf]

Macromolecular Rapid Communications

Supporting Information

for *Macromol. Rapid Commun.*, DOI 10.1002/marc.202500189

Liquid Crystalline Fluorene-2,1,3-Benzothiadiazole Oligomers with Amplified Spontaneous Emission

*Philipp J. Welscher, Ulrich Ziener and Alexander J. C. Kuehne**

Supporting Information

Liquid Crystalline Fluorene-2,1,3-Benzothiadiazole Oligomers with Amplified Spontaneous Emission

*Philipp J. Welscher, Ulrich Ziener and Alexander J. C. Kuehne**

General

All reagents are purchased from commercial suppliers. The solvents petroleum ether and dichloromethane are distilled prior to usage, otherwise all reagents are used as received. The starting materials 2,7-dibromo-9,9-dihexyl-9H-fluorene, 2-bromo-9,9-dihexyl-9H-fluorene, 2,7-dibromo-9,9-dioctyl-9H-fluorene, 2-(9,9-dioctyl-9H-fluoren-2-yl)-4,4,5,5-tetramethyl-1,3,2-dioxaborolane are available and purchased from commercial suppliers. All syntheses are carried out under nitrogen atmosphere.

Absorption spectroscopy

Absorption measurements are performed on a Perkin Elmer Lambda 365.

Fluorescence spectroscopy

Fluorescence spectra are recorded on a Perkin Elmer FL 6500.

Photoluminescence quantum yield (PLQY)

Photoluminescence quantum yield of solutions and films is measured with a Hamamatsu Quantaurus.

Mass spectrometry

The MALDI and APCI mass spectra are obtained on a FT-ICR-SolariX spectrometer from Bruker Daltonik GmbH. The used matrix is trans-2-[3-(4-tert-butylphenyl)-2-methyl-2-propenyl-idene]malononitrile (DCTB).

NMR spectroscopy

The NMR spectra (each time referenced against the solvent peak of $CDCl_3$) are measured with an AMX 400 or an AMX 600 spectrometer from Bruker. The data is assessed and evaluated with MestReNova (Mestrelab Research). In ^{1}H-NMR, the remaining solvents dichloromethane, acetone and water can be assigned to signals at 5.30, 2.17 and 1.54 ppm, respectively. In ^{13}C-NMR, the remaining solvent acetone can be assigned to the signal at 31.08 ppm. The tetramethylsilane (TMS) signal is visible at 0.0 ppm in ^{1}H-NMR.

Differential scanning calorimetry (DSC)

Differential scanning calorimetry is measured on a DSC 2 from Mettler Toledo with the STARe Software V.13 in 40 µl Al-pans under nitrogen atmosphere. The glass transition temperature T_g is measured by determining the inflection point of the respective signal in DSC.

Thermogravimetric analysis (TGA)

TGA-measurements are done with a TGA 8000 TM from Perkin Elmer under nitrogen atmosphere with a temperature gradient of 10 K min^{-1}.

X-Ray Diffractometry (XRD)

X-Ray Diffractograms are performed using a X´Pert MPD Pro from PANalytical with Cu Kα radiation and a X´Cellerator. The samples are prepared by drop casting a suspension of C2 and toluene (30 mg ml^{-1}) onto a glass substrate. Prior to use, the glass substrate is cleaned in deionized water, isopropanol, and acetone by ultrasonication for 10 min each.

The crystal lattice distance *d* is calculated by using the Bragg's law:

$$n\lambda = 2dsin\theta \qquad (1)$$

where *n* is the diffraction order, λ is the wavelength of the X-Ray beam and 2θ gives the glancing angle.

Table S1: Overview of determined crystal lattice distance

n	2θ / °	*d* / nm
1	5.874	1.50
2	11.781	1.50
3	17.655	1.51
4	23.595	1.51

Spincoating

The oligomers are spin-coated onto square glass slides with the dimensions 24x24 mm size #5 from ORSAtec GmbH with a Laurell Model WS-400B-6NPP/LITE/8K. Prior to coating, the glass slides are cleaned in deionized water, isopropanol and acetone in an ultrasonic bath for 10 min each.

Atomic Force Microscopy (AFM)

AFM images are acquired in tapping mode using a Multimode-AFM NanoScope® IIIa from Veeco Instruments Inc. (Digital Instruments) and recorded with the NanoScope V.5.30r.sr3 software.

Polarization microscopy (POM)

POM pictures are taken with a Carl Zeiss Axioskop 50 with a magnification of 200x for Figures 2c-h and 600x for Figure 2g.

Amplified Spontaneous emission (ASE) setup

The same custom setup described previously[1] is employed to measure and quantify amplified spontaneous emission (ASE), as depicted in Figure S1. An OpoletteUX Nd:YAG-pumped laser system from Opotek operating at a wavelength of 355 nm with a pulse duration of 5–7 ns, serves as excitation source. The pump energy E_P is varied during the measurements using a combination of a rotatable half-wave plate, a polarized plate beamsplitter, and neutral density (ND) filters. The laser beam is subsequently expanded, then refocused using a cylindrical lens, and shaped with a slit to form a rectangular excitation area of 6 mm^2 on the sample. Emitted light is collected at a 90° angle and directed to an iDus 420A-BN detector from Andor Technology. For the temperature dependent ASE measurement, the film is heated to 85 °C, then the heating source is removed, and the film is allowed to cool to room temperature, while simultaneously measuring the emission. The temperature T of the film is determined in two points and the intermediate temperatures are fitted using Newton’s law of cooling.

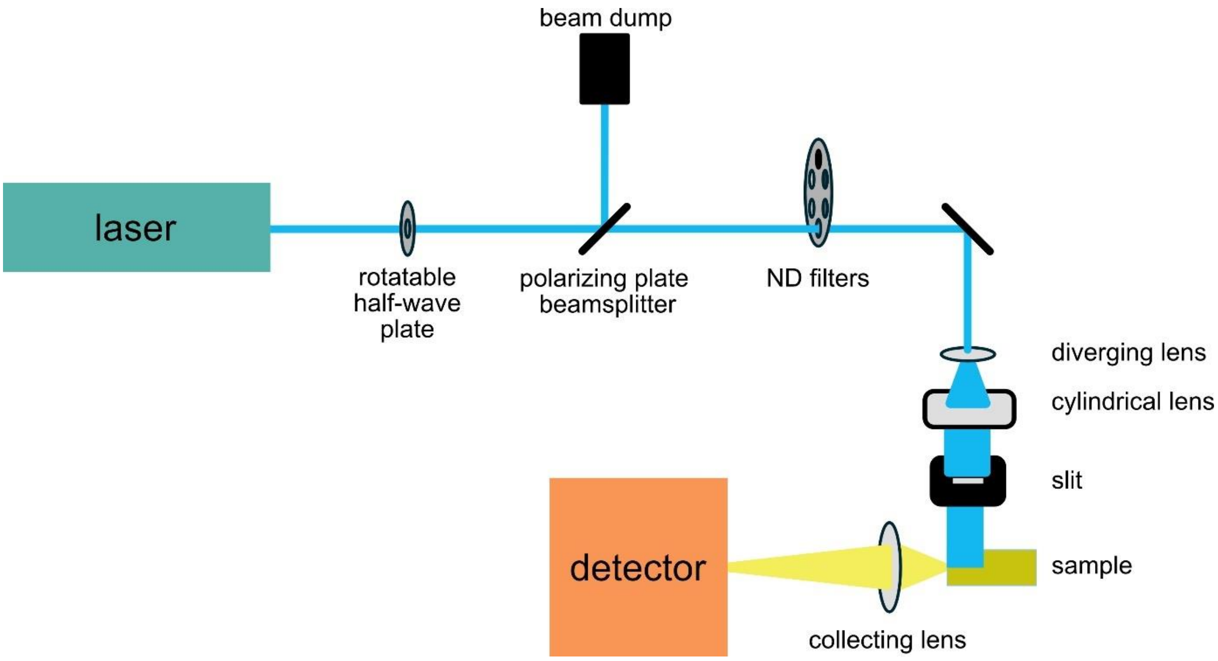

Figure S1: Laser setup to measure amplified spontaneous emission (ASE) from a solid film sample. Reproduced under terms of the CC-BY 4.0 license. Welscher, P. J.; Straub, D.; Stümpges, F.; Respondek, A. L.; Esser, B.; Kuehne, A. J. C. Electron Donor-Functionalized Pyrenes with Amplified Spontaneous Emission for Violet–Blue Electroluminescent Devices Beyond the Spin Statistical Limit. *Adv. Funct. Mater*. Published by Wiley-VCH **2024**, 2417129. https://doi.org/10.1002/adfm.202417129.[1]

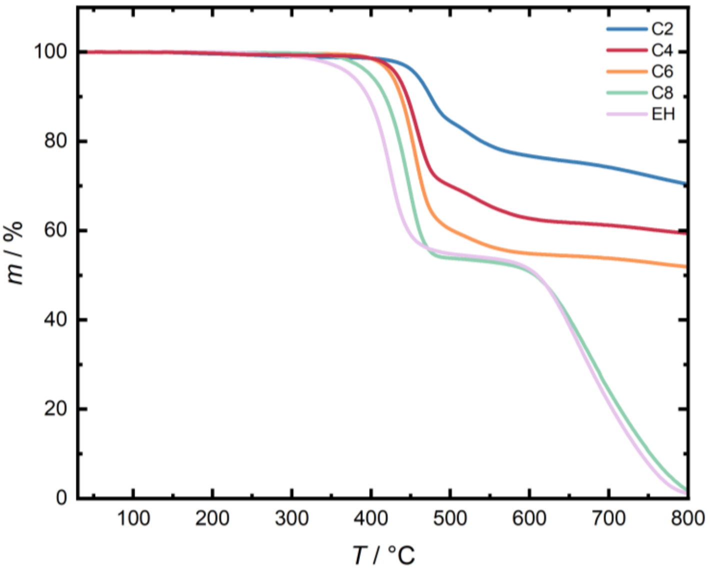

Figure S2: Thermogravimetric analysis (TGA) of the synthesised oligomers under nitrogen atmosphere with a heating rate of 10 °C min^{-1}.

The mass loss increases systematically with increasing length of the alkyl chains, indicating their involvement in the degradation process. For C2, C4, and C6 two processes are identified, respectively at around 480 °C and at 560 °C. The mass loss at 480 °C refers to a BT-

pentafluorene with only eight methyl groups (C1), and the mass loss at 560 °C to a BT-pentafluorene without any alkyl chains (C0) (see Table S2). For these three compounds the aromatic part seems to be stable during the whole measurement. For the traces C8 and EH, after the formation of C1, the compounds including the aromatic parts begin to completely degrade without stabilization of the C0 form. We hypothesize that the aromatic pentamer backbone is packed more densely in the solid state for molecules with shorter C2, C4, C6 alkyl side chains, which upon heating leads to the formation of carbonaceous residues. The longer side chains in the C8 and EH-functionalized pentamers will prevent this close packing allowing for complete degradation, leaving no residues.

Table S2: Comparison of the mass loss as determined from TGA and theoretical molecular weights

compound	*m* at 480 °C / %	$M_{C1} \cdot (M_{compound})^{-1}$ / %	*m* at 560 °C / %	$M_{C0} \cdot (M_{compound})^{-1}$ / %
C2	88.4	89.0	78.3	77.9
C4	72.3	72.9	64.7	63.9
C6	63.2	61.7	56.0	54.1
C8	54.6	53.5	52.8	46.9
EH	55.5	53.5	53.6	46.9

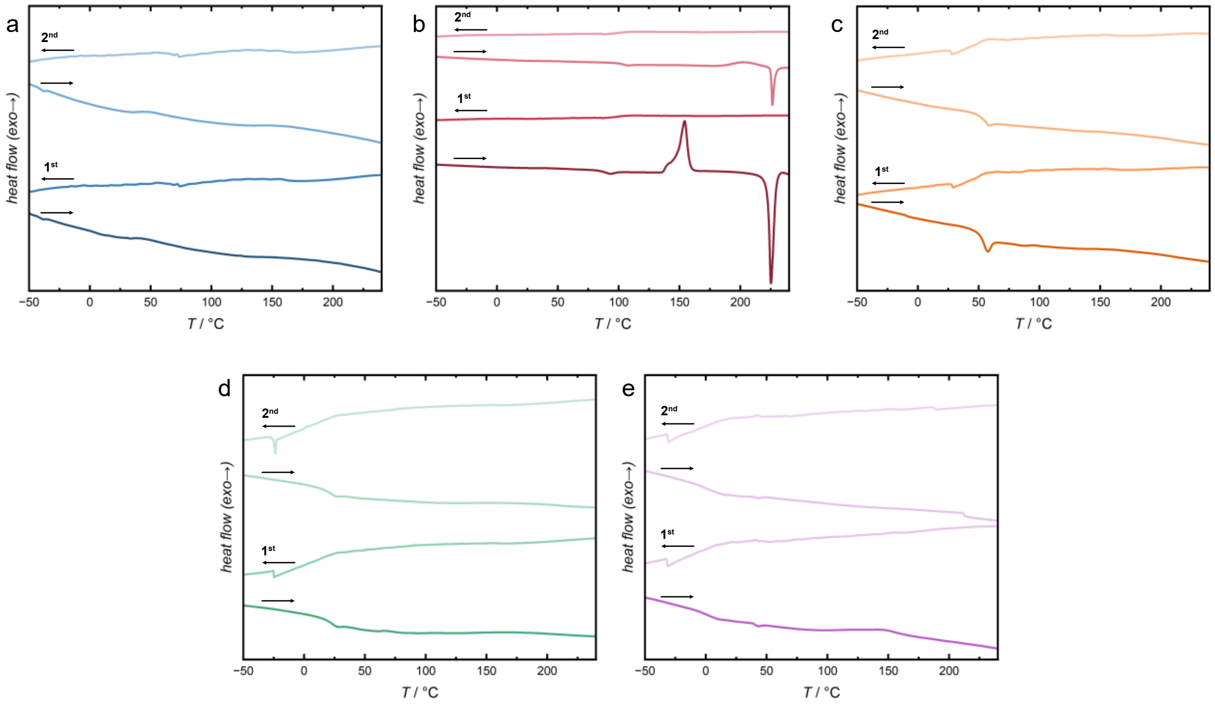

Figure S3: Differential scanning calorimetry (DSC) measurements of C2 (a), C4 (b), C6 (c), C8 (d) and EH (e) under nitrogen atmosphere. The first two heating and cooling cycles are shown. All heating traces are measured with 15 K min^{-1}.

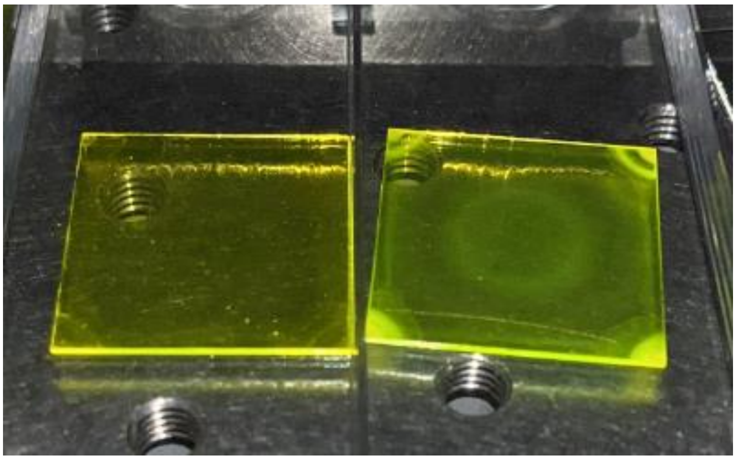

Figure S4: Photograph of the films of C8 (left) and EH (right). The amorphous C8 shows a clear film, while the liquid crystalline EH film is turbid.

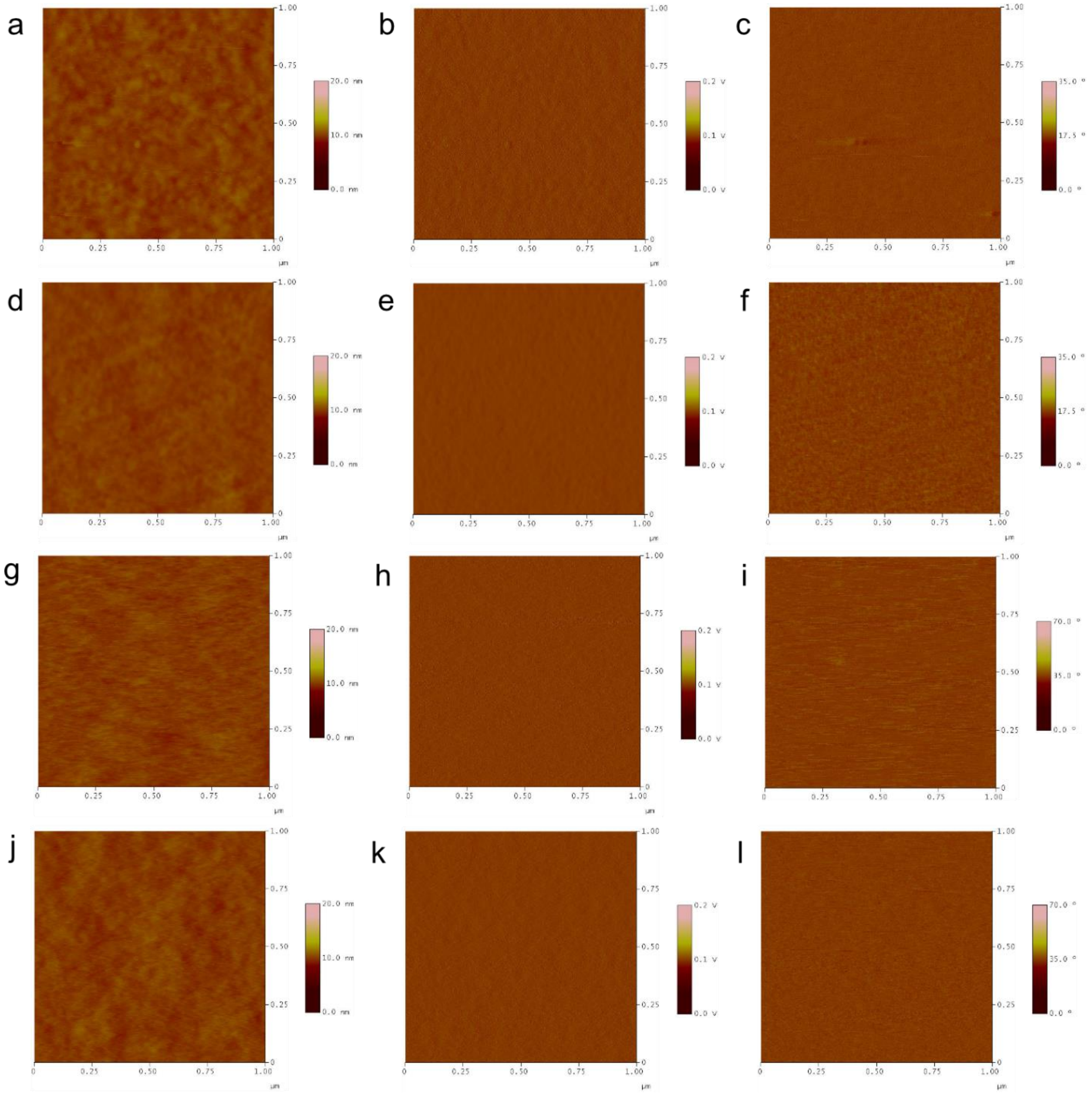

Figure S5: Atomic force microscopy (AFM) images of films of C4 (a: height, b: amplitude, c: phase), C6 (d: height, e: amplitude, f: phase), C8 (g: height, h: amplitude, i: phase) and EH (j: height, k: amplitude, l: phase) revealing smooth surfaces (roughness: C4 0.32 nm, C6 0.20 nm, C8 0.28 nm, EH 0.28 nm).

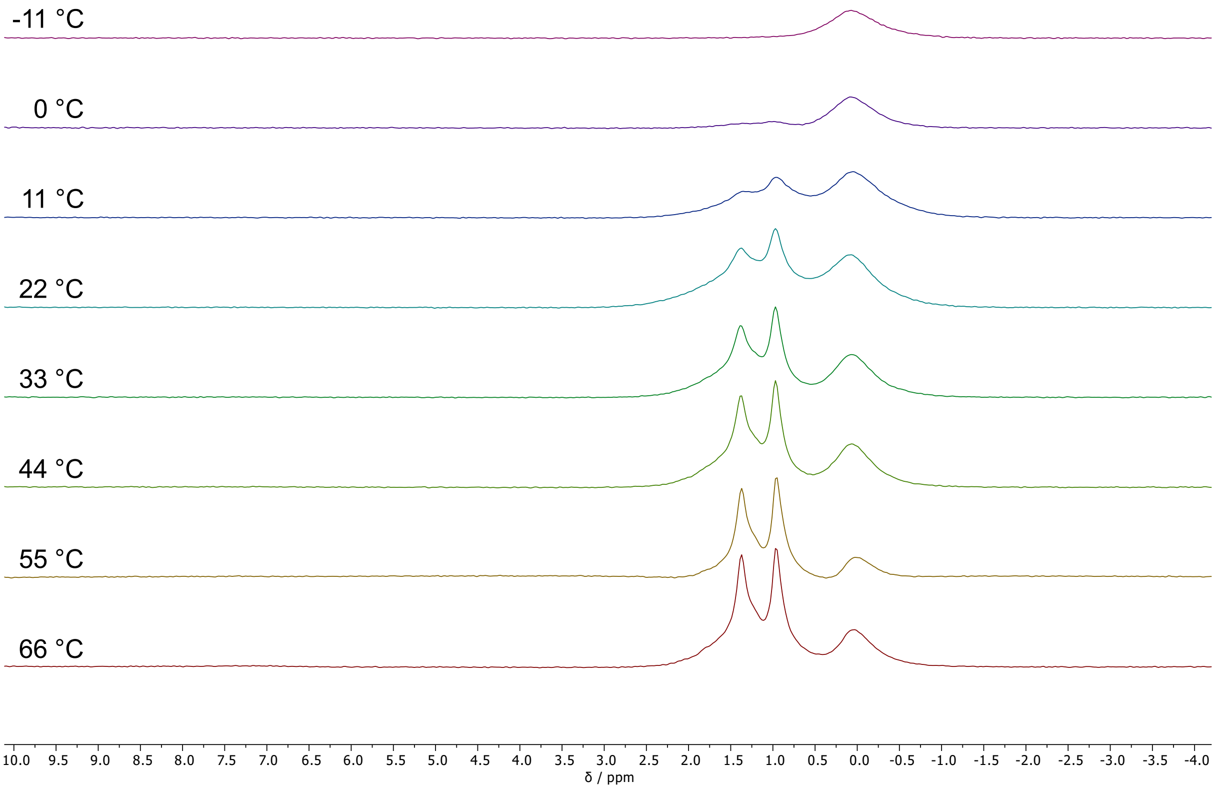

Figure S6: Solid state ^{1}H-NMR of EH with varying temperature, below, within and above T_g at 0.27 °C and T_{iso} at 43.8 °C.

To study the morphology of the nematic EH compound, we perform solid-state ^{1}H measurements at varying temperatures. At -11 °C, below the T_g, we observe a broad signal at 0.04 ppm, which we attribute to frozen alkyl chains. Signals in the aromatic region are not visible under the given measurement conditions. Increasing the temperature to the T_g and beyond, allows the additional formation of signals between 1.0 and 1.5 ppm. This is attributed to alkyl chains, which become mobile above T_g. Above T_{iso} no further significant changes can be observed in the NMR spectrum, but the existing signals sharpen since additional changes would be only expected for the aromatic region. These observations are in accordance with the previously determined phase transition temperatures as measured in DSC and observed in POM.

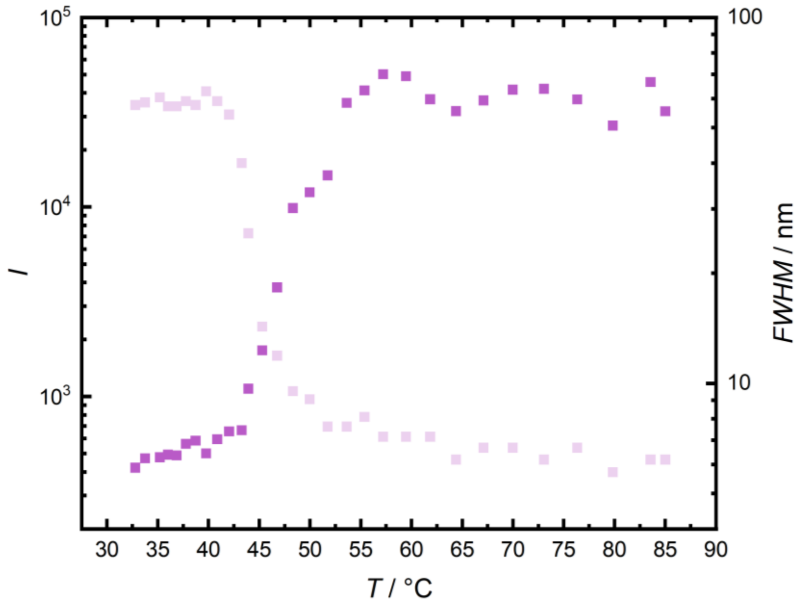

Figure S7: Intensity *I* (dark squares, left axis) and *FWHM* (light squares, right axis) of a film prepared from the EH oligofluorene versus the temperature *T* of the film. The power density of the laser is at E_P = 458 µJ cm^{-2}. At T_{iso} a significant increase for *I* and reduction of *FWHM* is observed, which we attribute to the presence of ASE beyond that temperature.

Synthesis

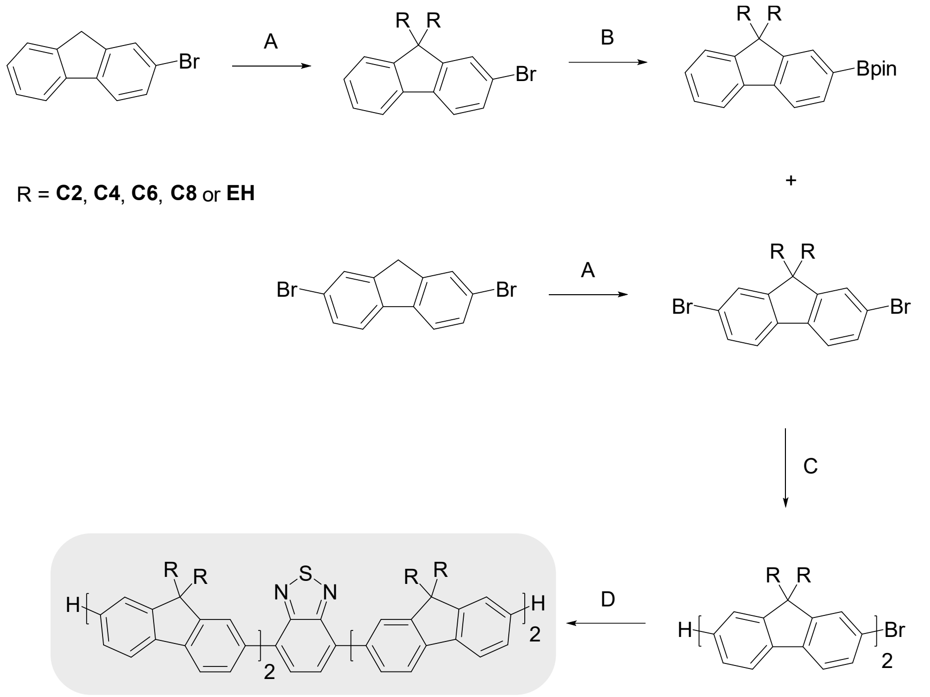

Scheme S1: Overview of the synthesis towards BT-cored pentafluorenes with varying alkyl chains.

General procedure A:[2]

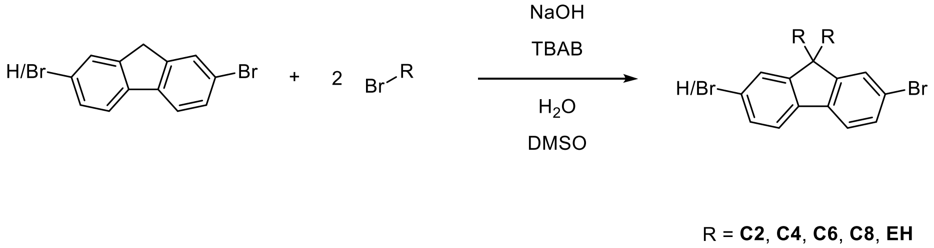

2-Bromo-9H-fluorene or 2,7-dibromo-9H-fluorene (1 eq.) and tetrabutylammonium bromide (0.5 eq.) are dissolved in degassed dimethyl sulfoxide. A degassed saturated aqueous solution of sodium hydroxide (20 eq.) is slowly added under stirring at room temperature. Subsequently, the respective brominated alkane (6 eq.) is added. After stirring overnight, ethyl acetate is added, and the organic phase is washed with aqueous ammonium chloride solution and water. The organic phase is dried over sodium sulfate and filtered off. After removing the solvent and the remaining brominated alkane under *vacuo*, the desired product is obtained as solid or oil.

General procedure B:[2]

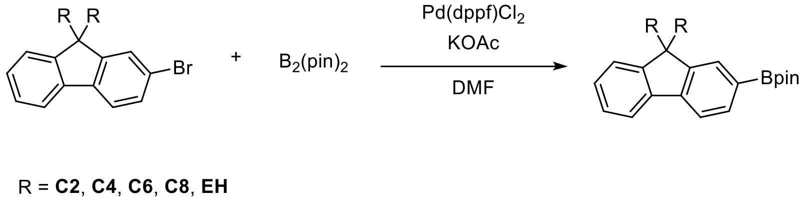

2-Bromo-9,9-dialkyl-9H-fluorene (1 eq.), bis(pinacolato)diboron (1.2 eq or 1.5 eq.), potassium acetate (2.5 eq) and [1,1′-bis(diphenylphosphino)ferrocen]dichlorpalladium(II) (5 mol%) are injected into a dried Schlenk flask. Degassed dimethylformamide is added and the mixture is stirred at 95 °C for 18 h. After cooling down, the reaction is quenched with half concentrated aqueous sodium chloride solution and extracted with dichloromethane. The combined organic fractions are dried over sodium sulfate, filtered and dried under *vacuo*. The crude residue is purified *via* column chromatography (SiO_2, petroleum ether/ethyl acetate 100:0 → 99:1 or 100:0 → 95:5) to obtain the desired borylated fluorenes as solid or oil.

General procedure C:

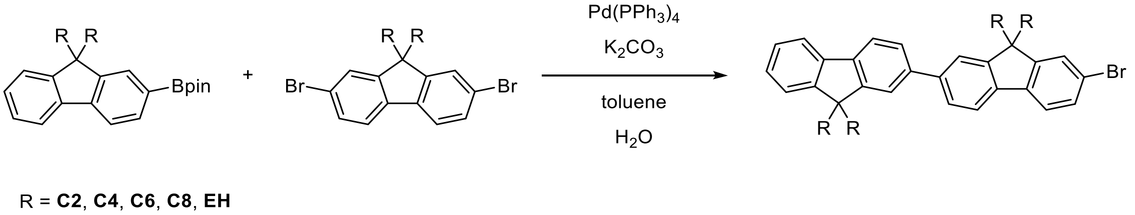

2-(9,9-dialkyl-9H-fluoren-2-yl)-4,4,5,5-tetramethyl-[1,3,2]dioxaborolane (1 eq.), 2,7-dibromo-9,9-dialkyl-9H-fluorene (2.5 eq. or 3 eq.), tetrakis(triphenylphosphine)palladium(0) (3 or 5 mol% Pd) and potassium carbonate (2 eq.) are filled into a Schlenk flask. A degassed solvent mixture of water and toluene (1:5) is added and the mixture is heated to 100 °C. After stirring for 20 h, the emulsion is cooled to rt, quenched with aqueous ammonium chloride solution and extracted with dichloromethane. The combined organic phases are dried over sodium sulfate, filtered and the obtained solution is reduced under *vacuo*. The crude residue is purified *via* column chromatography (SiO_2, petroleum ether/dichloromethane 10:0 or 9:1) to obtain the desired bifluorenes as colorless solid or oil. In some cases, the obtained product is recrystallized from acetone/toluene to improve purity.

General procedure D:[3]

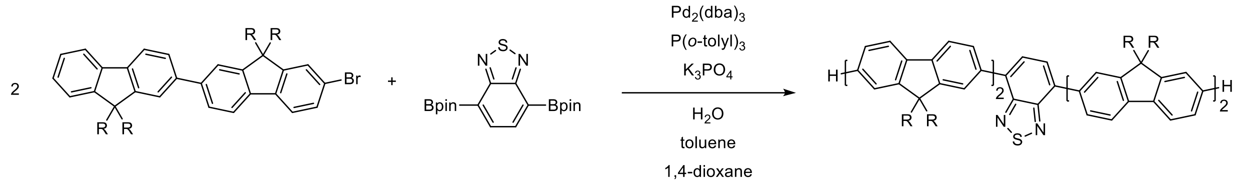

R = **C2**, **C4**, **C6**, **C8**, **EH**

7-Bromo-9,9,9’,9’-tetraalkyl-9H,9H’-2,2’-bifluorene (2 eq.), 4,7-bis-pinacolato-diborane-2,1,3-benzothiadiazole (0.95 or 1 eq.), tris(dibenzylideneacetone)dipalladium(0) (3 mol% Pd), tris(o-tolyl)phosphine (12 mol%) and potassium phosphate (2 eq.) are injected into a Schlenk flask. A degassed solvent mixture of water, toluene and 1,4-dioxane (1/1.8/5.5) is added. After stirring the mixture at 80 °C for 18 h, it is cooled to rt and quenched with aqueous ammonium chloride solution. The organics are extracted with dichloromethane. The combined organic phases are dried over sodium sulfate and filtered to remove remaining solids. The remaining solvents are evaporated under *vacuo* and the crude residue is purified *via* column chromatography (SiO_2, petroleum ether/dichloromethane 9:1, 8:2 or 7:3) to afford the desired pentamers as orange crystalline or amorphous solids.

Synthesis of 2,7-dibromo-9,9-diethyl-9H-fluorene

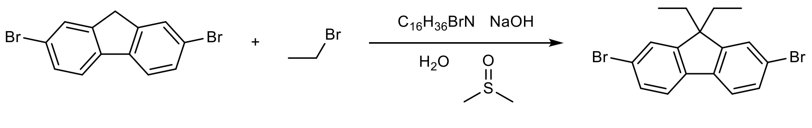

This reaction is performed according to general procedure A using 2,7-dibromo-9H-fluorene (10.0 g, 30.9 mmol, 1 eq.), bromoethane (20.2 g, 185 mmol, 6 eq.), tetrabutylammonium bromide (4.97 g, 15.4 mmol, 0.5 eq.), sodium hydroxide (24.7 g, 617 mmol, 20 eq.) in water (20 ml) and dimethyl sulfoxide (100 ml). The desired product is obtained as greenish solid (11.6 g, 30.4 mmol, 99%).

^{1}H-NMR (400 MHz, $CDCl_3$): δ [ppm] = 7.53 (dd, J = 8.0, 0.7 Hz, 2H), 7.47 (d, J = 1.8 Hz, 1H), 7.45 – 7.43 (m, 2H), 1.99 (q, J = 7.4 Hz, 4H), 0.32 (t, J = 7.4 Hz, 6H).

^{13}C-NMR (100 MHz, $CDCl_3$): δ [ppm] = 151.90, 139.63, 130.37, 126.41, 121.63, 121.24, 56.89, 32.76, 8.55.

HR-MS (APCI pos): [m/z] for $C_{17}H_{16}Br_2$ = 379.96012 (found), 379.95983 (calculated).

Analytical data is in accordance with literature.[4]

Synthesis of 2-bromo-9,9-diethyl-9H-fluorene

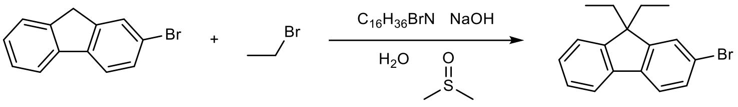

This reaction is performed according to general procedure A using 2-bromo-9H-fluorene (10.0 g, 40.8 mmol, 1 eq.), bromoethane (26.7 g, 245 mmol, 6 eq.), tetrabutylammonium bromide (6.58 g, 20.4 mmol, 0.5 eq.), sodium hydroxide (32.6 g, 816 mmol, 20 eq.) in water (30 ml) and dimethyl sulfoxide (110 ml). The desired product is obtained as orange oil (12.1 g, 40.2 mmol, 99%).

^{1}H-NMR (400 MHz, $CDCl_3$): δ [ppm] = 7.70 – 7.65 (m, 1H), 7.58 – 7.55 (m, 1H), 7.47 – 7.44 (m, 2H), 7.36 – 7.30 (m, 3H), 2.01 (qd, J = 7.4, 2.6 Hz, 4H), 0.32 (t, J = 7.4 Hz, 6H).

^{13}C-NMR (100 MHz, $CDCl_3$): δ [ppm] = 152.30, 149.61, 140.69, 140.58, 130.09, 127.61, 127.15, 126.36, 123.06, 121.12, 119.86, 56.55, 32.81, 8.57. (One aryl signal is not visible due to overlap)

HR-MS (APCI pos): [m/z] for $C_{17}H_{17}Br$ = 302.04895 (found), 302.04932 (calculated).

Analytical data is in accordance with literature.[5]

Synthesis of 2-(9,9-diethyl-9H-fluoren-2-yl)-4,4,5,5-tetramethyl-1,3,2-dioxaborolane

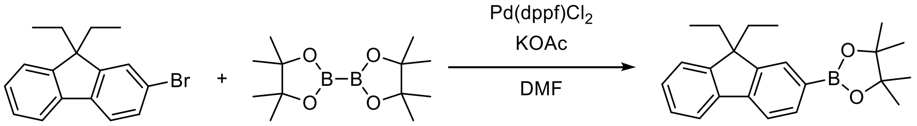

This reaction is performed according to general procedure B using 2-bromo-9,9-diethyl-9H-fluorene (5.00 g, 16.6 mmol, 1 eq.), bis(pinacolato)diboron (6.32 g, 24.9 mmol, 1.5 eq.), [1,1′-bis(diphenylphosphino)ferrocen]dichlorpalladium(II) (678 mg, 830 µmol, 5 mol%), potassium acetate (4.07 g, 41.5 mmol, 2.5 eq.) in dimethylformamide (50 ml). The desired product is obtained as ivory solid (5.56 g, 16.0 mmol, 96%). Residual reagents are removed during the purification of the next step.

^{1}H-NMR (400 MHz, $CDCl_3$): δ [ppm] = 7.81 (dd, J = 7.6, 1.1 Hz, 1H), 7.77 – 7.69 (m, 3H), 7.35 – 7.31 (m, 3H), 2.16 – 1.96 (m, 4H), 1.39 (s, 12H), 0.29 (t, J = 7.4 Hz, 6H).

^{13}C-NMR (100 MHz, $CDCl_3$): δ [ppm] = 150.62, 149.13, 144.70, 141.48, 133.88, 129.07, 127.64, 126.89, 123.08, 120.21, 119.07, 83.84, 56.27, 32.79, 25.09, 8.64. (One aryl and one alkyl signal are not visible due to overlap)

HR-MS (APCI pos): [m/z] for $C_{23}H_{29}BO_2$ = 348.22517 (found), 348.22606 (calculated).

Analytical data is in accordance with literature.[6]

Synthesis of 7-bromo-9,9,9',9'-tetraethyl-9H,9H'-2,2'-bifluorene

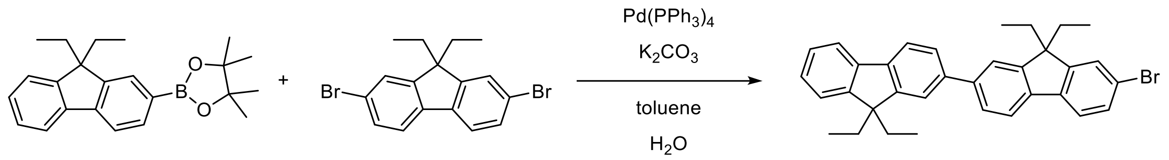

This reaction is performed according to general procedure C using 2-(9,9-diethyl-9H-fluoren-2-yl)-4,4,5,5-tetramethyl-1,3,2-dioxaborolane (2.00 g, 5.74 mmol, 1 eq.), 2,7-dibromo-9,9-diethyl-9H-fluorene (6.55 g, 17.2 mmol, 3 eq.), tetrakis(triphenylphosphine)palladium(0) (199 mg, 172 µmol, 3 mol% Pd) and potassium carbonate (1.59 g, 11.5 mmol, 2 eq.) in a solvent mixture of water (6.5 ml) and toluene (40 ml). After column chromatography (SiO_2, petroleum ether/dichloromethane 10:0 → 9:1) and recrystallisation from a mixture of acetone and toluene, the desired product is obtained as colorless solid (1.33 g, 2.55 mmol, 44%).

^{1}H-NMR (400 MHz, $CDCl_3$): δ [ppm] = 7.80 – 7.73 (m, 3H), 7.65 (ddd, J = 7.9, 5.6, 1.7 Hz, 2H), 7.62 – 7.56 (m, 3H), 7.51 – 7.46 (m, 2H), 7.39 – 7.30 (m, 3H), 2.22 – 2.01 (m, 8H), 0.40 (td, J = 7.4, 1.7 Hz, 12H).

^{13}C-NMR (100 MHz, $CDCl_3$): δ [ppm] = 152.58, 150.80, 150.39, 150.29, 141.31, 141.25, 141.01, 140.46, 140.44, 139.73, 130.19, 127.21, 127.03, 126.50, 126.41, 126.27, 123.09, 121.58, 121.57, 121.18, 121.11, 120.12, 120.03, 119.87, 56.74, 56.38, 32.96, 32.92, 8.76, 8.73.

HR-MS (MALDI pos): [m/z] for $C_{34}H_{33}Br$ = 520.17602 (found), 520.17656 (calculated).

Analytical data is in accordance with literature.[6]

Synthesis of 4,7-bis(9,9,9',9'-tetraethyl-9H,9'H-[2,2'-bifluoren]-7-yl)benzo[c][1,2,5]thiadiazole (C2)

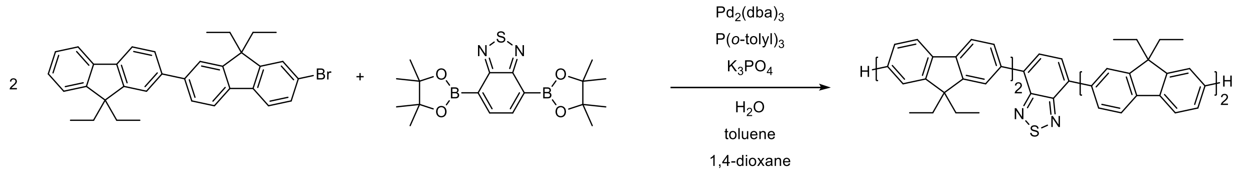

This reaction is performed according to general procedure D using 7-bromo-9,9,9',9'-tetraethyl-9H,9H'-2,2'-bifluorene (700 mg, 1.34 mmol, 2 eq.), 4,7-bis-pinacolato-diborane-

2,1,3-benzothiadiazole (260 mg, 671 µmol, 1 eq.), tris(dibenzylideneacetone)dipalladium(0) (9.22 mg, 10.1 µmol, 3 mol% Pd), tris(o-tolyl)phosphine (24.5 mg, 80.5 µmol, 12 mol%) and potassium phosphate (285 mg, 1.34 mmol, 2 eq.) in 40 ml of the solvent mixture. After column chromatography (SiO_2, petroleum ether/dichloromethane 8:2 → 7:3), the desired product is obtained as a bright yellow powder (376 mg, 369 µmol, 55%).

^{1}H-NMR (400 MHz, $CDCl_3$): δ [ppm] = 8.07 (dd, J = 7.8, 1.6 Hz, 2H), 8.01 (d, J = 1.5 Hz, 2H), 7.96 – 7.90 (m, 4H), 7.87 (d, J = 7.9 Hz, 2H), 7.81 (d, J = 7.8 Hz, 2H), 7.78 – 7.75 (m, 2H), 7.71 (dd, J = 8.0, 1.6 Hz, 3H), 7.68 – 7.63 (m, 5H), 7.42 – 7.30 (m, 6H), 2.28 – 2.17 (m, 8H), 2.17 – 2.05 (m, 8H), 0.54 (t, J = 7.3 Hz, 12H), 0.42 (t, J = 7.3 Hz, 12H).

^{13}C-NMR (100 MHz, $CDCl_3$): δ [ppm] = 154.52, 151.43, 150.79, 150.78, 150.32, 141.62, 141.38, 141.04, 140.94, 140.66, 140.39, 136.39, 133.71, 128.49, 128.10, 127.17, 127.03, 126.46, 126.30, 124.10, 123.09, 121.70, 121.62, 120.34, 120.04, 119.93, 119.86, 56.58, 56.39, 32.99, 32.97, 9.00, 8.78.

HR-MS (MALDI pos): [m/z] for $C_{74}H_{68}N_2S$ = 1016.50919 (found), 1016.51032 (calculated).

Synthesis of 2,7-dibromo-9,9-dibutyl-9H-fluorene

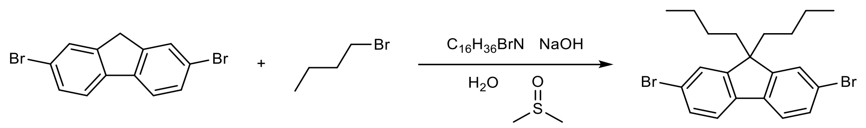

This reaction is performed according to general procedure A using 2,7-dibromo-9H-fluorene (10.0 g, 30.9 mmol, 1 eq.), bromobutane (20.2 g, 185 mmol, 6 eq.), tetrabutylammonium bromide (4.97 g, 15.4 mmol, 0.5 eq.), sodium hydroxide (24.7 g, 617 mmol, 20 eq.) in water (20 ml) and dimethyl sulfoxide (100 ml). The desired product is obtained as greenish solid (11.6 g, 30.4 mmol, 99%).

^{1}H-NMR (400 MHz, $CDCl_3$): δ [ppm] = 7.52 (dd, J = 7.6, 0.9 Hz, 2H), 7.46 (d, J = 1.8 Hz, 1H), 7.45 – 7.44 (m, 2H), 1.96 – 1.89 (m, 4H), 1.09 (h, J = 7.4 Hz, 4H), 0.69 (t, J = 7.3 Hz, 6H), 0.63 – 0.51 (m, 4H).
^{13}C-NMR (100 MHz, $CDCl_3$): δ [ppm] = 152.69, 139.22, 130.31, 126.32, 121.62, 121.28, 55.75, 40.17, 25.96, 23.09, 13.92.

HR-MS (APCI pos): [m/z] for $C_{21}H_{24}Br_2$ = 436.02060 (found), 436.02243 (calculated).

Analytical data is in accordance with literature.[7]

Synthesis of 2-bromo-9,9-dibutyl-9H-fluorene

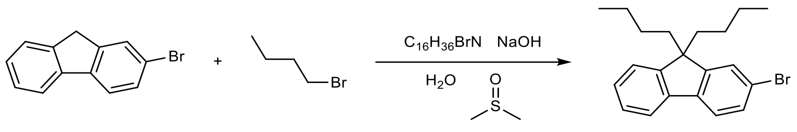

This reaction is performed according to general procedure A using 2-bromo-9H-fluorene (10.0 g, 40.8 mmol, 1 eq.), bromobutane (33.5 g, 245 mmol, 6 eq.), tetrabutylammonium bromide (6.58 g, 20.4 mmol, 0.5 eq.), sodium hydroxide (32.6 g, 816 mmol, 20 eq.) in water (30 ml) and dimethyl sulfoxide (100 ml). The desired product is obtained as yellow oil (14.4 g, 40.3 mmol, 99%).

^{1}H-NMR (400 MHz, $CDCl_3$): δ [ppm] = 7.70 – 7.65 (m, 1H), 7.56 (dd, J = 7.8, 0.7 Hz, 1H), 7.50 – 7.42 (m, 2H), 7.37 – 7.30 (m, 3H), 2.04 – 1.87 (m, 4H), 1.17 – 1.01 (m, 4H), 0.69 (t, J = 7.3 Hz, 6H), 0.66 – 0.50 (m, 4H).

^{13}C-NMR (100 MHz, $CDCl_3$): δ [ppm] = 153.09, 150.44, 140.28, 140.18, 130.02, 127.60, 127.06, 126.27, 123.02, 121.16, 121.11, 119.88, 55.43, 40.25, 26.01, 23.15, 13.93.

HR-MS (APCI pos): [m/z] for $C_{21}H_{25}Br$ = 358.11177 (found), 358.11192 (calculated).

Analytical data is in accordance with literature.[8]

Synthesis of 2-(9,9-dibutyl-9H-fluoren-2-yl)-4,4,5,5-tetramethyl-1,3,2-dioxaborolane

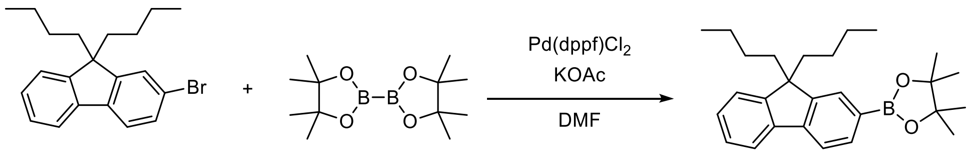

This reaction is performed according to general procedure B using 2-bromo-9,9-dibutyl-9H-fluorene (5.97 g, 16.7 mmol, 1 eq.), bis(pinacolato)diboron (6.41 g, 25.2 mmol, 1.5 eq.), [1,1′-bis(diphenylphosphino)ferrocen]dichlorpalladium(II) (611 mg, 835 µmol, 5 mol%), potassium acetate (4.10 g, 41.8 mmol, 2.5 eq.) in dimethylformamide (60 ml). The desired product is obtained as yellow oil (3.86 g, 9.54 mmol, 57%).

^{1}H-NMR (400 MHz, $CDCl_3$): δ [ppm] = 7.81 (dd, J = 7.5, 1.0 Hz, 1H), 7.76 – 7.69 (m, 3H), 7.37 – 7.30 (m, 3H), 2.06 – 1.91 (m, 4H), 1.11 – 1.01 (m, 4H), 0.65 (t, J = 7.4 Hz, 6H), 0.63 – 0.47 (m, 4H).

^{13}C-NMR (100 MHz, CDCl$_3$): δ [ppm] = 151.45, 150.00, 144.27, 141.08, 133.85, 128.96, 127.62, 126.81, 123.09, 120.23, 119.10, 83.85, 55.15, 40.24, 26.04, 25.10, 23.20, 13.96. (One aryl signal missing)

HR-MS (APCI pos): [m/z] for $C_{27}H_{37}BO_2$ = 404.28726 (found), 404.28866 (calculated).

Synthesis of 7-bromo-9,9,9',9'-tetrabutyl-9H,9H'-2,2'-bifluorene

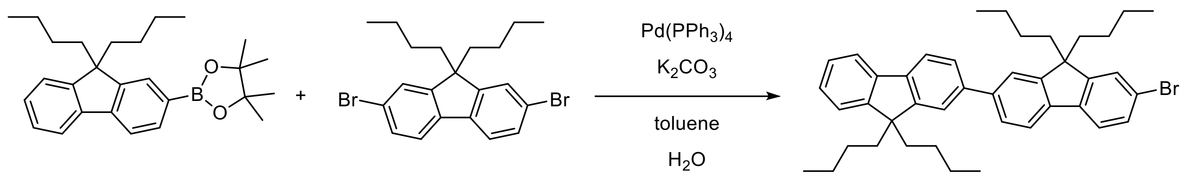

This reaction is performed according to general procedure C using 2-(9,9-dibutyl-9H-fluoren-2-yl)-4,4,5,5-tetramethyl-1,3,2-dioxaborolane (2.01 g, 4.97 mmol, 1 eq.), 2,7-dibromo-9,9-dibutyl-9H-fluorene (6.50 g, 14.9 mmol, 3 eq.), tetrakis(triphenylphosphine)palladium(0) (172 mg, 149 µmol, 3 mol% Pd) and potassium carbonate (1.37 g, 9.94 mmol, 2 eq.) in a solvent mixture of water (6.5 ml) and toluene (40 ml). After column chromatography (SiO_2, petroleum ether/dichloromethane 10:0 → 95:5) and recrystallisation from acetone, the desired product is obtained as a colorless solid (1.33 g, 2.55 mmol, 44%).

^{1}H-NMR (400 MHz, CDCl$_3$): δ [ppm] = 7.79 (dd, J = 7.9, 0.6 Hz, 1H), 7.76 – 7.72 (m, 2H), 7.65 (ddd, J = 7.7, 5.7, 1.7 Hz, 2H), 7.62 – 7.58 (m, 3H), 7.50 – 7.46 (m, 2H), 7.39 – 7.30 (m, 3H), 2.11 – 1.93 (m, 8H), 1.12 (qd, J = 7.4, 5.6 Hz, 8H), 0.77 – 0.63 (m, 20H).

^{13}C-NMR (100 MHz, CDCl$_3$): δ [ppm] = 153.39, 151.65, 151.25, 151.14, 141.10, 140.87, 140.64, 140.32, 140.00, 139.35, 130.15, 127.21, 126.96, 126.38, 126.35, 126.14, 123.09, 121.52, 121.51, 121.22, 121.12, 120.15, 120.06, 119.90, 55.60, 55.25, 40.34, 40.28, 26.17, 26.11, 23.22, 23.17, 13.96, 13.95.

HR-MS (MALDI pos): [m/z] for $C_{42}H_{49}Br$ = 632.30130 (found), 632.30176 (calculated).

Synthesis of 4,7-bis(9,9,9',9'-tetrabutyl-9H,9'H-[2,2'-bifluoren]-7-yl)benzo[c][1,2,5]thiadiazole (C4)

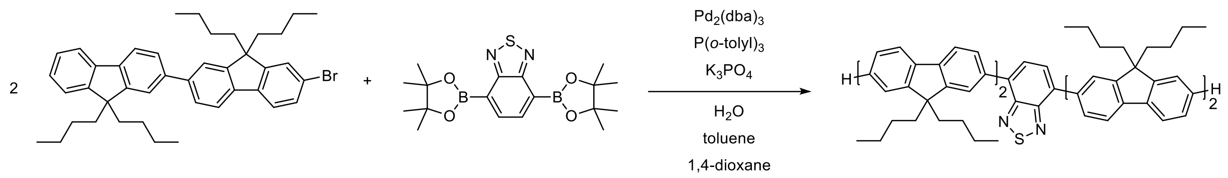

This reaction is performed according to general procedure D using 7-bromo-9,9,9',9'-tetrabutyl-9H,9H'-2,2'-bifluorene (700 mg, 1.10 mmol, 2 eq.), 4,7-bis-pinacolato-diborane-2,1,3-benzothiadiazole (214 mg, 552 µmol, 1 eq.), tris(dibenzylideneacetone)dipalladium(0) (7.59 mg, 8.28 µmol, 3 mol% Pd), tris(o-tolyl)phosphine (20.2 mg, 66.3 µmol, 12 mol%) and potassium phosphate (234 mg, 1.10 mmol, 2 eq.) in 40 ml of the solvent mixture. After column chromatography (SiO_2, petroleum ether/dichloromethane 8:2), the desired product is obtained as an orange solid (604 mg, 486 µmol, 88%).

^{1}H-NMR (400 MHz, $CDCl_3$): δ [ppm] = 8.09 (dd, J = 7.8, 1.6 Hz, 1H), 8.01 (d, J = 1.5 Hz, 1H), 7.95 – 7.91 (m, 2H), 7.88 (d, J = 7.7 Hz, 1H), 7.81 (d, J = 7.8 Hz, 1H), 7.78 – 7.74 (m, 1H), 7.73 – 7.66 (m, 4H), 7.41 – 7.30 (m, 3H), 2.23 – 1.98 (m, 8H), 1.24 – 1.07 (m, 8H), 0.93 – 0.82 (m, 4H), 0.74 (dt, J = 17.1, 7.3 Hz, 16H).

^{13}C-NMR (100 MHz, $CDCl_3$): δ [ppm] = 154.54, 152.26, 151.64, 151.60, 151.16, 141.20, 140.94, 140.90, 140.57, 140.52, 140.02, 136.36, 133.76, 128.45, 128.09, 127.18, 126.96, 126.34, 126.18, 124.10, 123.10, 121.64, 121.55, 120.37, 120.07, 119.95, 119.89, 55.47, 55.26, 40.38, 40.30, 26.35, 26.19, 23.30, 23.25, 14.02, 13.98.

HR-MS (MALDI pos): [m/z] for $C_{90}H_{100}N_2S$ = 1240.75894 (found), 1240.76072 (calculated).

Synthesis of 2-(9,9-dihexyl-9H-fluoren-2-yl)-4,4,5,5-tetramethyl-1,3,2-dioxaborolane

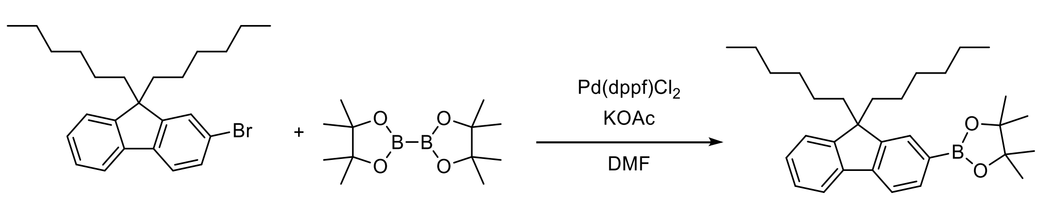

This reaction is performed according to general procedure B using 2-bromo-9,9-dihexyl-9H-fluorene (4.04 g, 9.77 mmol, 1 eq.), bis(pinacolato)diboron (3.72 g, 14.7 mmol, 1.5 eq.), [1,1′-bis(diphenylphosphino)ferrocen]dichlorpalladium(II) (357 mg, 835 µmol, 5 mol%), potassium acetate (2.40 g, 24.4 mmol, 2.5 eq.) in dimethylformamide (40 ml). The desired product is obtained as yellow oil (3.28 g, 7.11 mmol, 73%).

^{1}H-NMR (400 MHz, $CDCl_3$): δ [ppm] = 7.80 (dd, J = 7.6, 1.0 Hz, 1H), 7.75 – 7.67 (m, 3H), 7.36 – 7.29 (m, 3H), 2.06 – 1.89 (m, 4H), 1.39 (s, 12H), 1.13 – 0.97 (m, 12H), 0.75 (t, J = 7.1 Hz, 6H), 0.58 (q, J = 5.6 Hz, 4H).

^{13}C-NMR (100 MHz, $CDCl_3$): δ [ppm] = 151.44, 150.01, 144.26, 141.06, 133.83, 128.97, 127.61, 126.79, 123.06, 120.22, 119.09, 83.82, 55.21, 40.38, 31.61, 29.81, 25.08, 23.78, 22.71, 14.14. (one aryl signal is not visible due to overlap)

HR-MS (APCI pos): [m/z] for $C_{31}H_{45}BO_2$ = 460.35032 (found), 460.35126 (calculated).

Analytical data is in accordance with literature.[9]

Synthesis of 7-bromo-9,9,9',9'-tetrahexyl-9H,9H'-2,2'-bifluorene

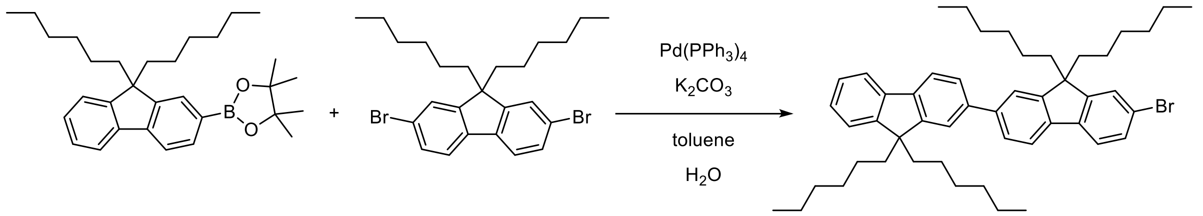

This reaction is performed according to general procedure C using 2-(9,9-dihexyl-9H-fluoren-2-yl)-4,4,5,5-tetramethyl-1,3,2-dioxaborolane (1.55 g, 3.37 mmol, 1 eq.), 2,7-dibromo-9,9-dihexyl-9H-fluorene (4.97 g, 10.1 mmol, 3 eq.), tetrakis(triphenylphosphine)palladium(0) (117 mg, 101 µmol, 3 mol% Pd) and potassium carbonate (930 mg, 6.73 mmol, 2 eq.) in a solvent mixture of water (5 ml) and toluene (30 ml). After column chromatography (SiO_2, petroleum ether) and recrystallisation from acetone, the desired product is obtained as a colorless solid (747 mg, 1.00 mmol, 30%).

^{1}H-NMR (400 MHz, $CDCl_3$): δ [ppm] = 7.81 – 7.70 (m, 3H), 7.63 (ddd, J = 7.7, 5.8, 1.6 Hz, 2H), 7.60 – 7.57 (m, 3H), 7.49 – 7.45 (m, 2H), 7.38 – 7.28 (m, 3H), 2.10 – 1.91 (m, 8H), 1.18 – 1.04 (m, 26H), 0.80 – 0.66 (m, 19H).

^{13}C-NMR (100 MHz, $CDCl_3$): δ [ppm] = 153.39, 151.64, 151.23, 151.13, 141.19, 140.86, 140.60, 140.41, 139.99, 139.31, 130.12, 127.19, 126.94, 126.41, 126.34, 126.18, 123.07, 121.57, 121.55, 121.20, 121.09, 120.12, 120.04, 119.88, 55.66, 55.31, 40.49, 40.41, 31.60, 31.59, 29.82, 29.75, 23.90, 23.87, 22.70, 22.69, 14.14, 14.14.

HR-MS (MALDI pos): [m/z] for $C_{50}H_{65}Br$ = 744.42692 (found), 744.42697 (calculated).

Analytical data is in accordance with literature.[10]

Synthesis of 4,7-bis(9,9,9',9'-tetrahexyl-9H,9'H-[2,2'-bifluoren]-7-yl)benzo[c][1,2,5]thiadiazole (C6)

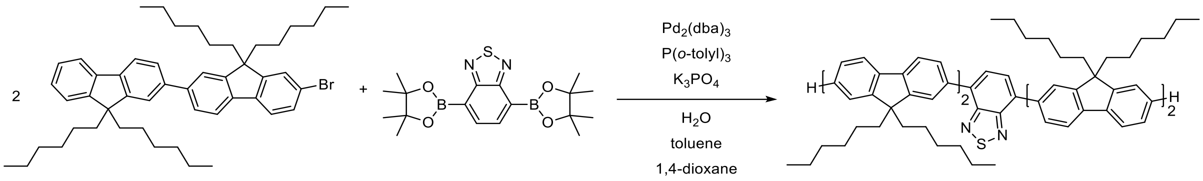

This reaction is performed according to general procedure D using 7-bromo-9,9,9’,9’-tetrahexyl-9H,9H’-2,2’-bifluorene (500 mg, 670 µmol, 2 eq.), 4,7-bis-pinacolato-diborane-2,1,3-benzothiadiazole (130 mg, 335 µmol, 1 eq.), tris(dibenzylideneacetone)dipalladium(0) (4.60 mg, 5.03 µmol, 3 mol% Pd), tris(o-tolyl)phosphine (12.2 mg, 40.2 µmol, 12 mol%) and potassium phosphate (142 mg, 670 µmol, 2 eq.) in 20 ml of the solvent mixture. After column chromatography (SiO_2, petroleum ether/dichloromethane 9:1 → 8:2), the desired product is obtained as an orange solid (604 mg, 486 µmol, 88%).

^{1}H-NMR (400 MHz, $CDCl_3$): δ [ppm] = 8.07 (dd, J = 7.9, 1.6 Hz, 2H), 8.01 (d, J = 1.5 Hz, 2H), 7.95 – 7.91 (m, 4H), 7.87 (d, J = 7.8 Hz, 2H), 7.80 (d, J = 7.8 Hz, 2H), 7.77 – 7.74 (m, 2H), 7.71 – 7.63 (m, 8H), 7.41 – 7.29 (m, 6H), 2.20 – 2.09 (m, 8H), 2.09 – 1.96 (m, 8H), 1.23 – 1.01 (m, 48H), 0.94 – 0.85 (m, 8H), 0.84 – 0.66 (m, 32H).

^{13}C-NMR (100 MHz, $CDCl_3$): δ [ppm] = 154.55, 152.27, 151.63, 151.58, 151.17, 141.19, 140.99, 140.94, 140.61, 140.53, 139.97, 136.33, 133.77, 128.40, 128.07, 127.16, 126.94, 126.37, 126.21, 124.16, 123.08, 121.70, 121.61, 120.35, 120.04, 119.94, 119.88, 55.52, 55.33, 40.54, 40.46, 31.64, 31.63, 29.89, 29.85, 24.09, 23.93, 22.74, 22.72, 14.19, 14.16.

HR-MS (MALDI pos): [m/z] for $C_{106}H_{132}N_2S$ = 1466.01447 (found), 1466.01448 (calculated).

Synthesis of 7-bromo-9,9,9’,9’-tetraoctyl-9H,9H’-2,2’-bifluorene

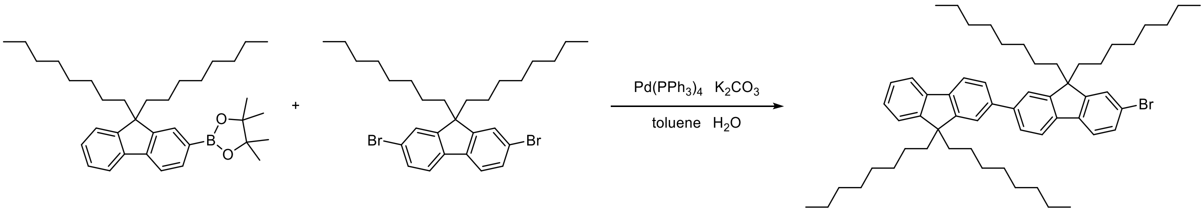

This reaction is performed according to general procedure C using 2-(9,9-dioctyl-9H-fluoren-2-yl)-4,4,5,5-tetramethyl-1,3,2-dioxaborolane (628 mg, 1.22 mmol, 1 eq.), 2,7-dibromo-9,9-dioctyl-9H-fluorene (1.00 g, 1.82 mmol, 1.5 eq.), tetrakis(triphenylphosphine)palladium(0) (70.2 mg, 60.8 µmol, 3 mol% Pd) and potassium carbonate (336 mg, 2.43 mmol, 2 eq.) in a solvent mixture of water (2 ml) and toluene (10 ml). After column chromatography (SiO_2, petroleum ether), the desired product is obtained as a colorless oil (571 mg, 665 µmol, 55%).

^{1}H-NMR (400 MHz, $CDCl_3$): δ [ppm] = 7.76 (dd, J = 14.1, 7.8 Hz, 3H), 7.66 – 7.61 (ddd, J = 7.7, 5.8, 1.6 Hz, 2H), 7.61 – 7.56 (m, 3H), 7.50 – 7.45 (m, 2H), 7.40 – 7.29 (m, 3H), 2.10 – 1.91 (m, 8H), 1.28 – 1.04 (m, 40H), 0.81 (td, J = 7.0, 3.4 Hz, 12H), 0.77 – 0.63 (m, 8H).

^{13}C-NMR (100 MHz, $CDCl_3$): δ [ppm] = 153.40, 151.65, 151.24, 151.14, 141.17, 140.87, 140.60, 140.39, 140.00, 139.32, 130.12, 127.18, 126.94, 126.41, 126.35, 126.18, 123.09, 121.55, 121.53, 121.20, 121.09, 120.11, 120.03, 119.88, 55.67, 55.32, 40.49, 40.41, 31.92, 30.17, 30.09, 29.35, 29.32, 23.95, 23.90, 22.74, 14.21. (Five alkyl signals are not visible due to overlap)

HR-MS (MALDI pos): [m/z] for $C_{58}H_{81}Br$ = 858.55074 (found), 858.55012 (calculated).

Analytical data is in accordance with literature.[11]

Synthesis of 4,7-bis(9,9,9',9'-tetraoctyl-9H,9'H-[2,2'-bifluoren]-7-yl)benzo[c][1,2,5]thiadiazole (C8)

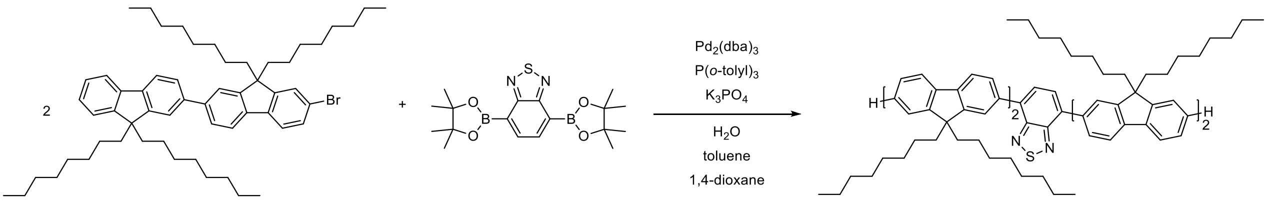

This reaction is performed according to general procedure D using 7-bromo-9,9,9’,9’-tetraoctyl-9H,9H’-2,2’-bifluorene (500 mg, 583 µmol, 2 eq.), 4,7-bis-pinacolato-diborane-2,1,3-benzothiadiazole (113 mg, 291 µmol, 1 eq.), tris(dibenzylideneacetone)dipalladium(0) (4.00 mg, 4.37 µmol, 3 mol% Pd), tris(o-tolyl)phosphine (10.6 mg, 35.0 µmol, 12 mol%) and potassium phosphate (124 mg, 583 µmol, 2 eq.) in 20 ml of the solvent mixture. After column chromatography (SiO_2, petroleum ether/dichloromethane 9:1 → 8:2), the desired product is obtained as an orange solid (339 mg, 201 µmol, 69%).

^{1}H-NMR (400 MHz, CDCl$_3$): δ [ppm] = 8.09 (dd, J = 7.7, 1.6 Hz, 2H), 8.02 (s, 2H), 7.95 – 7.93 (t, J = 3.9 Hz, 4H), 7.88 (d, J = 7.8 Hz, 2H), 7.80 (d, J = 7.8 Hz, 2H), 7.75 (d, J = 7.2 Hz, 2H), 7.73 – 7.64 (m, 8H), 7.39 – 7.31 (m, 6H), 2.21 – 1.99 (m, 16H), 1.35 – 1.05 (m, 80H), 0.98 – 0.64 (m, 40H).

^{13}C-NMR (100 MHz, CDCl$_3$): δ [ppm] = 154.55, 152.27, 151.64, 151.59, 151.17, 141.20, 140.97, 140.94, 140.58, 140.53, 139.97, 136.32, 133.77, 128.41, 128.06, 127.15, 126.94, 126.38, 126.21, 124.14, 123.09, 121.67, 121.58, 120.34, 120.03, 119.94, 119.87, 55.53, 55.33, 40.53, 40.45, 31.96, 31.94, 30.23, 30.19, 29.40, 29.37, 24.13, 23.98, 22.75, 14.22. (Four alkyl signals are not visible due to overlap)

HR-MS (MALDI pos): [m/z] for $C_{122}H_{164}N_2S$ = 1690.26421 (found), 1690.26488 (calculated).

Analytical data is in accordance with literature.[3]

Synthesis of 2,7-dibromo-9,9-bis(2-ethylhexyl)-9H-fluorene

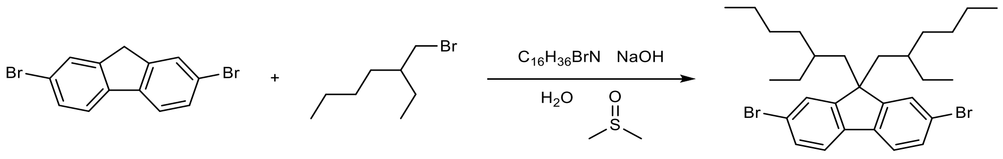

This reaction is performed according to general procedure A using 2,7-dibromo-9H-fluorene (10.0 g, 30.9 mmol, 1 eq.), 3-(bromomethyl)heptane (35.8 g, 185 mmol, 6 eq.), tetrabutylammonium bromide (4.97 g, 15.4 mmol, 0.5 eq.), sodium hydroxide (24.7 g, 617 mmol, 20 eq.) in water (20 ml) and dimethyl sulfoxide (100 ml). The desired product is obtained as yellow oil (16.7 g, 30.5 mmol, 99%).

^{1}H-NMR (400 MHz, CDCl$_3$): δ [ppm] = 7.54 – 7.48 (m, 4H), 7.45 (dd, J = 8.0, 1.8 Hz, 2H), 2.01 – 1.87 (m, 4H), 0.99 – 0.63 (m, 22H), 0.54 (td, J = 7.4, 2.7 Hz, 6H), 0.48 (p, J = 5.7 Hz, 2H).

^{13}C-NMR (100 MHz, CDCl$_3$): δ [ppm] = 152.58, 152.53, 139.33, 139.31, 139.29, 130.26, 130.23, 130.20, 127.61, 127.53, 127.46, 121.19, 121.11, 121.07, 121.03, 55.50, 44.46, 34.82, 33.75, 33.71, 28.18, 28.15, 27.22, 27.19, 22.86, 14.17, 10.47, 10.45. (Multiple Peaks are observed due to the presence of diastereomers)

HR-MS (APCI pos): [m/z] for $C_{29}H_{40}Br_2$ = 548.14625 (found), 548.14763 (calculated).

Analytical data is in accordance with literature.[12]

Synthesis of 2-bromo-9,9-bis(2-ethylhexyl)-9H-fluorene

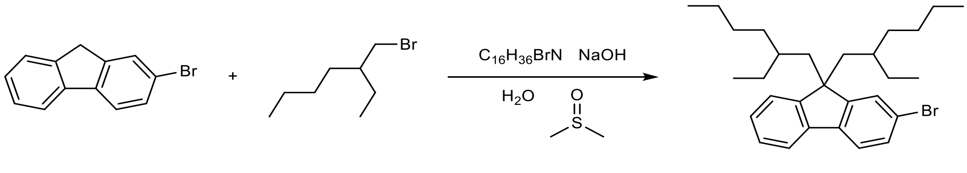

This reaction is performed according to general procedure A using 2-bromo-9H-fluorene (10.0 g, 40.8 mmol, 1 eq.), 3-(bromomethyl)heptane (47.3 g, 245 mmol, 6 eq.), tetrabutylammonium bromide (6.58 g, 20.4 mmol, 0.5 eq.), sodium hydroxide (32.6 g, 816 mmol, 20 eq.) in water (30 ml) and dimethyl sulfoxide (100 ml). The desired product is obtained as yellow oil (18.9 g, 40.2 mmol, 99%).

^{1}H-NMR (400 MHz, $CDCl_3$): δ [ppm] = 7.69 – 7.61 (m, 1H), 7.55 (d, J = 8.0 Hz, 1H), 7.51 (td, J = 4.1, 1.8 Hz, 1H), 7.44 (dd, J = 8.1, 1.8 Hz, 1H), 7.39 – 7.33 (m, 1H), 7.30 (ddd, J = 8.7, 6.9, 1.4 Hz, 2H), 2.04 – 1.88 (m, 4H), 0.99 – 0.64 (m, 22H), 0.58 – 0.44 (m, 8H).

^{13}C-NMR (100 MHz, $CDCl_3$): δ [ppm] = 152.94, 150.21, 150.15, 150.08, 140.43, 140.36, 140.33, 140.30, 129.94, 129.92, 129.90, 127.55, 127.47, 127.38, 127.07, 127.02, 124.26, 124.22, 124.19, 121.04, 120.57, 120.52, 120.47, 119.81, 55.28, 55.26, 55.25, 44.66, 44.63, 44.52, 44.50, 34.73, 33.81, 33.78, 33.76, 33.74, 28.26, 28.24, 28.16, 28.14, 27.20, 27.18, 27.04, 27.02, 22.88, 22.80, 14.19, 14.12, 10.51, 10.49, 10.33, 10.31. (Multiple Peaks are observed due to the presence of diastereomers)

HR-MS (APCI pos): [m/z] for $C_{29}H_{41}Br$ = 468.23990 (found), 468.23916 (calculated).

Analytical data is in accordance with literature.[12]

Synthesis of 2-(9,9-bis(2-ethylhexyl)-9H-fluoren-2-yl)-4,4,5,5-tetramethyl-1,3,2-dioxaborolane

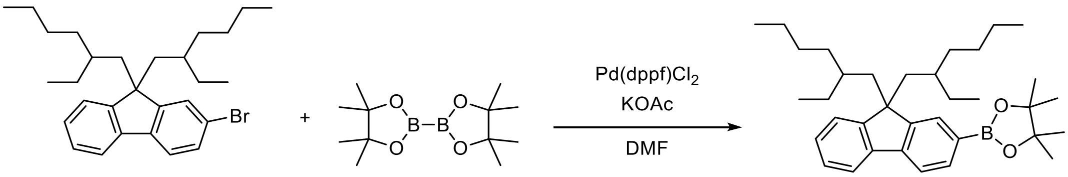

This reaction is performed according to general procedure B using 2-bromo-9,9-bis(2-ethylhexyl)-9H-fluorene (7.50 g, 16.0 mmol, 1 eq.), bis(pinacolato)diboron (4.87 g, 19.2 mmol, 1.2 eq.), [1,1′-bis(diphenylphosphino)ferrocen]dichlorpalladium(II) (575 mg, 799 µmol, 5 mol%), potassium acetate (3.92 g, 39.9 mmol, 2.5 eq.) in dimethylformamide (75 ml). The desired product is obtained as dark yellow oil (7.24 g, 14.0 mmol, 88%).

^{1}H-NMR (400 MHz, $CDCl_3$): δ [ppm] = 7.86 – 7.76 (m, 2H), 7.73 – 7.66 (m, 2H), 7.41 – 7.34 (m, 1H), 7.34 – 7.24 (m, 2H), 2.11 – 1.89 (m, 4H), 1.39 – 1.35 (m, 12H), 0.98 – 0.56 (m, 22H), 0.55 – 0.36 (m, 8H).

^{13}C-NMR (100 MHz, $CDCl_3$): δ [ppm] = 151.21, 151.15, 151.05, 149.80, 149.72, 149.64, 144.37, 144.34, 144.31, 141.29, 141.24, 141.19, 134.87, 133.67, 131.38, 130.51, 130.45, 130.41, 127.83, 126.98, 126.77, 124.31, 124.27, 124.23, 120.15, 120.13, 120.12, 118.95, 118.93, 83.67, 54.97, 54.91, 54.88, 44.62, 44.54, 44.23, 44.17, 34.74, 34.69, 33.84, 33.81, 33.62, 33.60, 28.31, 28.29, 27.90, 27.41, 27.40, 26.95, 26.92, 25.06, 24.99, 24.94, 22.85, 22.83, 14.22, 14.21, 14.13, 10.63, 10.22. (Multiple Peaks are observed due to the presence of diastereomers)

HR-MS (APCI pos): [m/z] for $C_{35}H_{53}BO_2$ = 516.41572 (found), 516.41386 (calculated).

Analytical data is in accordance with literature.[12]

Synthesis of 7-bromo-9,9,9',9'-tetrakis(2-ethylhexyl)-9H,9'H-2,2'-bifluorene

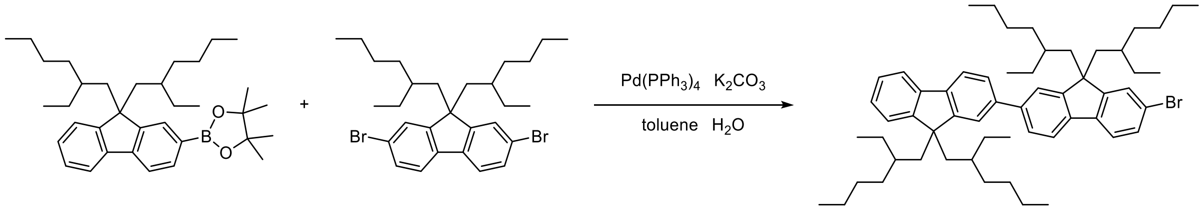

This reaction is performed according to general procedure C using 2-(9,9-bis(2-ethylhexyl)-9H-fluoren-2-yl)-4,4,5,5-tetramethyl-1,3,2-dioxaborolane (2.00 g, 3.87 mmol, 1 eq.), 2,7-dibromo-9,9-bis(2-ethylhexyl)-9H-fluorene (5.31 g, 9.68 mmol, 2.5 eq.), tetrakis(triphenylphosphine)palladium(0) (224 mg, 194 µmol, 5 mol%) and potassium carbonate (1.07 g, 7.74 mmol, 2 eq.) in a solvent mixture of water (9 ml) and toluene (45 ml). After column chromatography (SiO_2, petroleum ether), the desired product is obtained as a colorless oil (1.73 g, 2.02 mmol, 52%).

^{1}H-NMR (400 MHz, $CDCl_3$): δ [ppm] = 7.78 – 7.70 (m, 3H), 7.65 – 7.52 (m, 5H), 7.50 – 7.42 (m, 2H), 7.42 – 7.26 (m, 3H), 2.13 – 1.91 (m, 8H), 1.02 – 0.60 (m, 44H), 0.60 – 0.45 (m, 16H).

^{13}C-NMR (100 MHz, $CDCl_3$): δ [ppm] = 153.17, 153.14, 151.17, 151.12, 151.11, 150.76, 150.74, 150.70, 150.70, 150.67, 142.11, 141.15, 141.04, 140.97, 140.93, 140.69, 140.68, 140.24, 140.19, 140.11, 139.60, 139.34, 139.32, 130.04, 128.94, 128.90, 128.85, 128.81, 127.61, 127.52, 127.41, 127.29, 126.94, 126.91, 126.58, 126.41, 126.39, 126.28, 126.14, 126.04, 124.28, 124.23, 124.18, 123.08, 123.04, 122.97, 122.93, 122.78, 121.13, 121.08, 120.54, 120.49, 120.09, 120.02, 120.00, 119.92, 119.90, 119.77, 55.37, 55.09, 44.72, 44.68, 44.51, 44.36, 34.86, 34.83, 34.81, 34.77, 34.75, 34.72, 34.20, 34.15, 34.03, 33.99, 33.91, 28.37, 28.33, 28.30, 28.24, 27.23, 27.02, 22.89, 22.87, 22.83, 14.20, 14.15, 14.08, 10.54, 10.51, 10.47, 10.43, 10.41, 10.38, 10.34. (Multiple Peaks are observed due to the presence of diastereomers)

HR-MS (MALDI pos): [m/z] for $C_{58}H_{81}Br$ = 858.55226 (found), 858.55012 (calculated).

Analytical data is in accordance with literature.[13]

Synthesis of 4,7-bis(9,9,9',9'-(2-ethylhexyl)-9H,9'H-[2,2'-bifluoren]-7-yl)benzo[c][1,2,5]thiadiazole (EH)

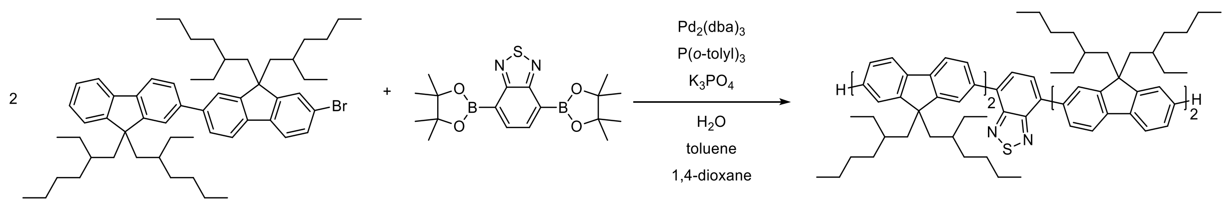

This reaction is performed according to general procedure D using 7-bromo-9,9,9',9'-tetrakis(2-ethylhexyl)-9H,9'H-2,2'-bifluorene (1.20 g, 1.40 mmol, 2 eq.), 4,7-bis-pinacolato-diborane-2,1,3-benzothiadiazole (258 mg, 664 µmol, 0.95 eq.), tris(dibenzylideneacetone)dipalladium(0) (9.60 mg, 10.5 µmol, 3 mol% Pd), tris(o-tolyl)phosphine (25.5 mg, 83.9 µmol, 12 mol%) and potassium phosphate (297 mg, 1.40 µmol, 2 eq.) in 48 ml of the solvent mixture. After column chromatography (SiO_2, petroleum ether/dichloromethane 95:5 → 9:1), the desired product is obtained as an orange solid (596 mg, 353 µmol, 50%).

^{1}H-NMR (400 MHz, $CDCl_3$): δ [ppm] = 8.09 – 8.02 (m, 4H), 7.91 (d, J = 7.9 Hz, 2H), 7.89 – 7.83 (m, 4H), 7.78 (d, J = 7.8 Hz, 2H), 7.73 (d, J = 7.4 Hz, 2H), 7.68 – 7.57 (m, 8H), 7.44 –

7.38 (m, 2H), 7.34 (t, J = 7.4 Hz, 2H), 7.29 (d, J = 7.3 Hz, 2H), 2.26 – 1.98 (m, 16H), 1.07 – 0.50 (m, 120H).

^{13}C-NMR (100 MHz, $CDCl_3$): δ [ppm] = 154.55, 151.58, 151.13, 150.77, 141.50, 141.23, 140.60, 140.11, 135.84, 133.80, 128.39, 127.87, 126.93, 126.54, 126.36, 126.08, 125.25, 125.15, 124.28, 124.24, 123.20, 123.12, 123.02, 120.19, 119.89, 119.78, 55.37, 55.11, 44.71, 34.88, 34.77, 34.74, 34.34, 34.20, 33.96, 28.38, 28.33, 27.34, 27.23, 27.18, 27.04, 22.96, 22.93, 22.90, 22.89, 22.84, 14.19, 14.16, 14.13, 10.55, 10.48, 10.43, 10.40, 10.37. (Multiple Peaks are observed due to the presence of diastereomers)

HR-MS (MALDI pos): [m/z] for $C_{122}H_{164}N_2S$ = 1690.26358 (found), 1690.26488 (calculated).

References

[1] P. J. Welscher, D. Straub, F. Stümpges, A. L. Respondek, B. Esser, A. J. C. Kuehne, *Adv. Funct. Mater.* **2024**, *2417129*, DOI 10.1002/adfm.202417129.

[2] Z. Zheng, F. Caraguel, Y. Y. Liao, C. Andraud, B. Van Der Sanden, Y. Bretonnière, *RSC Adv.* **2016**, *6*, 94200–94205.

[3] P. J. Welscher, U. Ziener, A. J. C. Kuehne, *(Preprint) ChemRxiv, 10.26434/chemrxiv-2024-ljtxr* **2024**, version 1, submitted: December, 2024.

[4] X. Feng, F. Lv, L. Liu, Q. Yang, S. Wang, G. C. Bazan, *Adv. Mater.* **2012**, *24*, 5428–5432.

[5] G. Saroja, Z. Pingzhu, N. P. Ernsting, J. Liebscher, *J. Org. Chem.* **2004**, *69*, 987–990.

[6] Y.-J. Pu, M. Higashidate, K. Nakayama, J. Kido, *J. Mater. Chem.* **2008**, *18*, 4183.

[7] G. Zhou, Y. He, B. Yao, J. Dang, W. Wong, Z. Xie, X. Zhao, L. Wang, *Chem. – An Asian J.* **2010**, *5*, 2405–2414.

[8] C.-C. Chang, H. Yueh, C.-T. Chen, *Org. Lett.* **2011**, *13*, 2702–2705.

[9] W. Y. Lai, R. Xia, D. D. C. Bradley, W. Huang, *Chem. - A Eur. J.* **2010**, *16*, 8471–8479.

[10] A. L. Kanibolotsky, R. Berridge, P. J. Skabara, I. F. Perepichka, D. D. C. Bradley, M. Koeberg, *J. Am. Chem. Soc.* **2004**, *126*, 13695–13702.

[11] L. Hu, Y. Zhang, T. Guo, L. Ying, J. Xiong, W. Yang, Y. Cao, *Dye. Pigment.* **2019**, *166*, 502–514.

[12] Y. B. Huang, Y. Cui, L. F. Zeng, Y. Y. Shen, L. Z. Feng, X. F. Shi, L. Y. Ye, Y. W. Yin, S. Tu, *J. Fluor. Chem.* **2015**, *178*, 195–201.

[13] J. Jo, C. Chi, S. Höger, G. Wegner, D. Y. Yoon, *Chem. - A Eur. J.* **2004**, *10*, 2681–2688.

Structural characterization

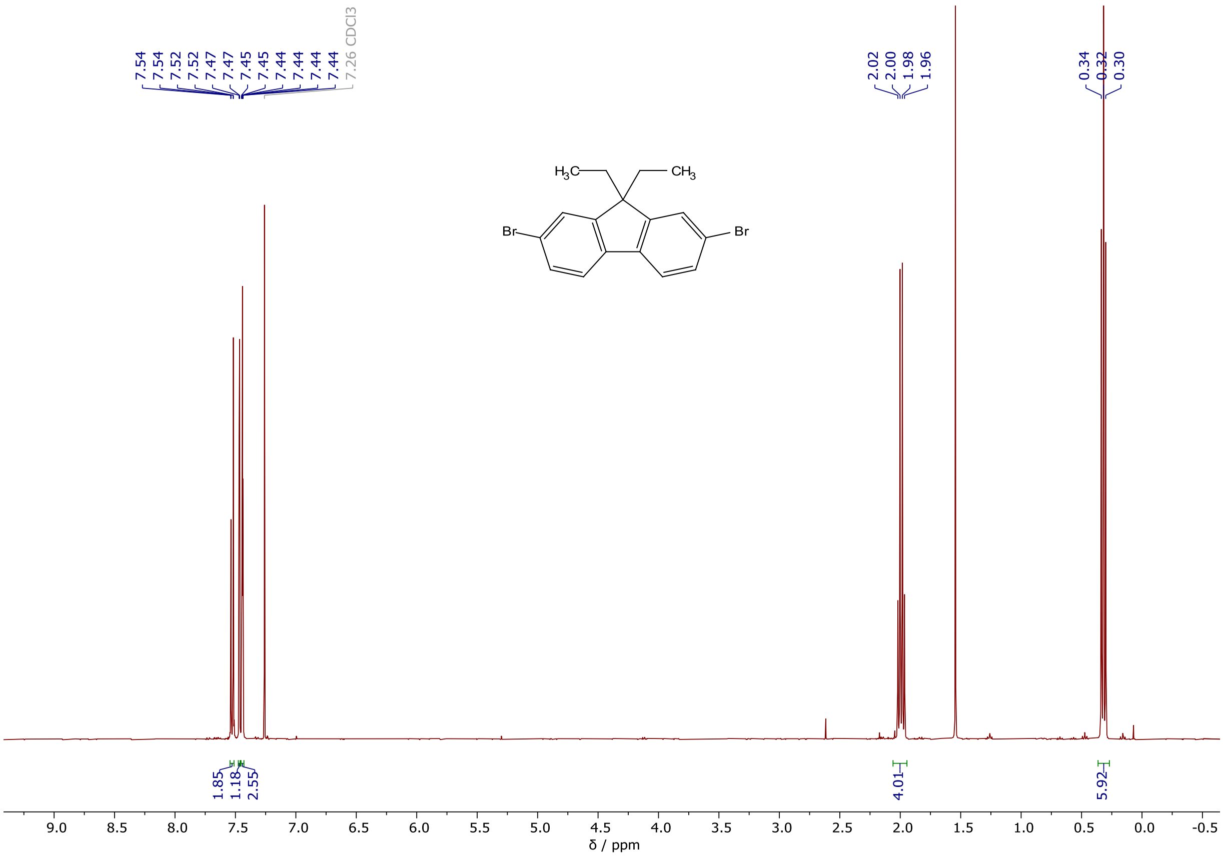

Figure S8: ^{1}H-NMR of 2,7-dibromo-9,9-diethyl-9H-fluorene in $CDCl_3$.

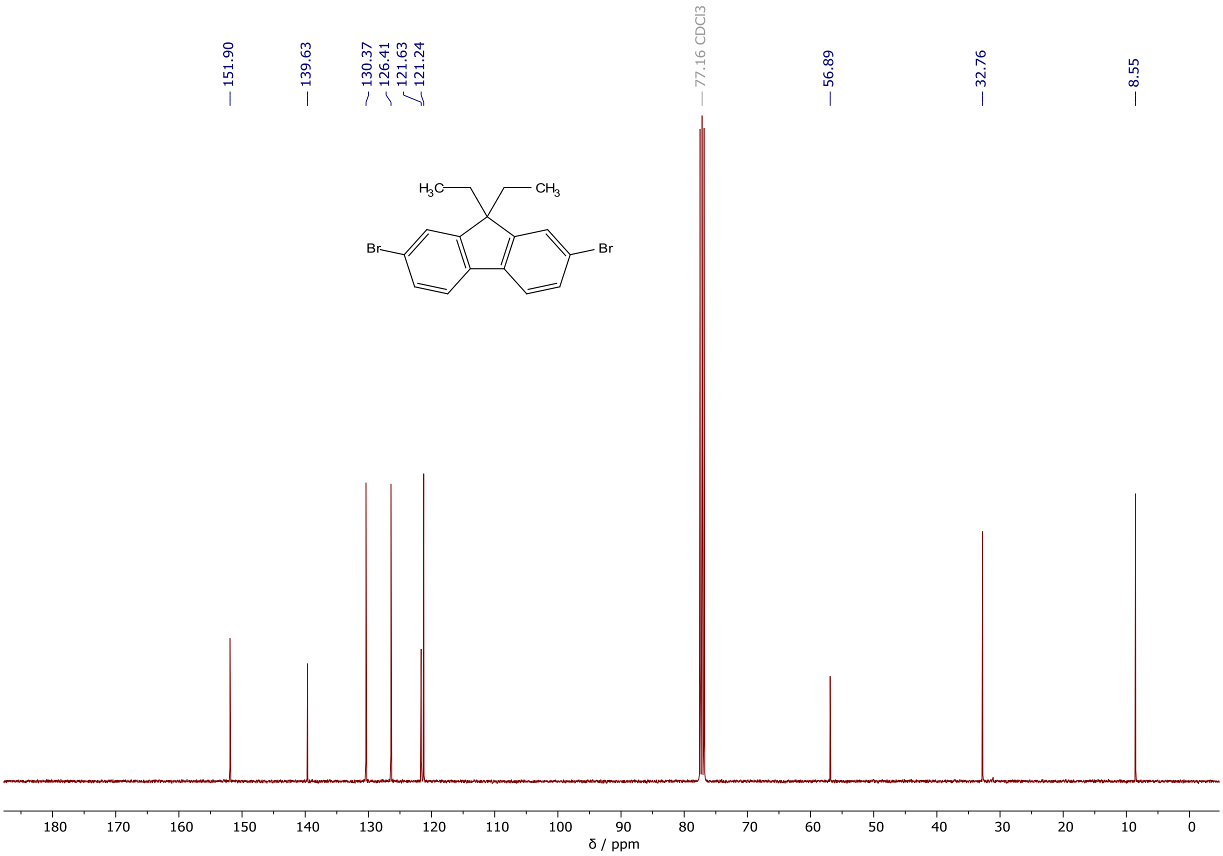

Figure S9: ^{13}C-NMR of 2,7-dibromo-9,9-diethyl-9H-fluorene in $CDCl_3$.

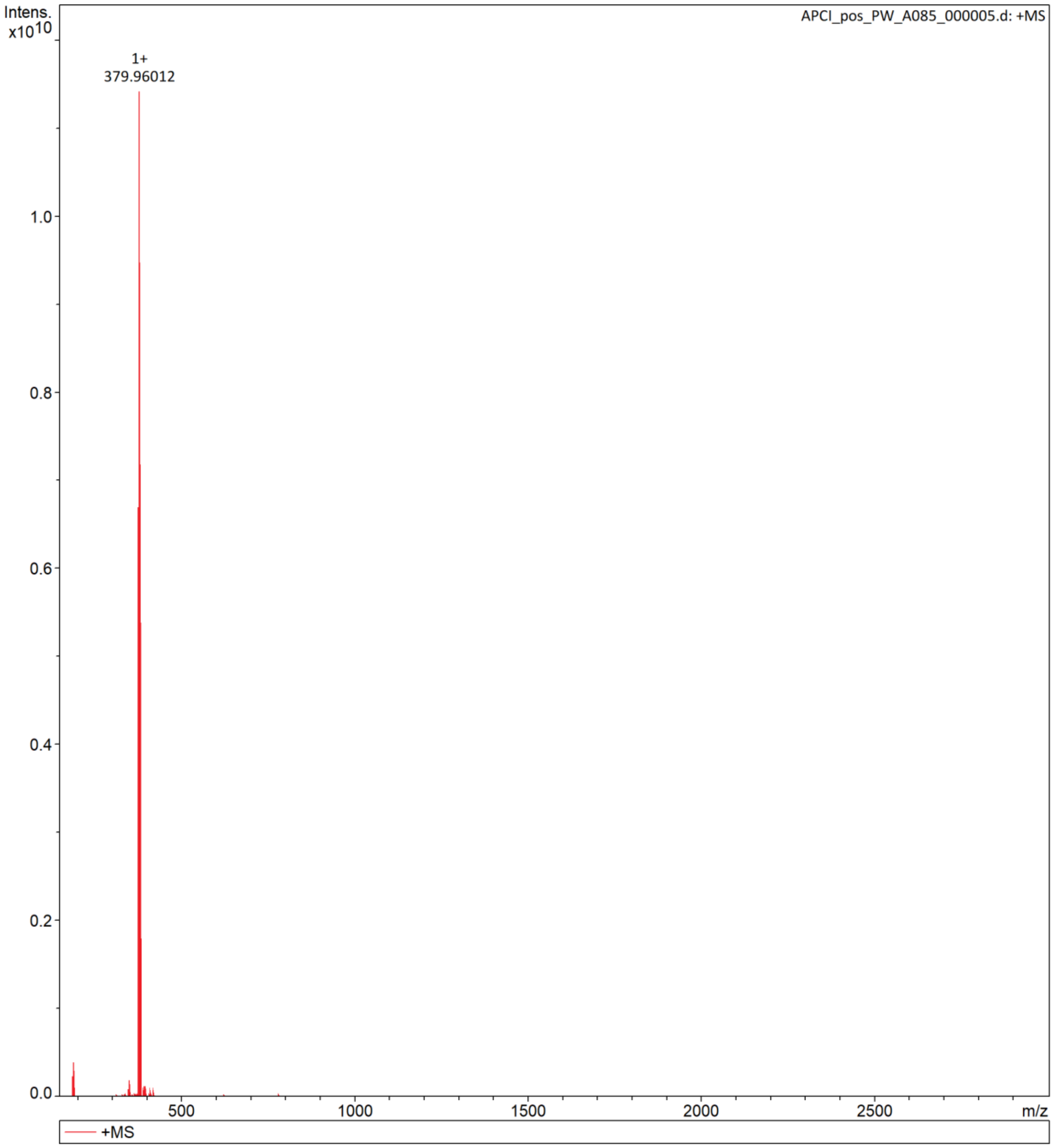

Figure S10: HR-APCI mass spectrum of 2,7-dibromo-9,9-diethyl-9H-fluorene.

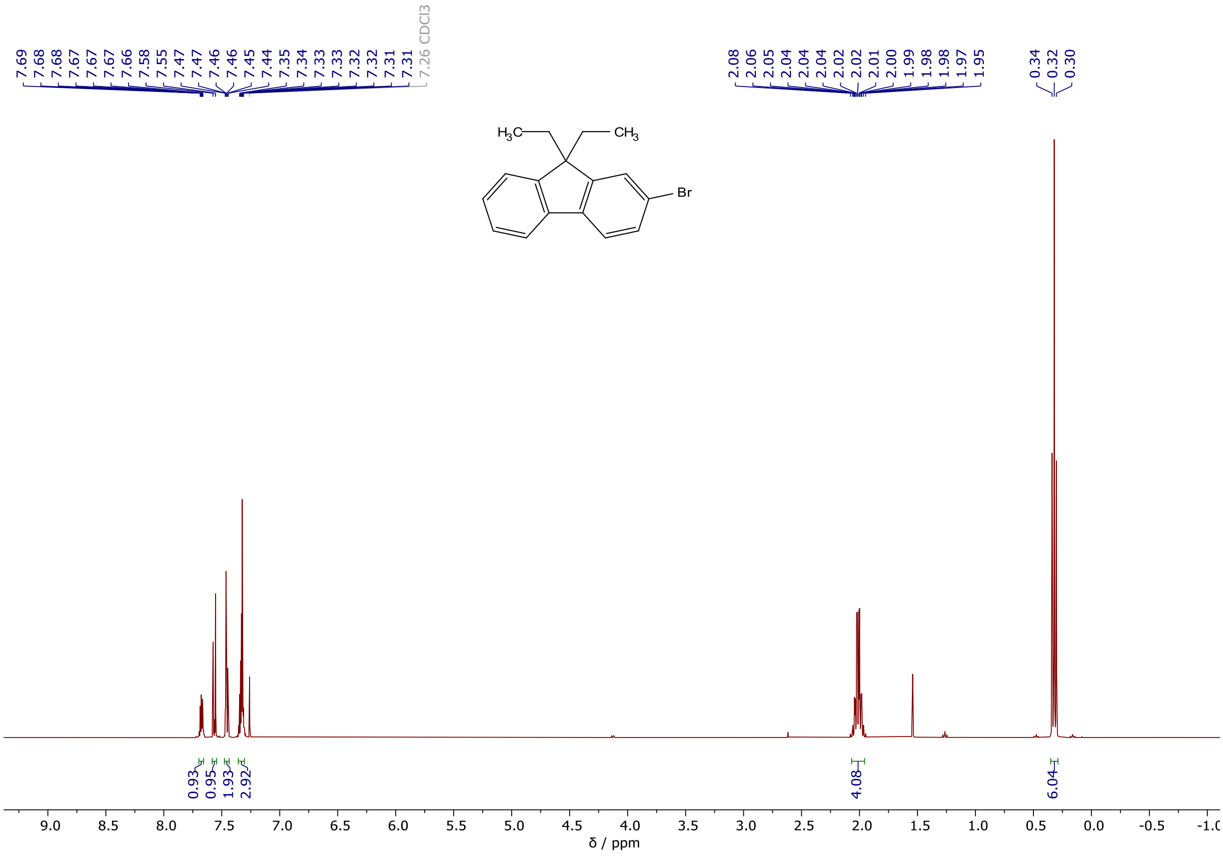

Figure S11: ^{1}H-NMR of 2-bromo-9,9-diethyl-9H-fluorene in $CDCl_3$.

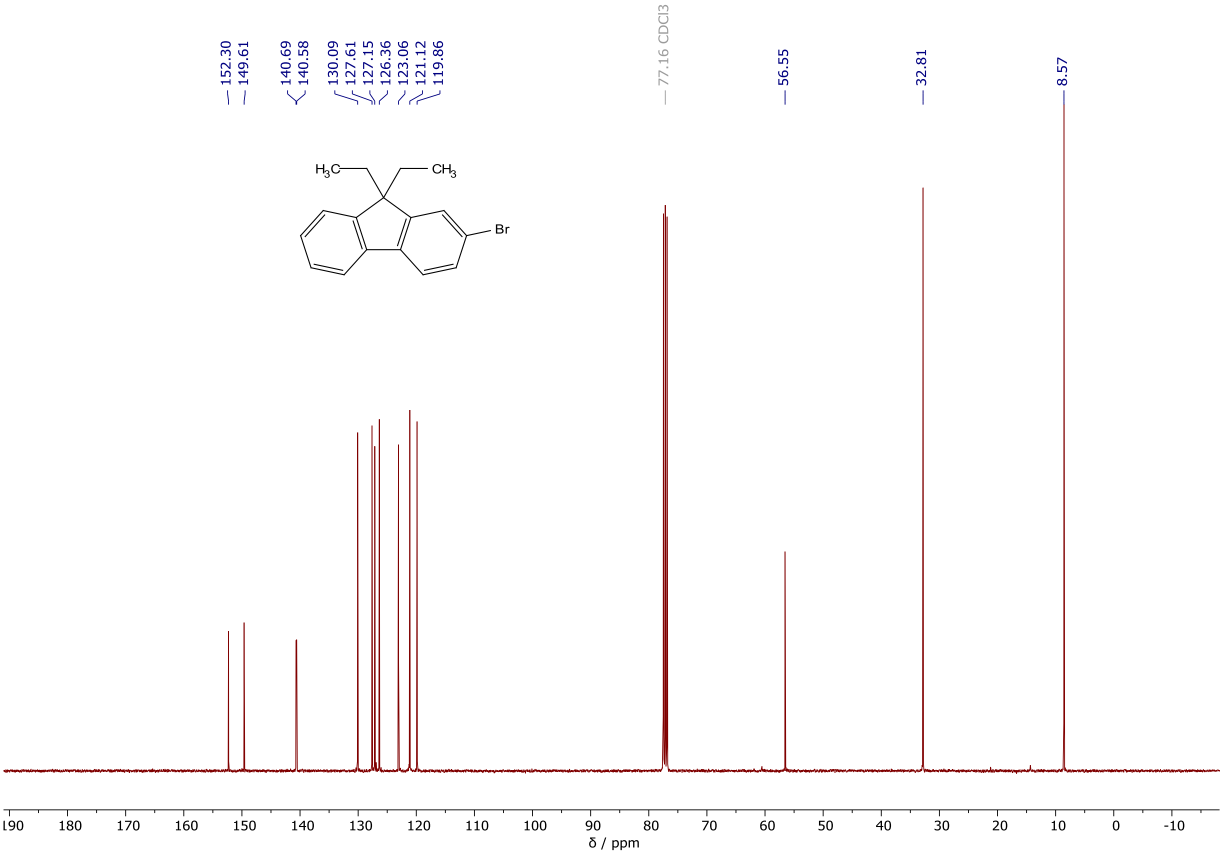

Figure S12: ^{13}C-NMR of 2-bromo-9,9-diethyl-9H-fluorene in $CDCl_3$.

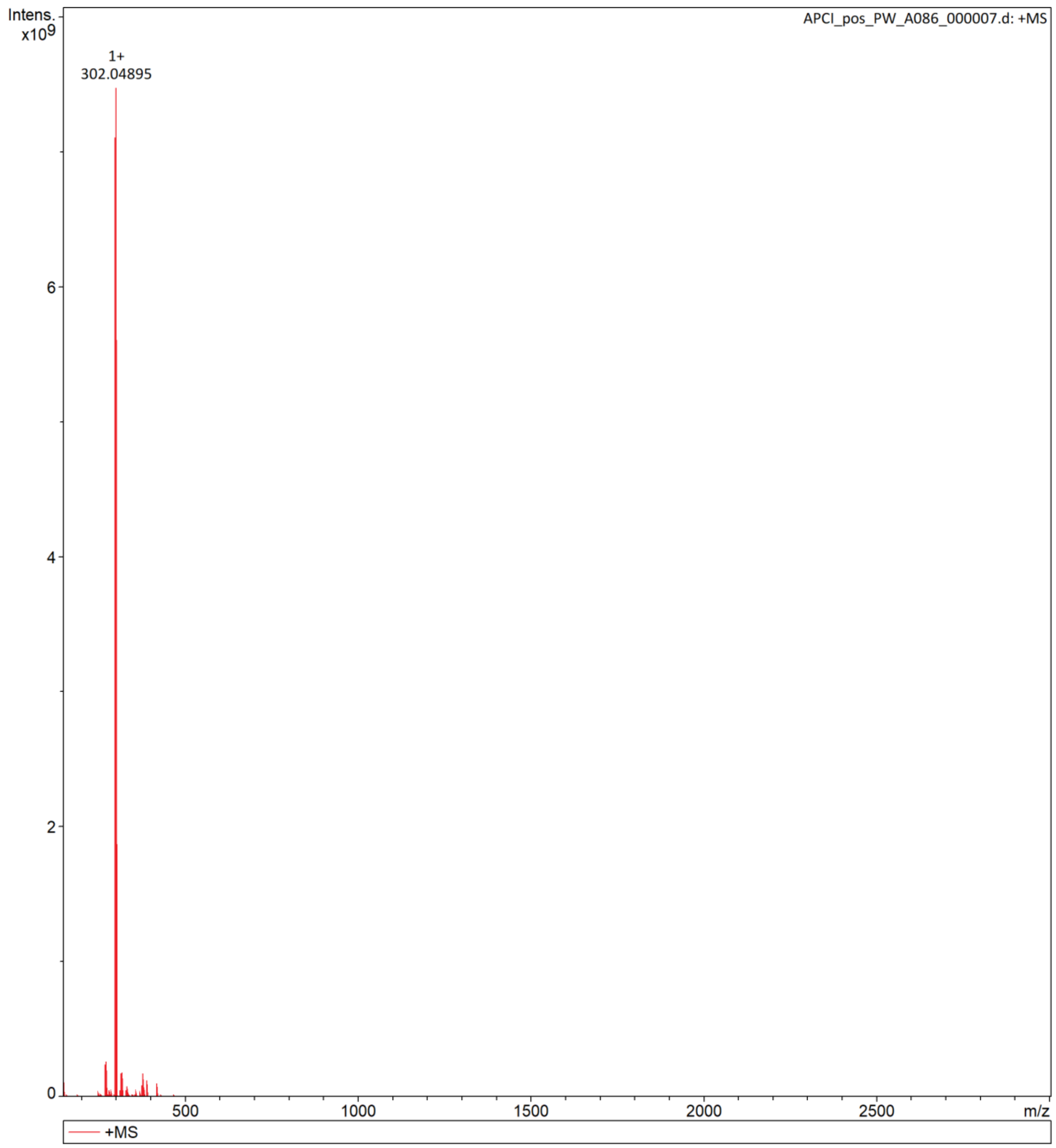

Figure S13: HR-APCI mass spectrum of 2-bromo-9,9-diethyl-9H-fluorene.

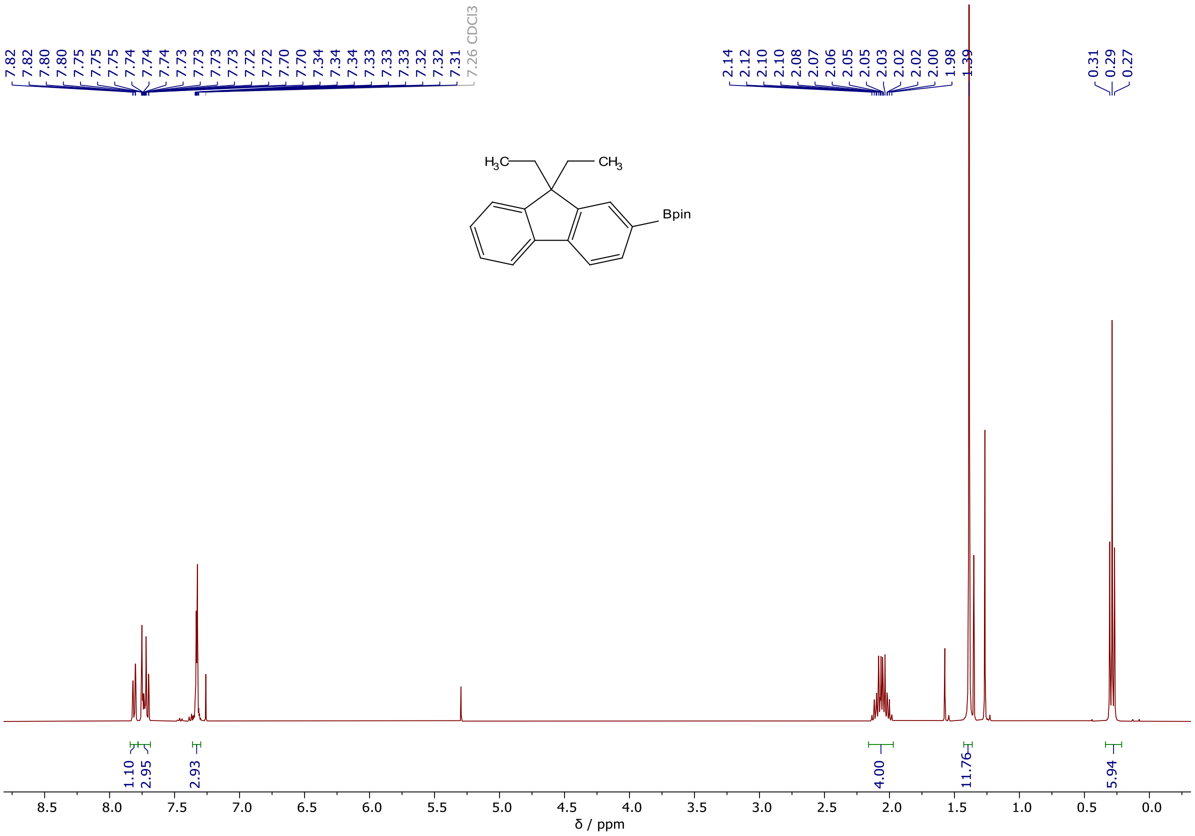

Figure S14: ^{1}H-NMR of 2-(9,9-diethyl-9H-fluoren-2-yl)-4,4,5,5-tetramethyl-1,3,2-dioxaborolane in $CDCl_3$.

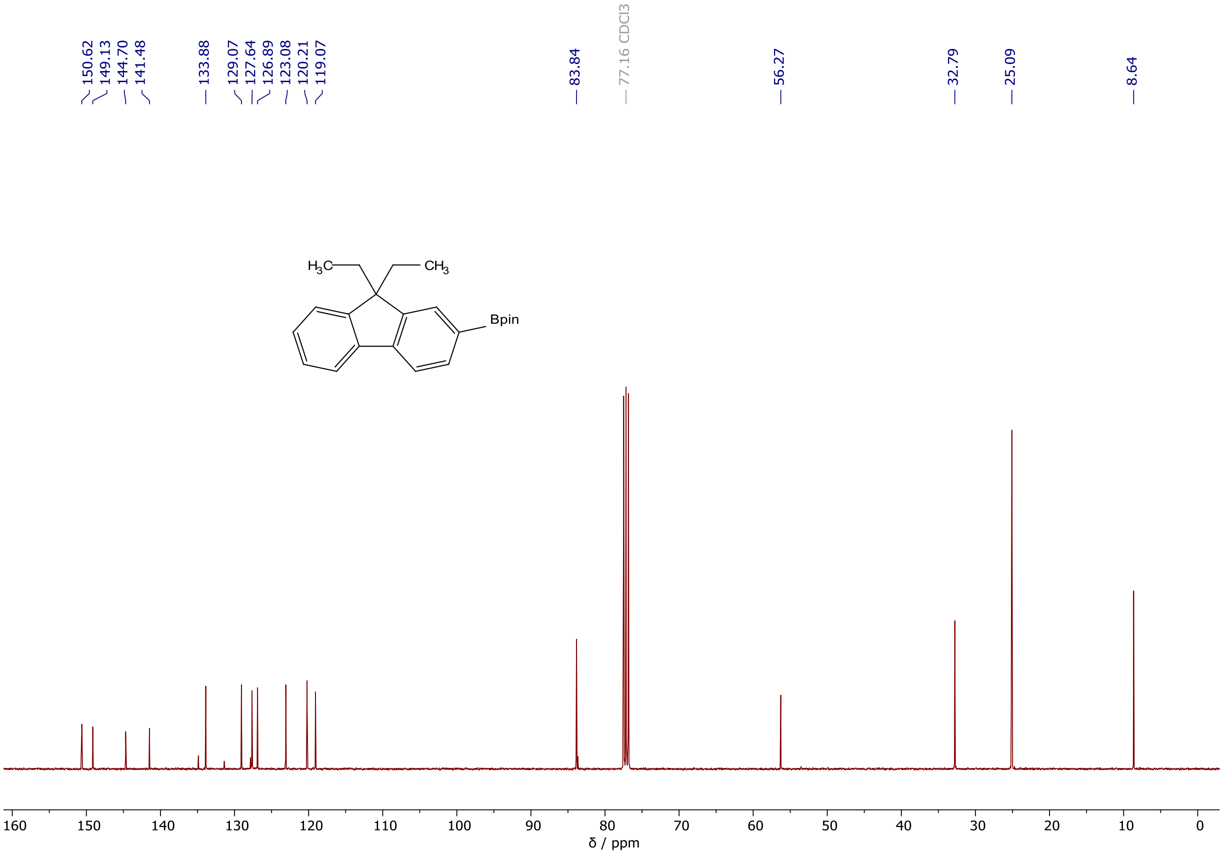

Figure S15: ^{13}C-NMR of 2-(9,9-diethyl-9H-fluoren-2-yl)-4,4,5,5-tetramethyl-1,3,2-dioxaborolane in $CDCl_3$.

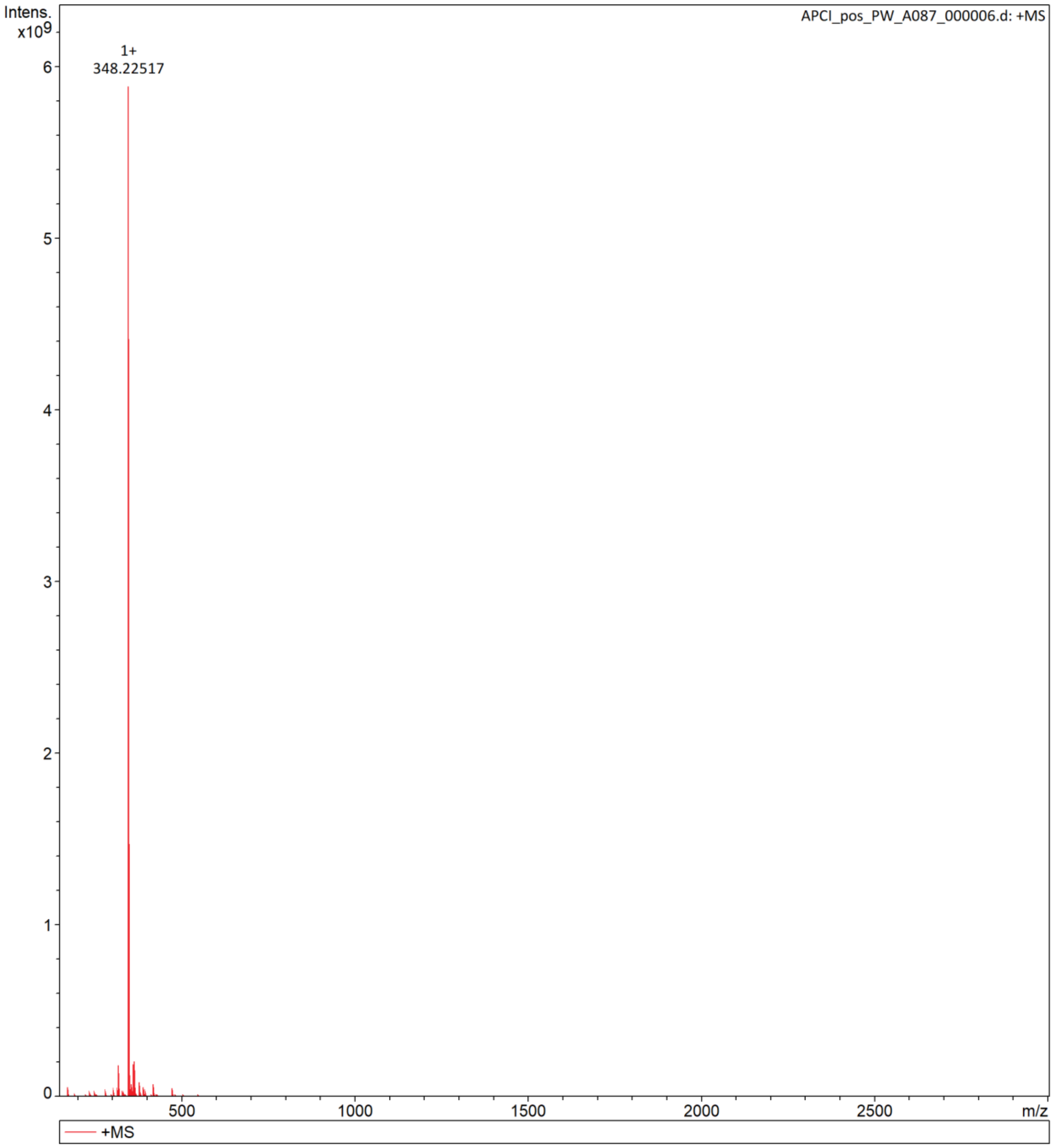

Figure S16: HR-APCI mass spectrum of 2-(9,9-diethyl-9H-fluoren-2-yl)-4,4,5,5-tetramethyl-1,3,2-dioxaborolane.

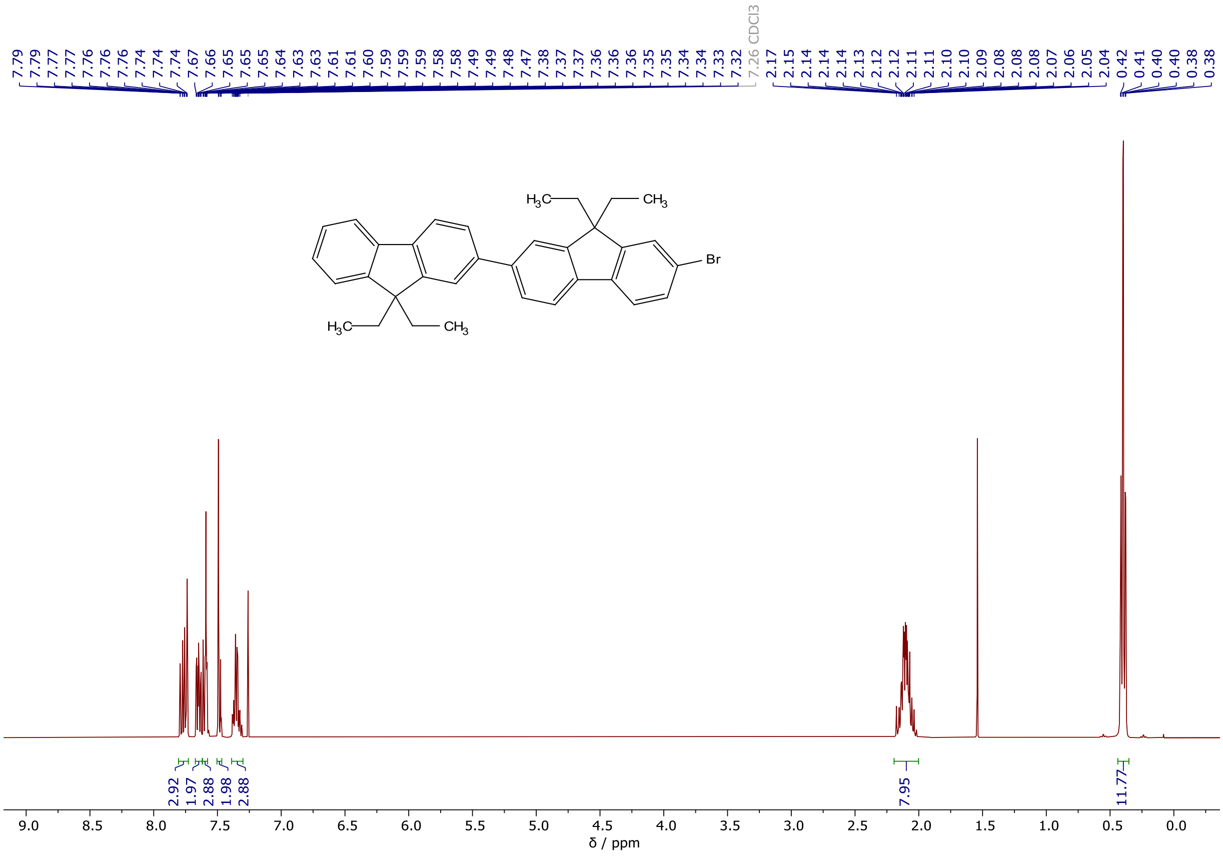

Figure S17: ^{1}H-NMR of 7-bromo-9,9,9',9'-tetraethyl-9H,9H'-2,2'-bifluorene in $CDCl_3$.

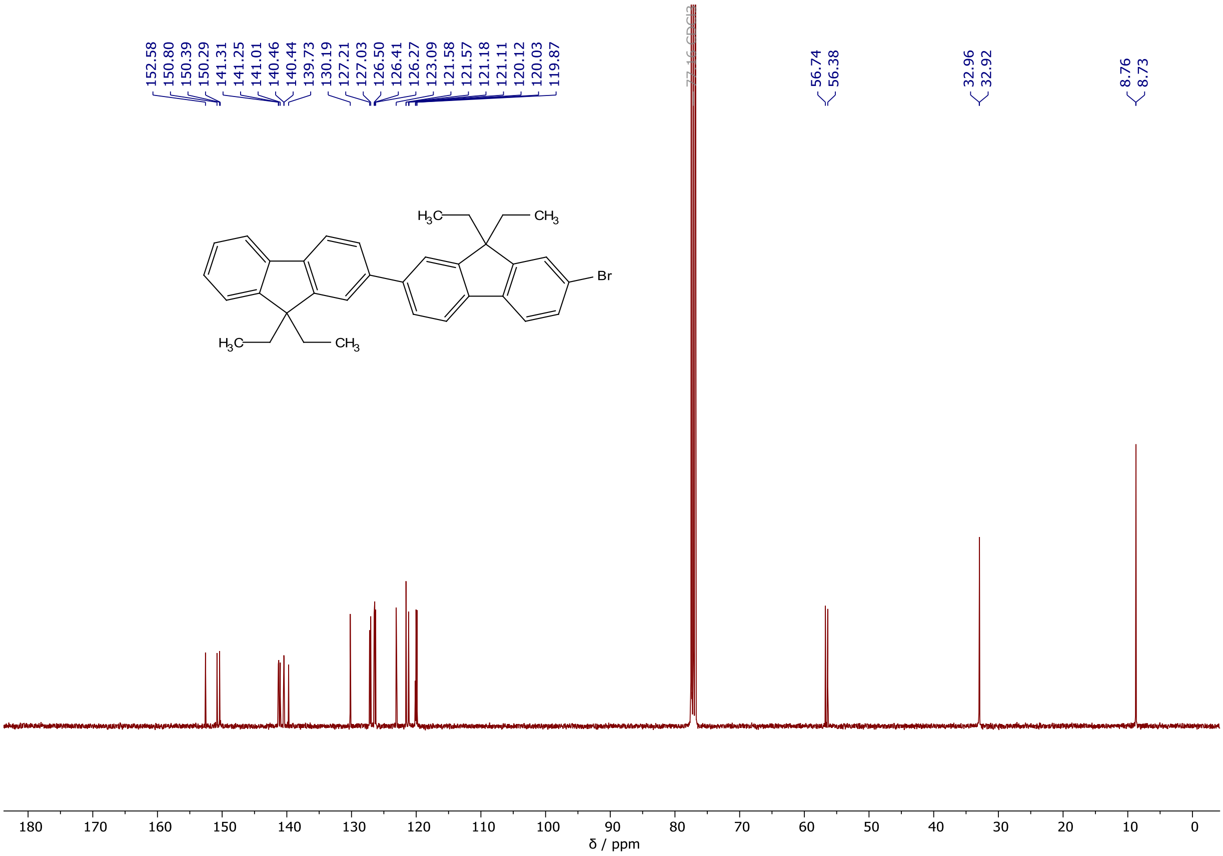

Figure S18: ^{13}C-NMR of 7-bromo-9,9,9',9'-tetraethyl-9H,9H'-2,2'-bifluorene in $CDCl_3$.

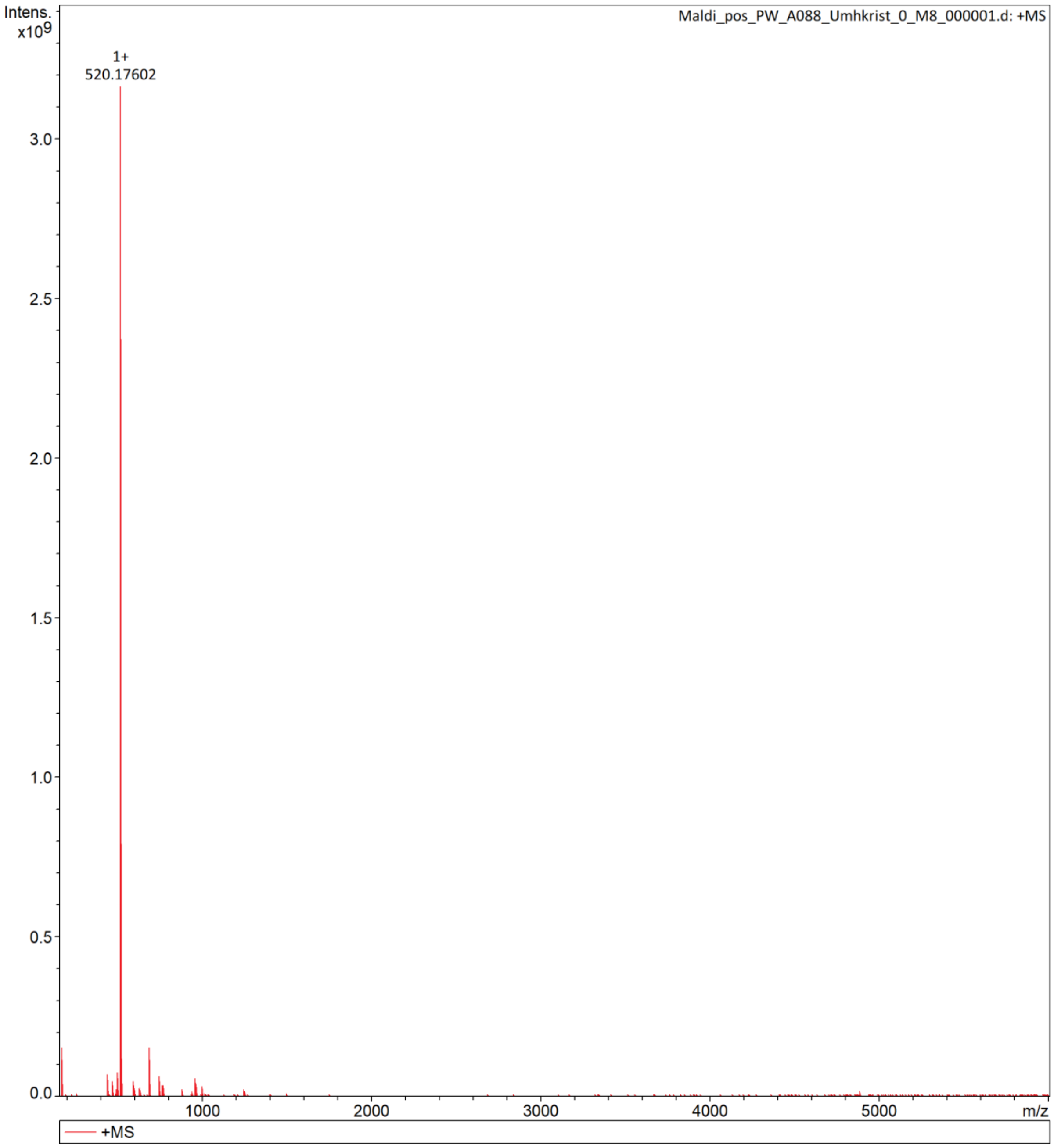

Figure S19: HR-MALDI mass spectrum of 7-bromo-9,9,9’,9’-tetraethyl-9H,9H’-2,2’-bifluorene with DCTB as matrix.

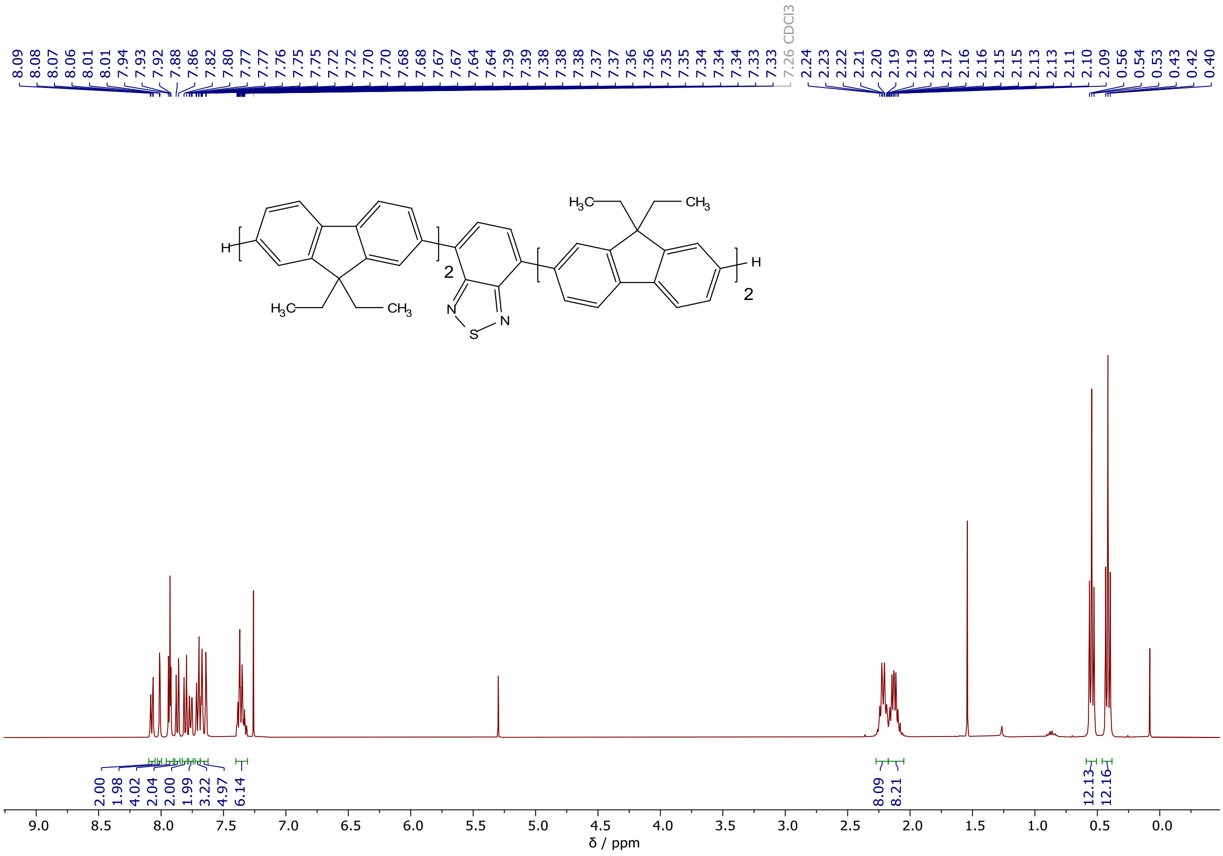

Figure S20: ^{1}H-NMR of 4,7-bis(9,9,9',9'-tetraethyl-9H,9'H-[2,2'-bifluoren]-7-yl)benzo[c][1,2,5]thiadi-azole (C2) in $CDCl_3$.

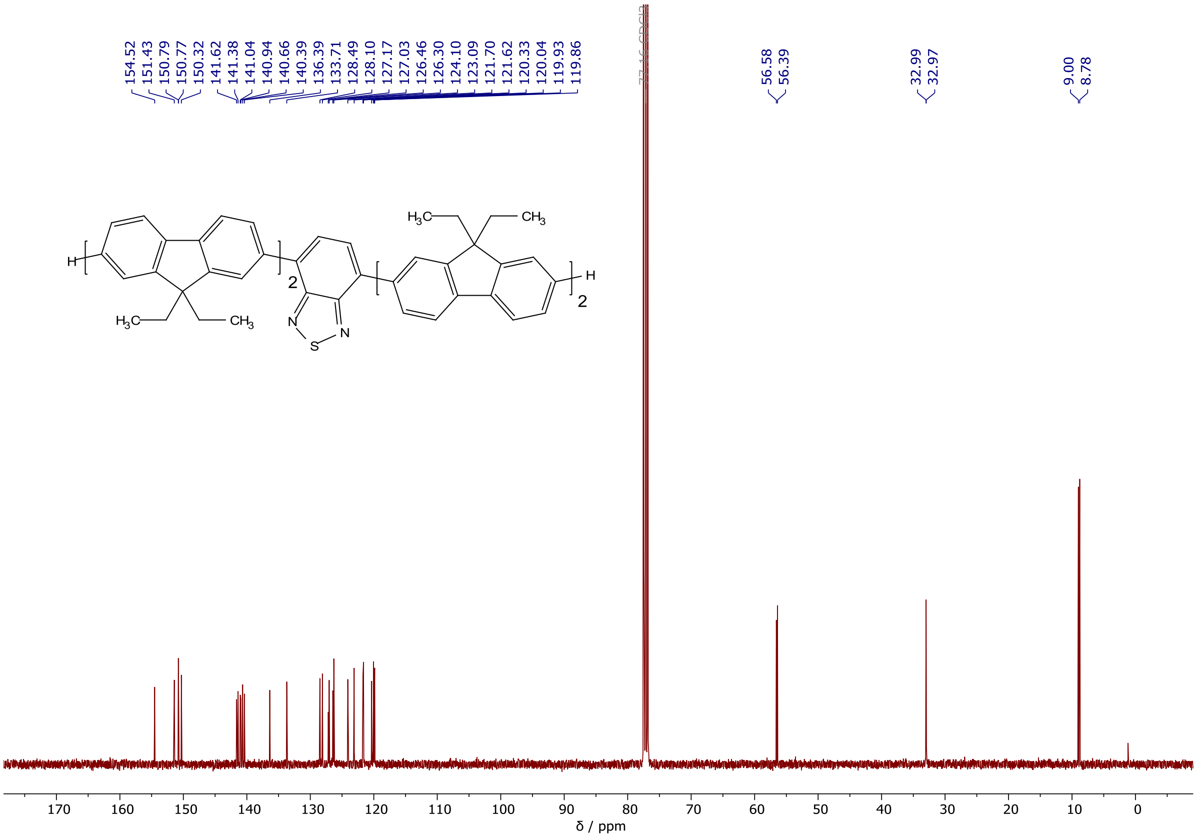

Figure S21: ^{13}C-NMR of 4,7-bis(9,9,9',9'-tetraethyl-9H,9'H-[2,2'-bifluoren]-7-yl)benzo[c][1,2,5]thiadi-azole (C2) in $CDCl_3$.

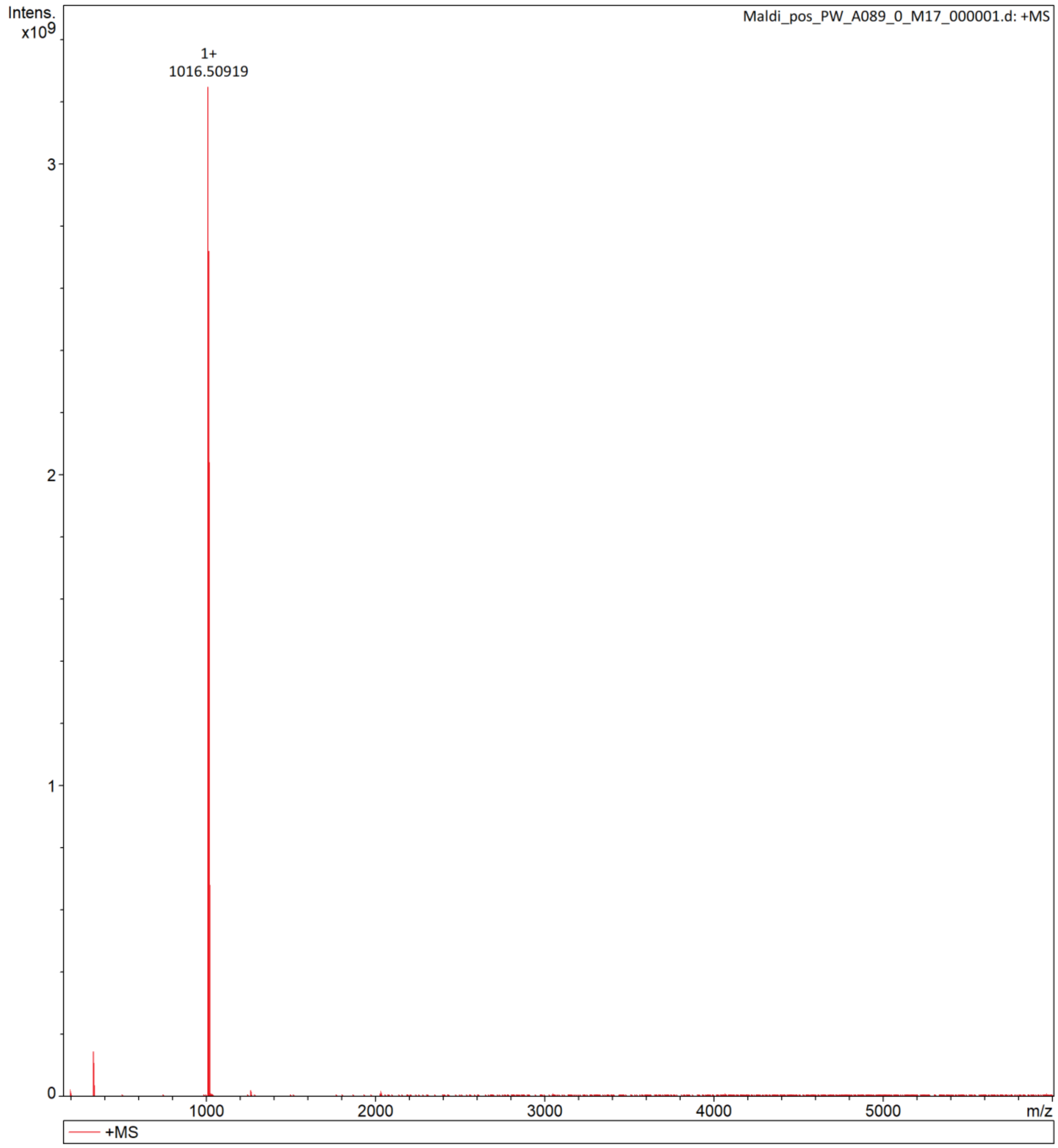

Figure S22: HR-MALDI mass spectrum of 4,7-bis(9,9,9',9'-tetraethyl-9H,9'H-[2,2'-bifluoren]-7-yl)benzo[c][1,2,5]thiadi-azole (C2) with DCTB as matrix.

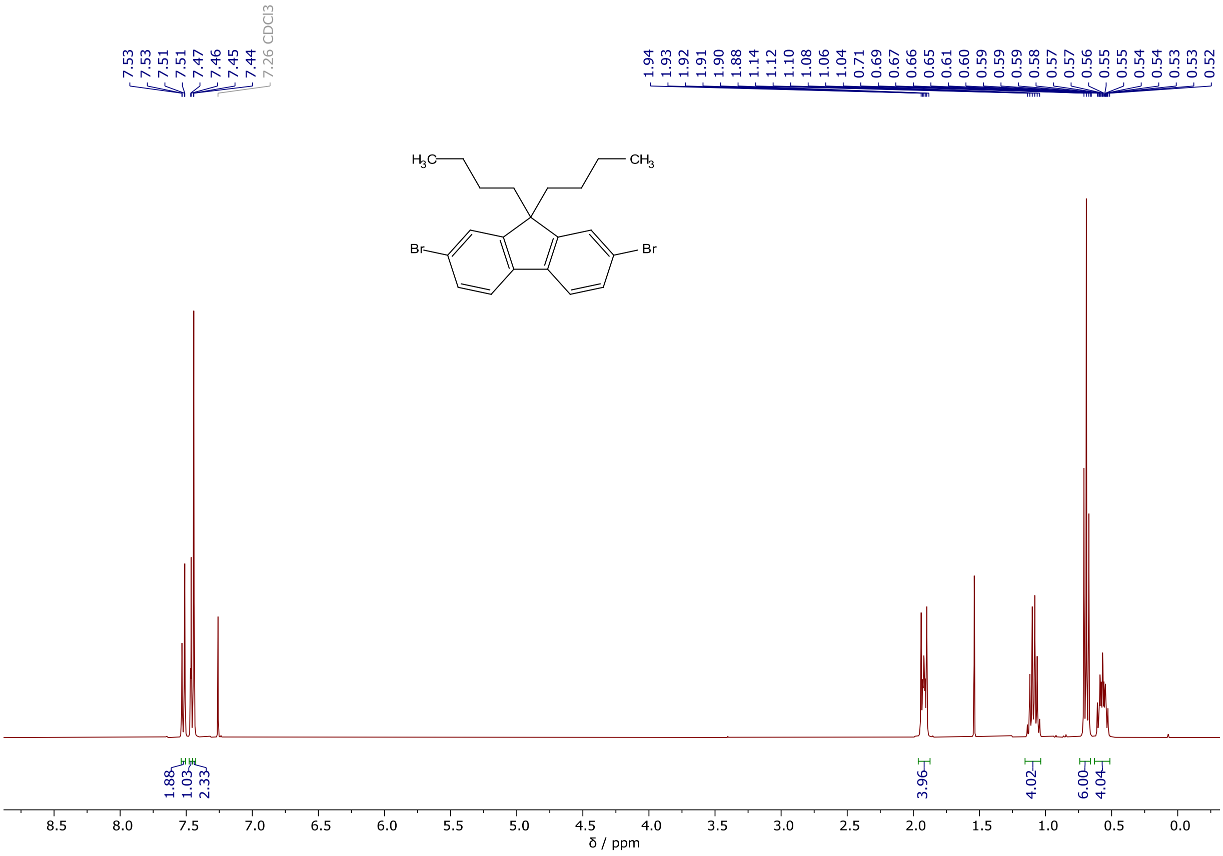

Figure S23: ^{1}H-NMR of 2,7-dibromo-9,9-dibutyl-9H-fluorene in $CDCl_3$.

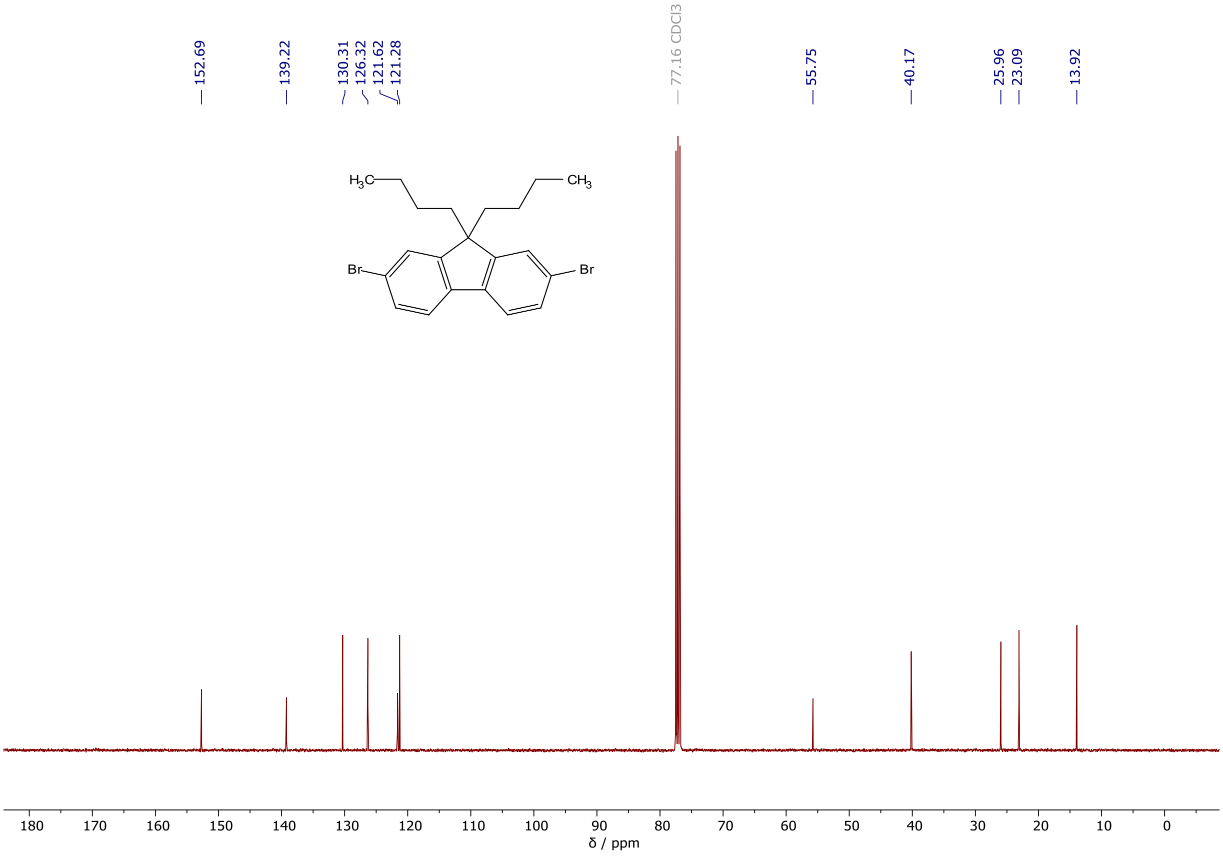

Figure S24: ^{13}C-NMR of 2,7-dibromo-9,9-dibutyl-9H-fluorene in $CDCl_3$.

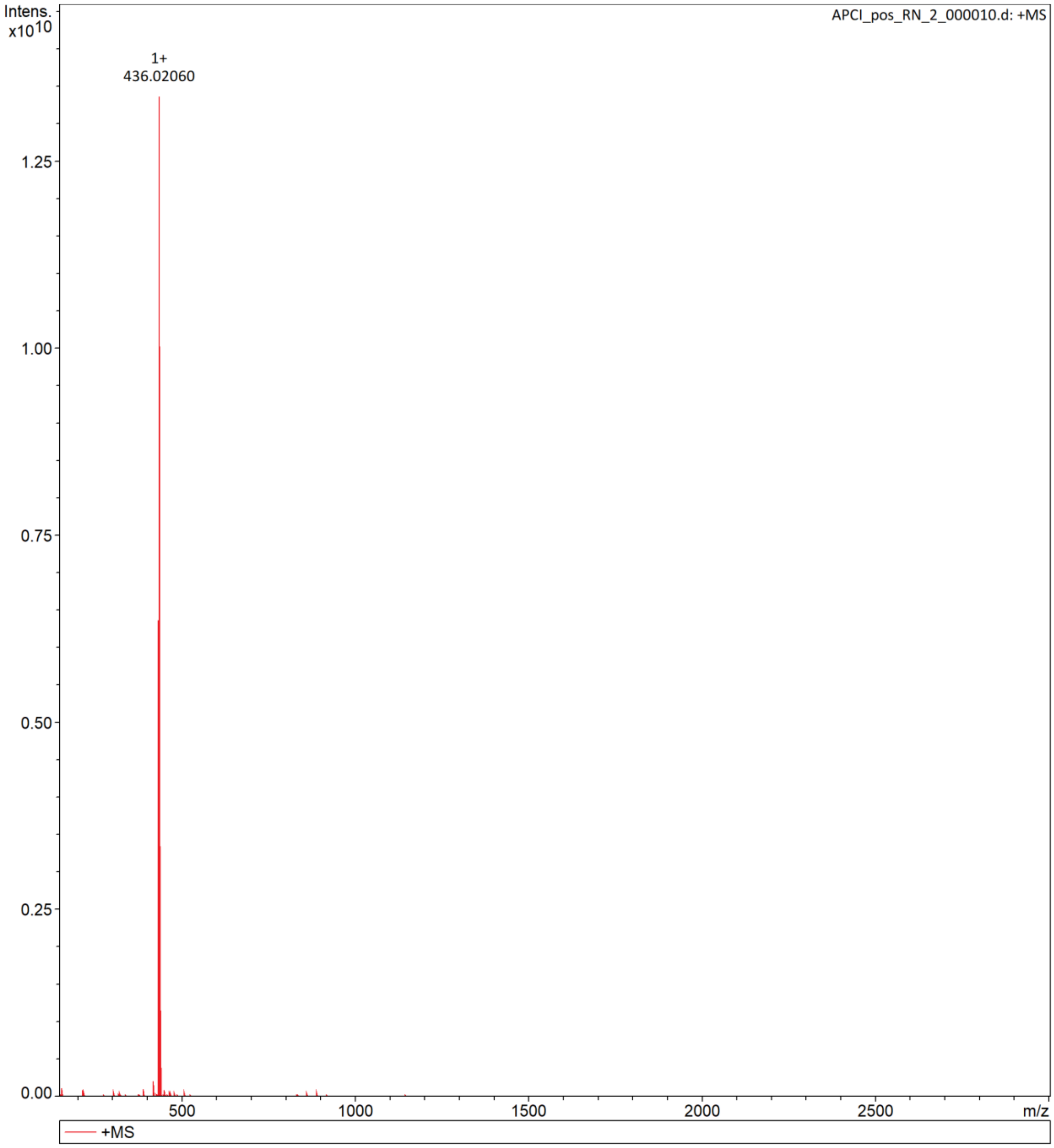

Figure S25: HR-APCI mass spectrum of 2,7-dibromo-9,9-dibutyl-9H-fluorene.

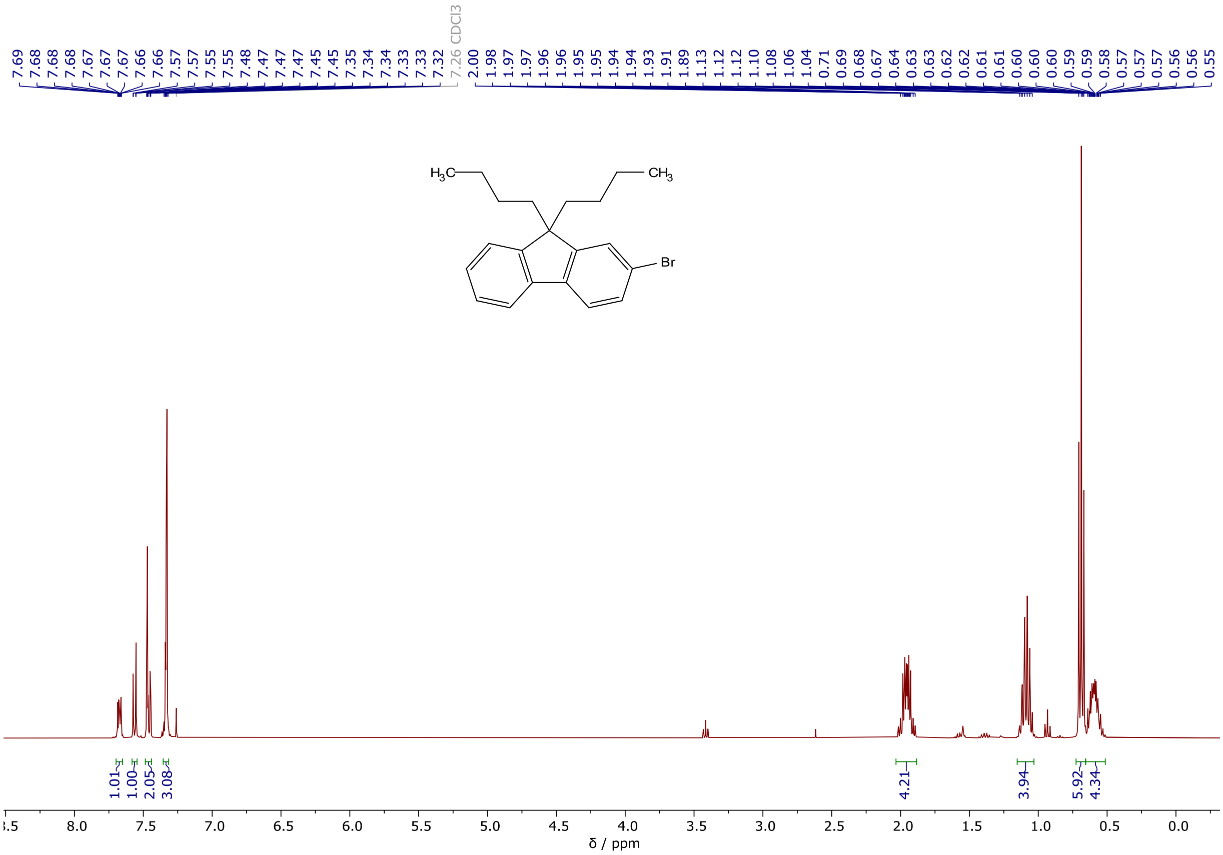

Figure S26: ^{1}H-NMR of 2-bromo-9,9-dibutyl-9H-fluorene in $CDCl_3$.

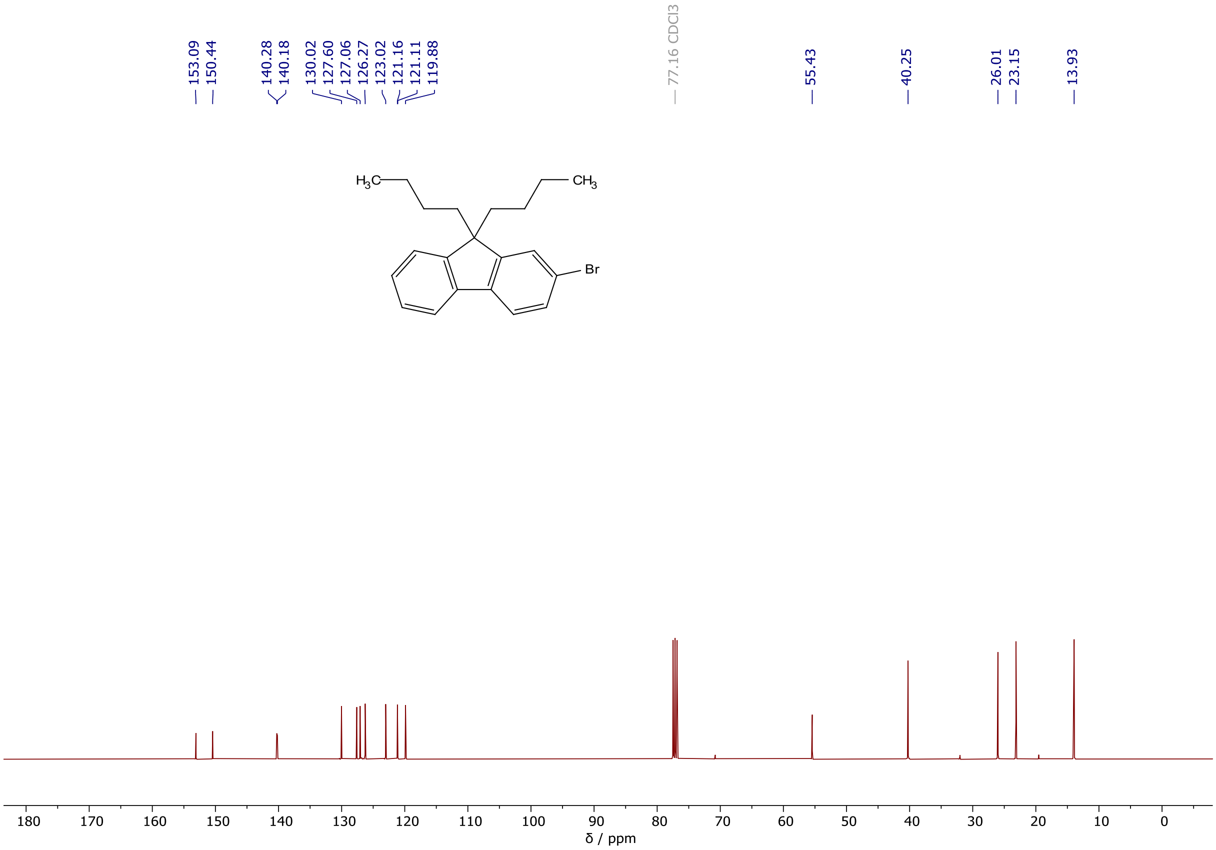

Figure S27: ^{13}C-NMR of 2-bromo-9,9-dibutyl-9H-fluorene in $CDCl_3$.

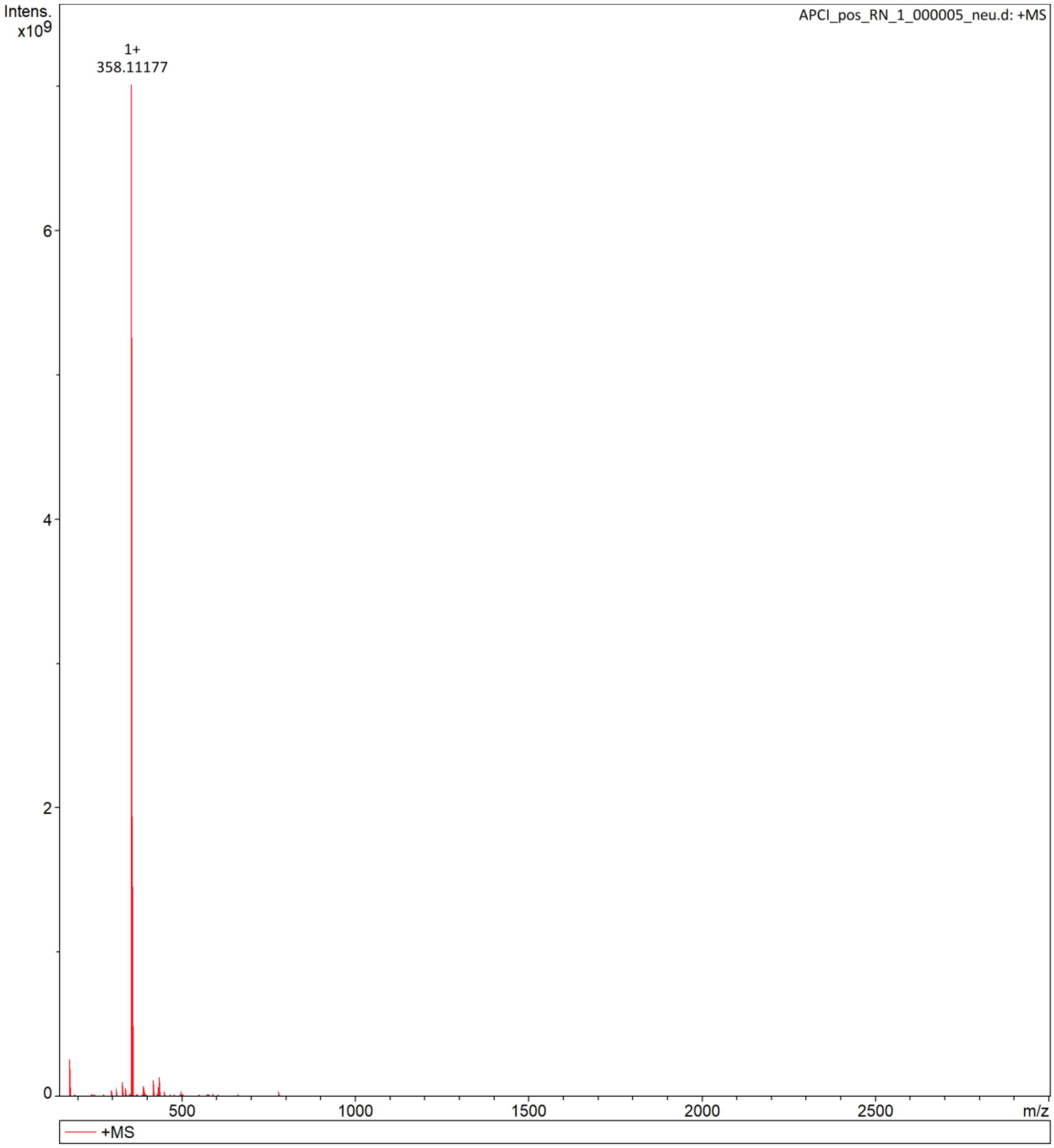

Figure S28: HR-APCI mass spectrum of 2-bromo-9,9-dibutyl-9H-fluorene.

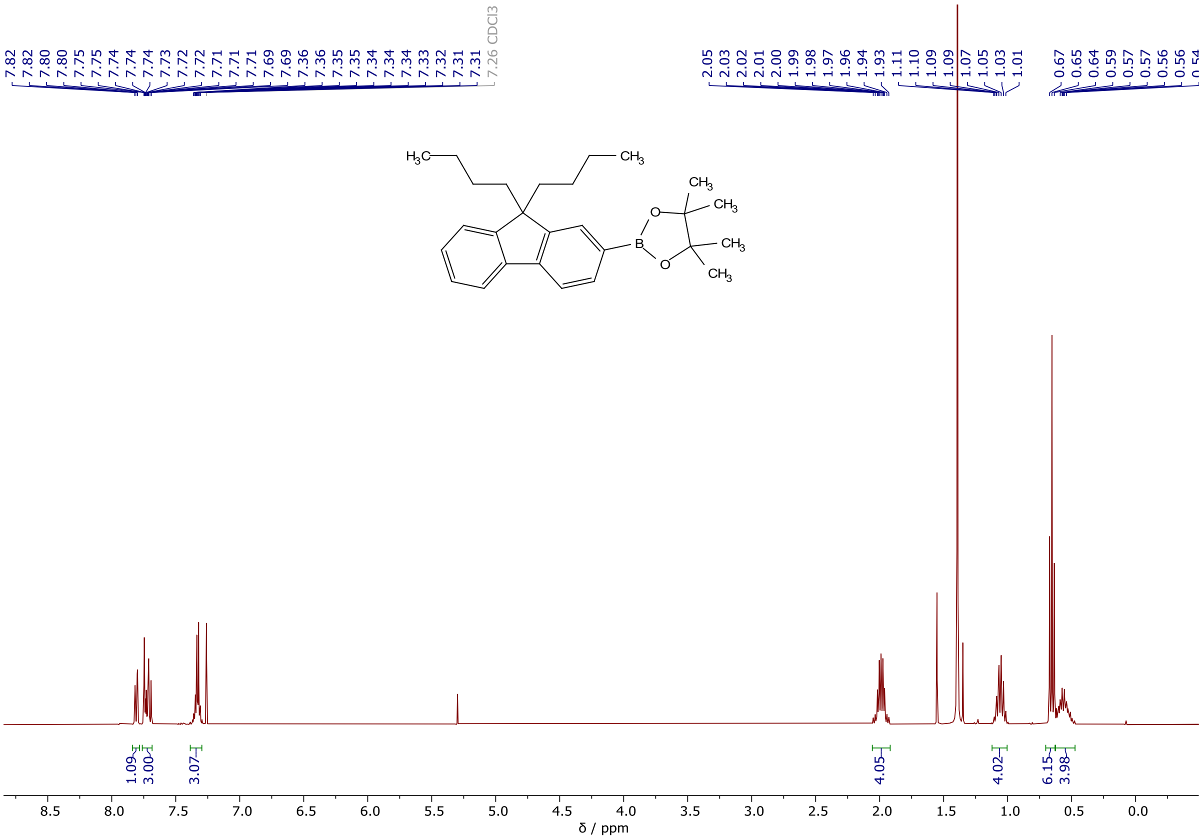

Figure S29: ^{1}H-NMR of 2-(9,9-dibutyl-9H-fluoren-2-yl)-4,4,5,5-tetramethyl-1,3,2-dioxaborolane in $CDCl_3$.

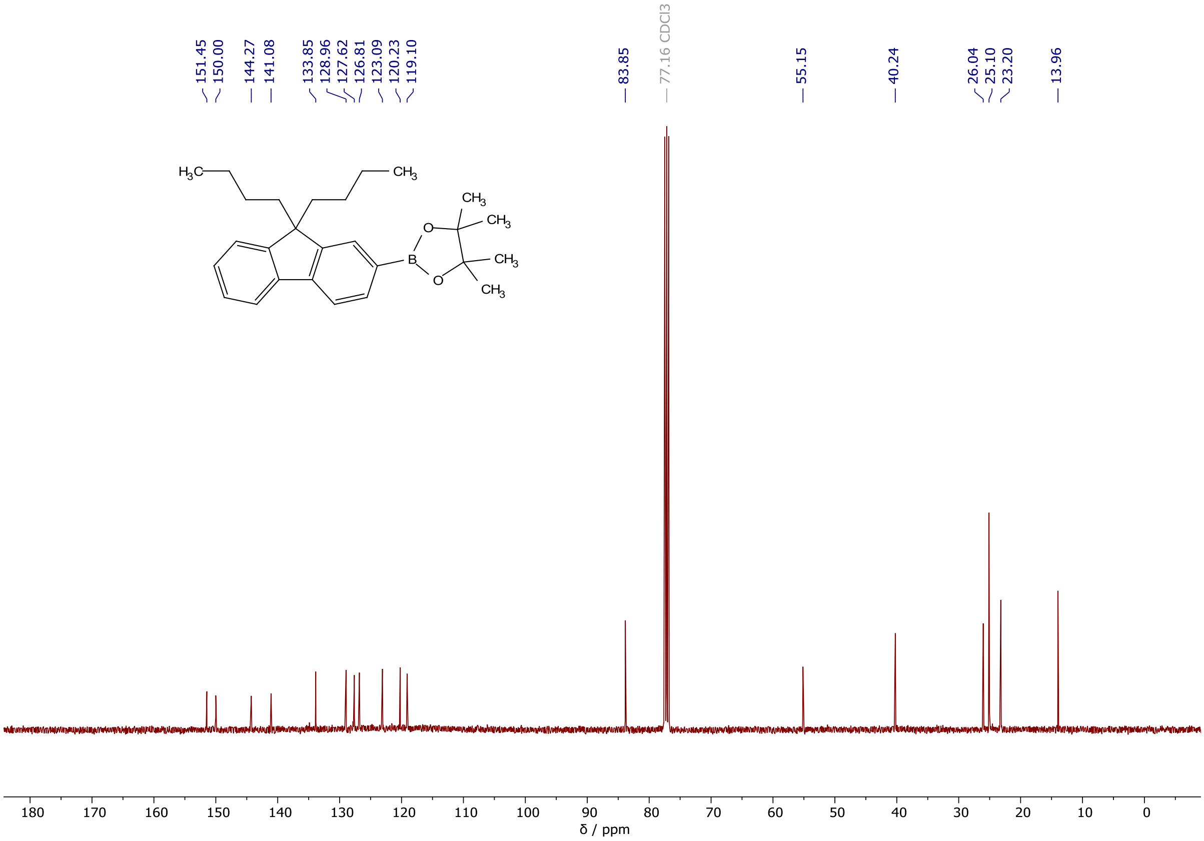

Figure S30: ^{13}C-NMR of 2-(9,9-dibutyl-9H-fluoren-2-yl)-4,4,5,5-tetramethyl-1,3,2-dioxaborolane in $CDCl_3$.

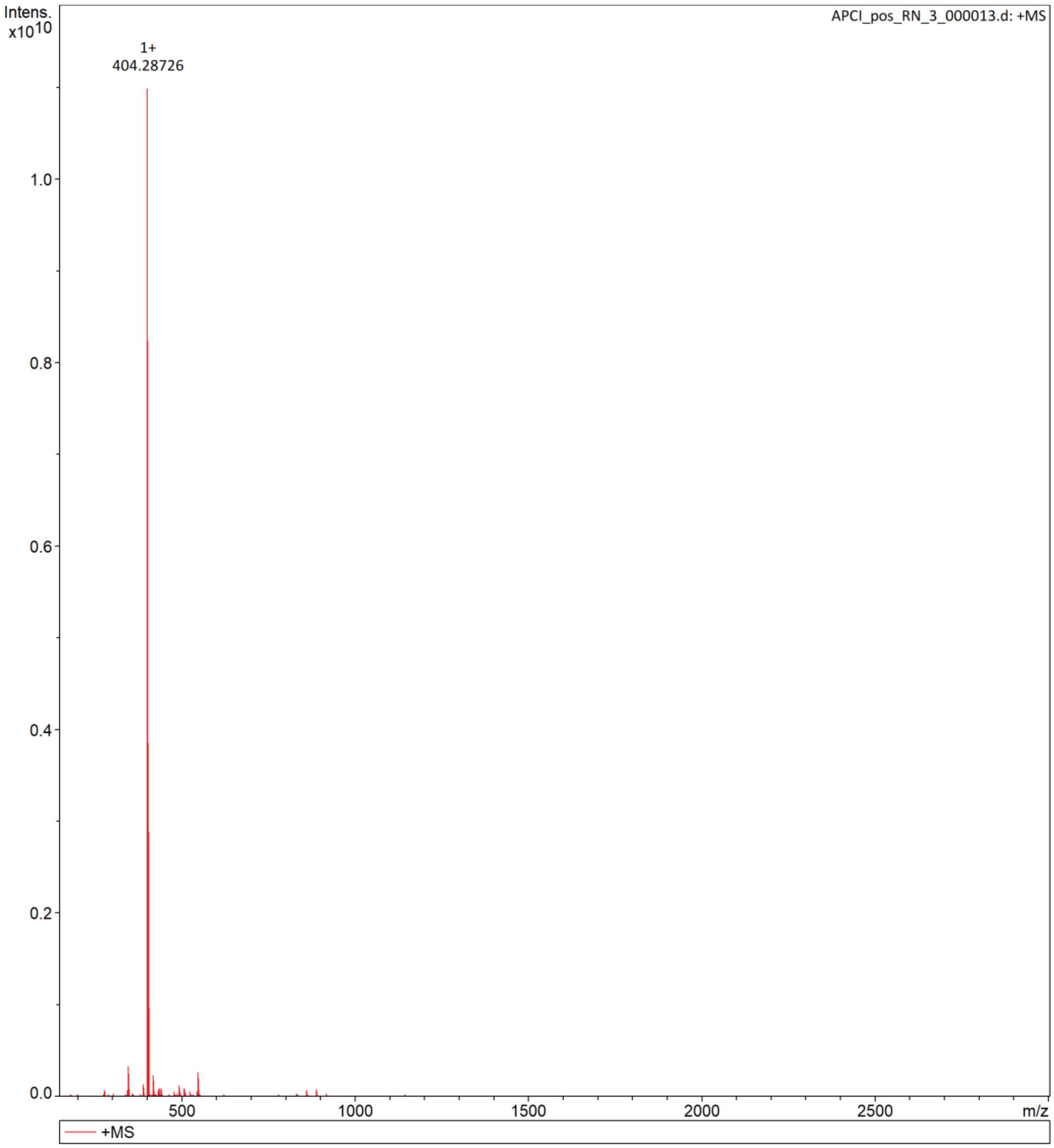

Figure S31: HR-APCI mass spectrum of 2-(9,9-dibutyl-9H-fluoren-2-yl)-4,4,5,5-tetramethyl-1,3,2-dioxaborolane.

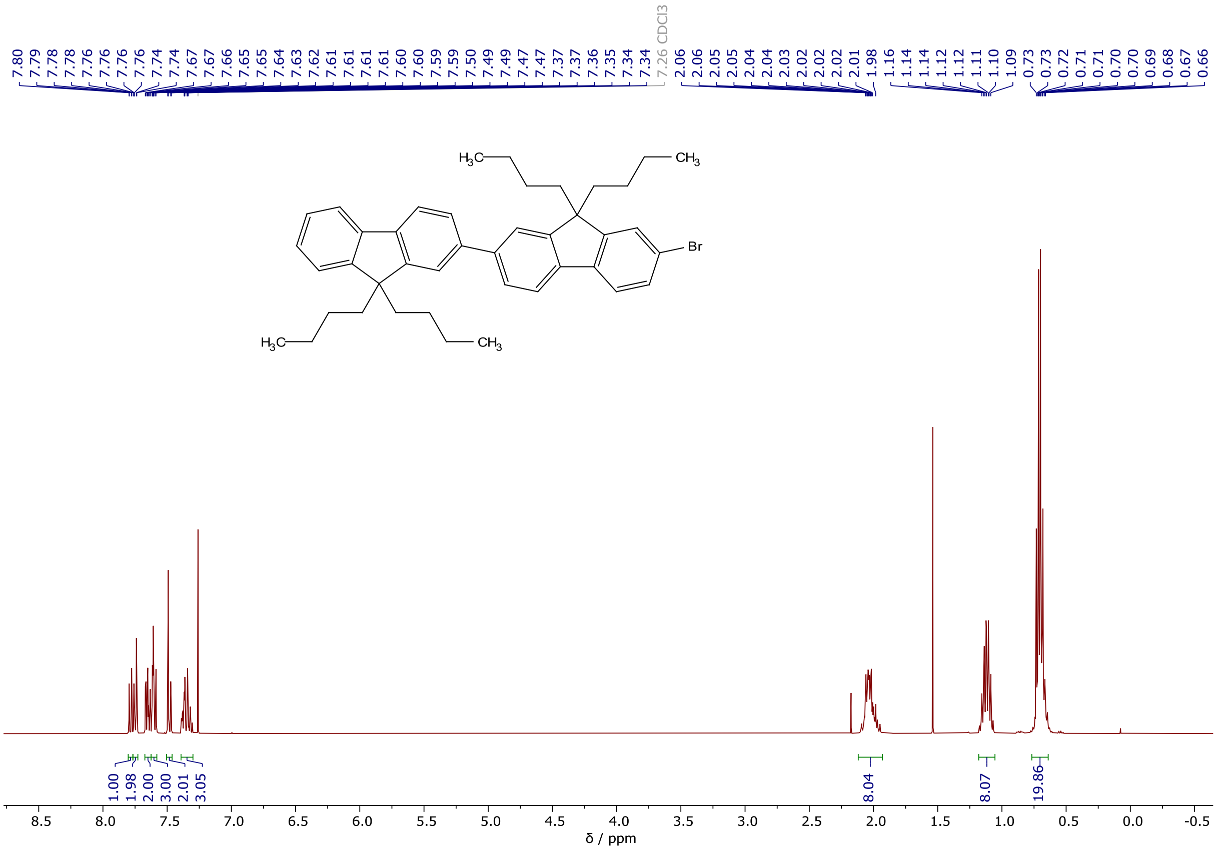

Figure S32: ^{1}H-NMR of 7-bromo-9,9,9',9'-tetrabutyl-9H,9H'-2,2'-bifluorene in $CDCl_3$.

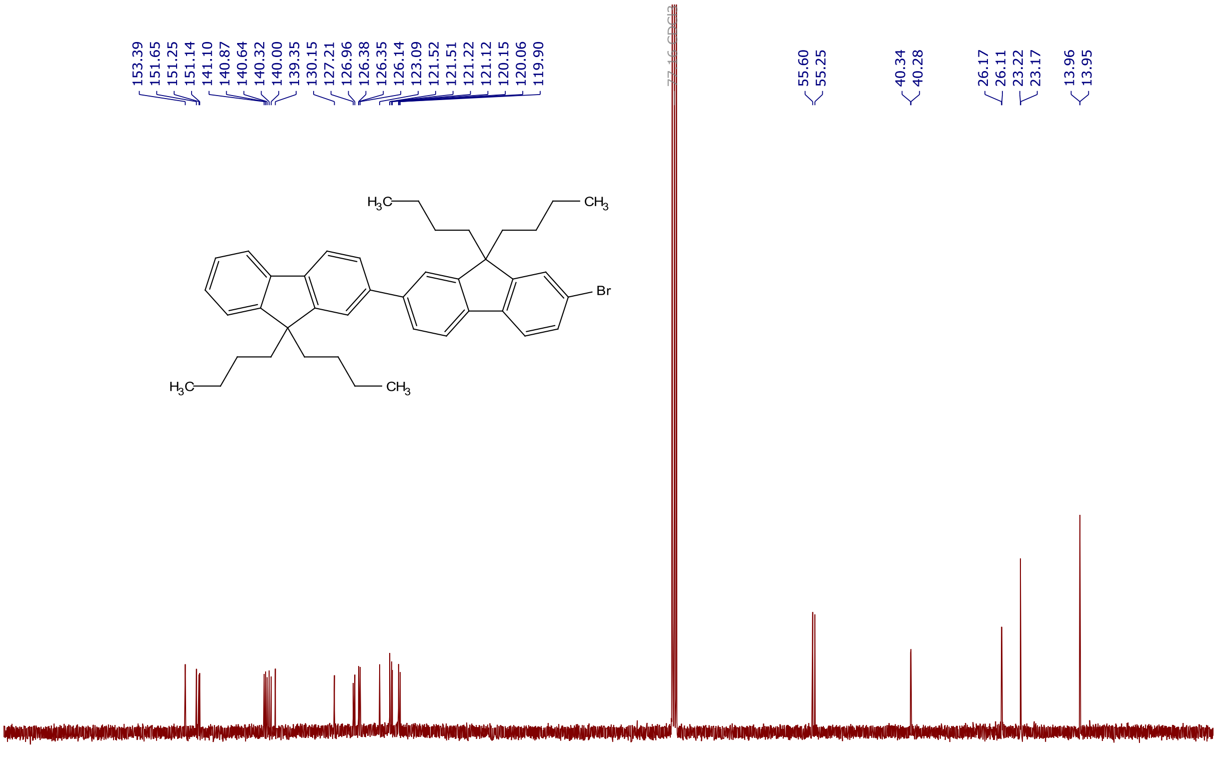

Figure S33: ^{13}C-NMR of 7-bromo-9,9,9',9'-tetrabutyl-9H,9H'-2,2'-bifluorene in $CDCl_3$.

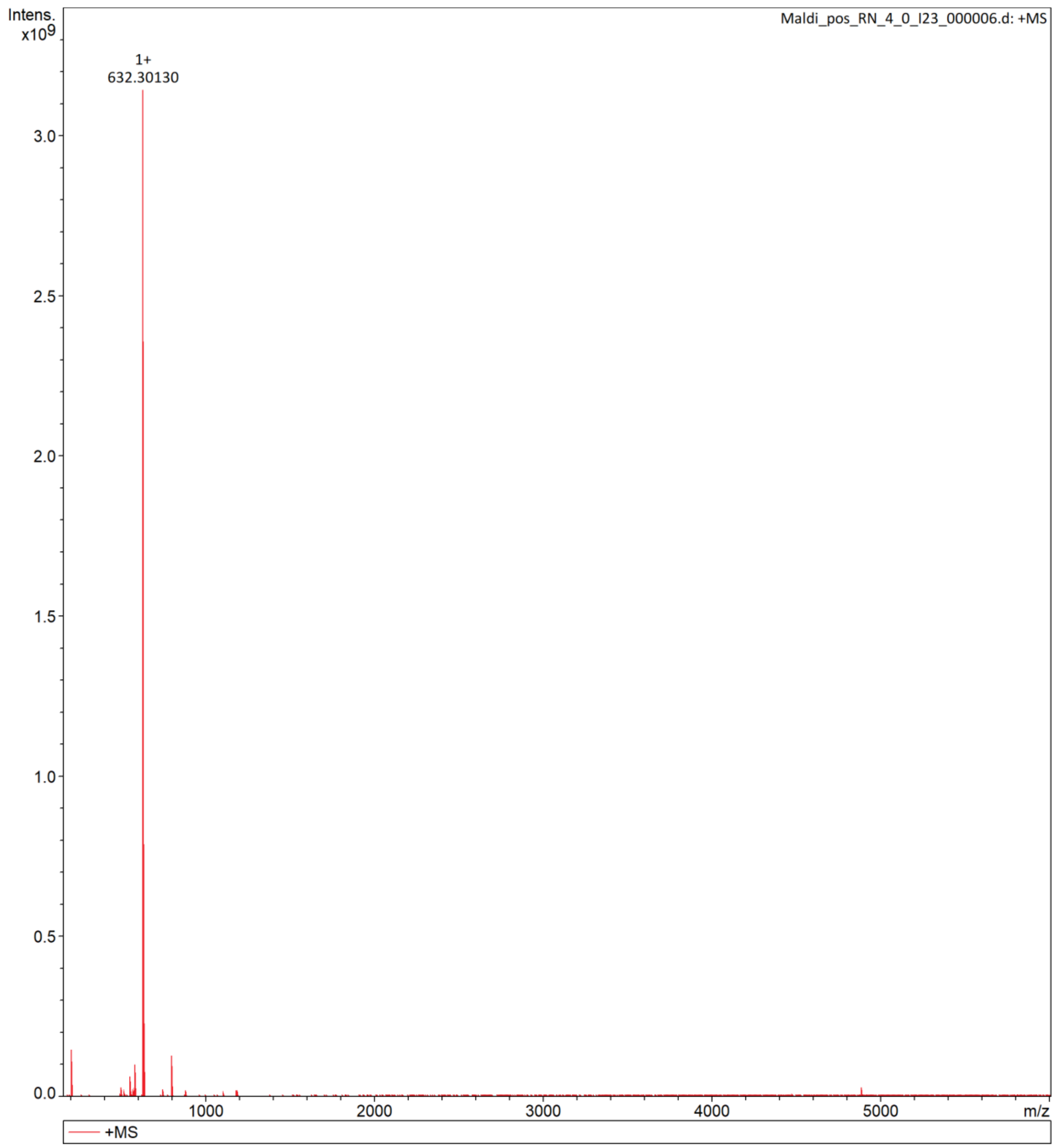

Figure S34: HR-MALDI mass spectrum of 7-bromo-9,9,9’,9’-tetrabutyl-9H,9H’-2,2’-bifluorene with DCTB as matrix.

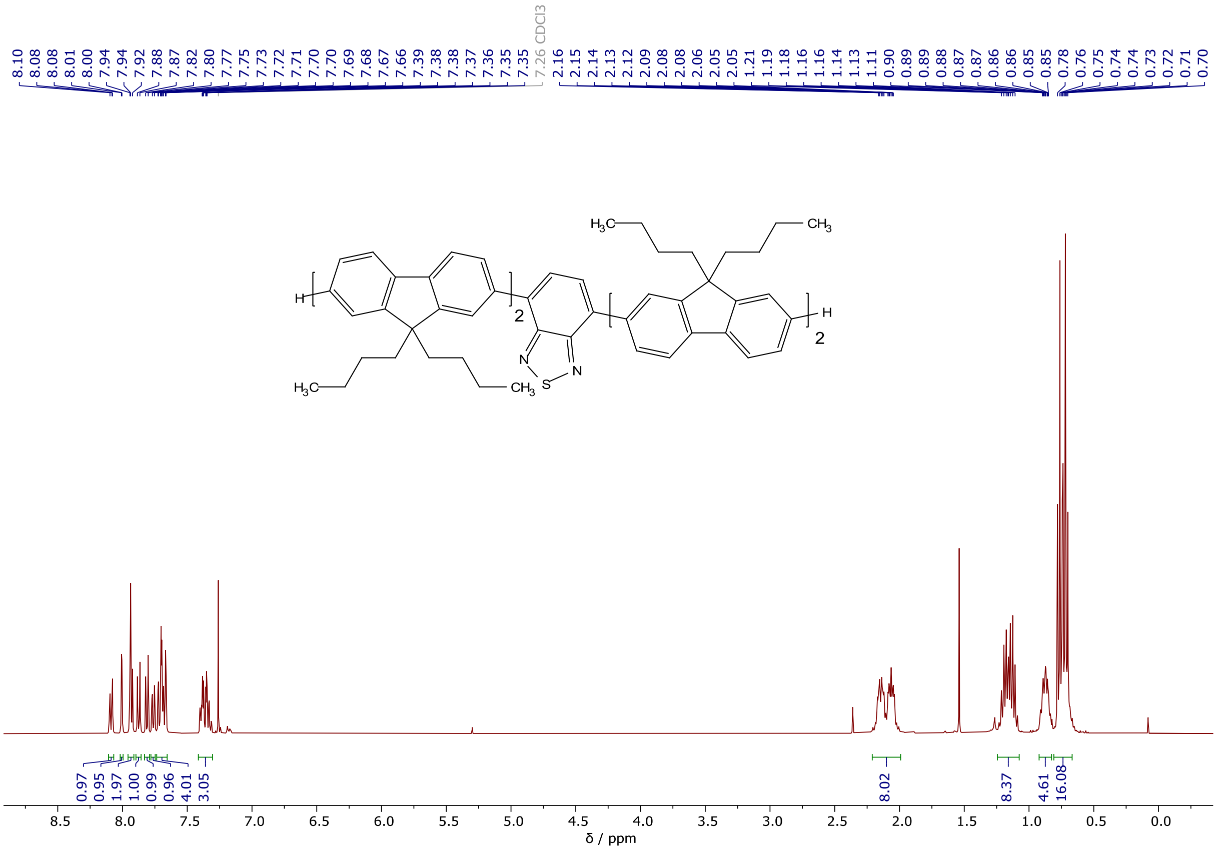

Figure S35: ^{1}H-NMR of 4,7-bis(9,9,9',9'-tetrabutyl-9H,9'H-[2,2'-bifluoren]-7-yl)benzo[c][1,2,5]thiadiazole (C4) in $CDCl_3$.

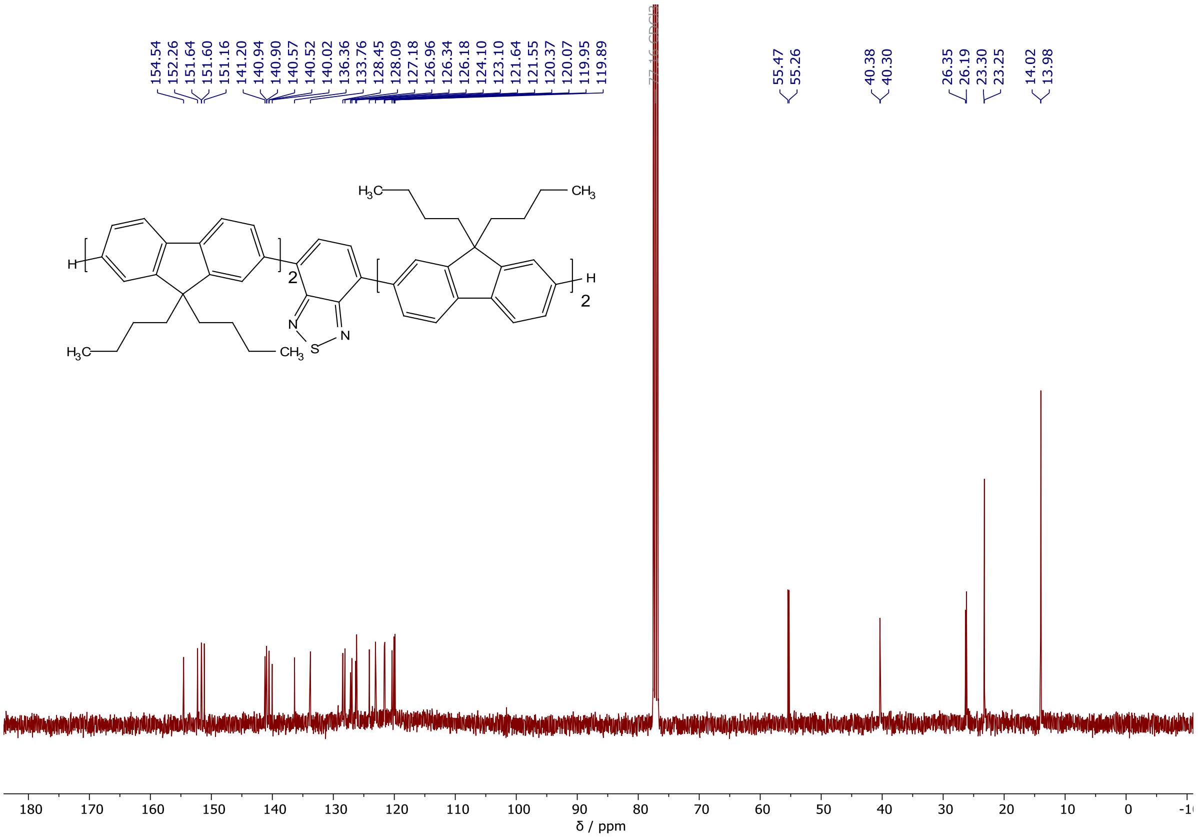

Figure S36: ^{13}C-NMR of 4,7-bis(9,9,9',9'-tetrabutyl-9H,9'H-[2,2'-bifluoren]-7-yl)benzo[c][1,2,5]thiadiazole (C4) in $CDCl_3$.

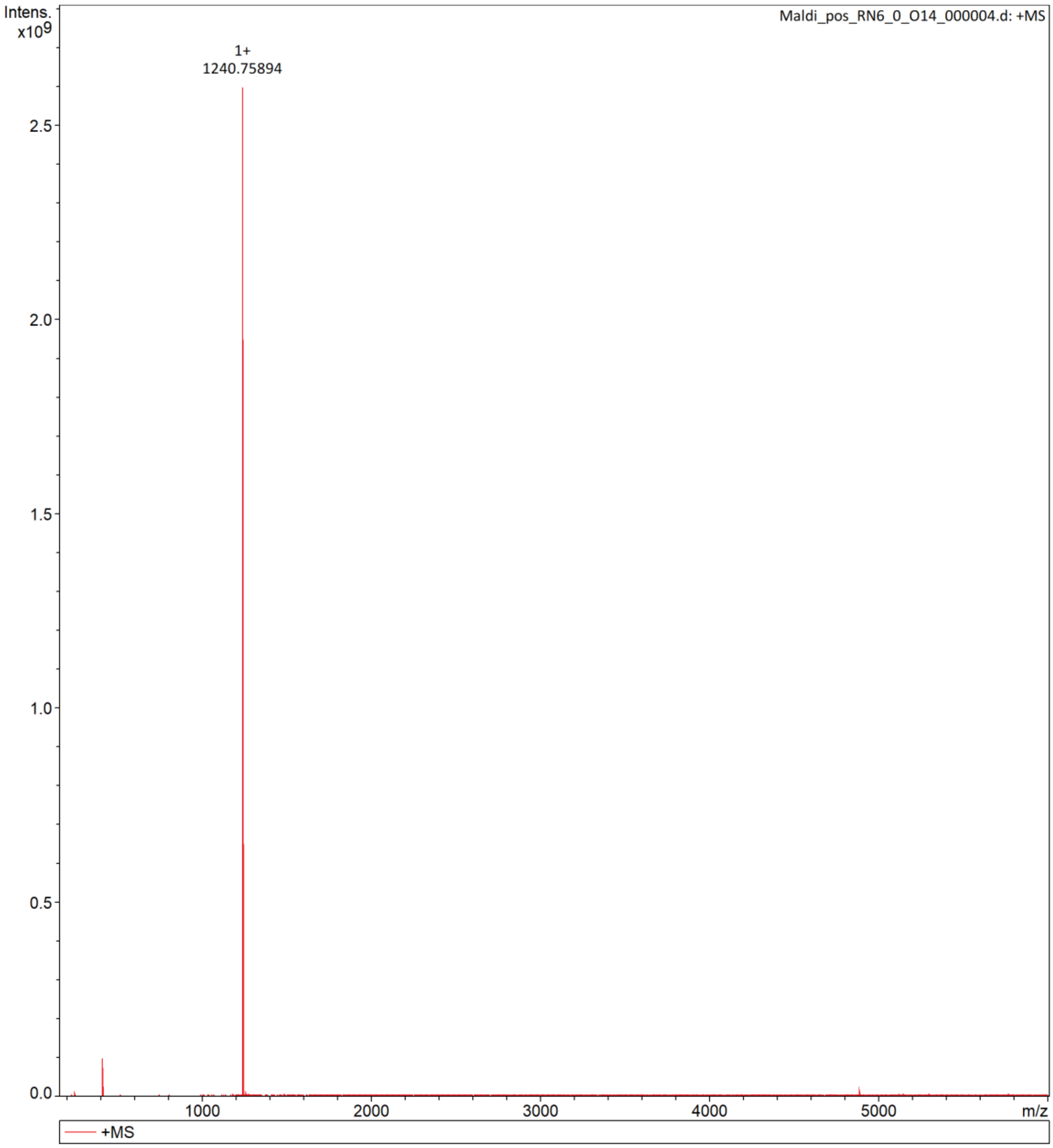

Figure S37: HR-MALDI mass spectrum of 4,7-bis(9,9,9',9'-tetrabutyl-9H,9'H-[2,2'-bifluoren]-7-yl)benzo[c][1,2,5]thiadiazole (C4) with DCTB as matrix.

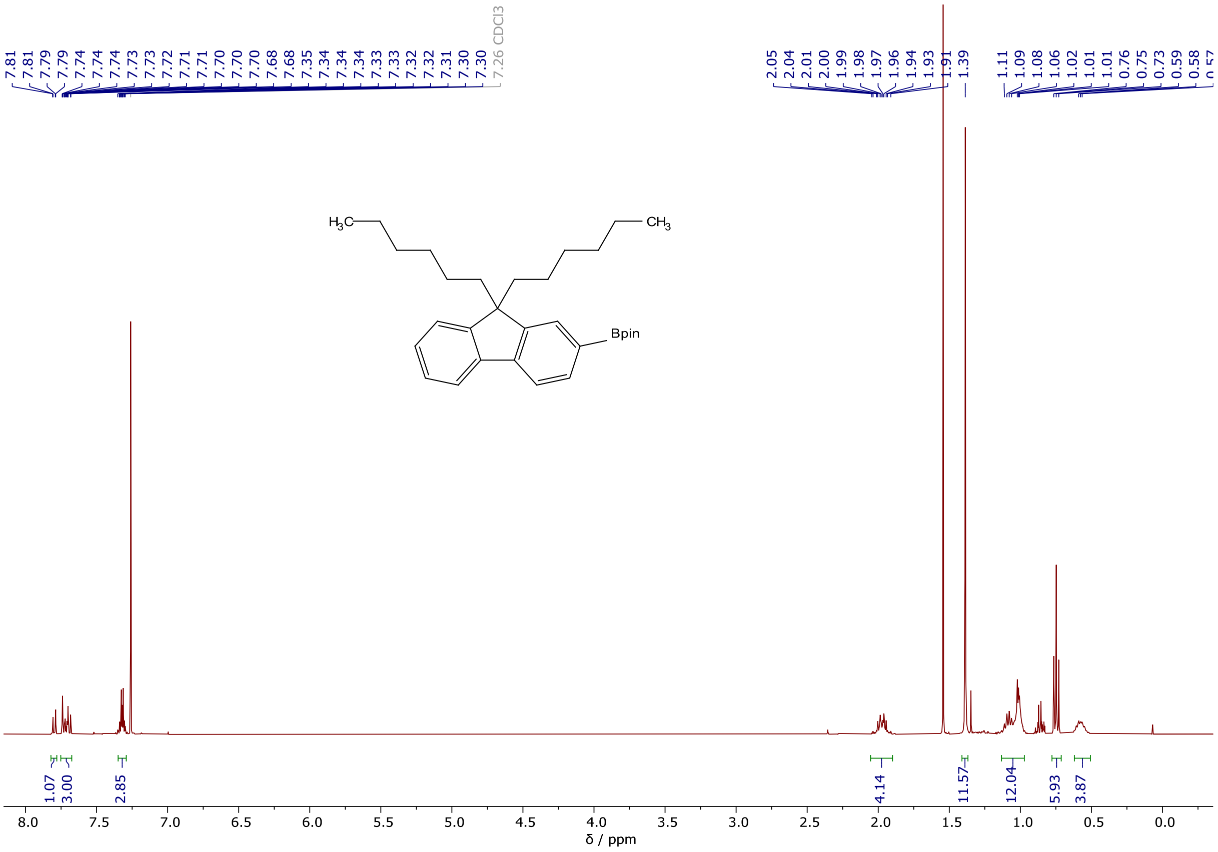

Figure S38: ^{1}H-NMR of 2-(9,9-dihexyl-9H-fluoren-2-yl)-4,4,5,5-tetramethyl-1,3,2-dioxaborolane in $CDCl_3$.

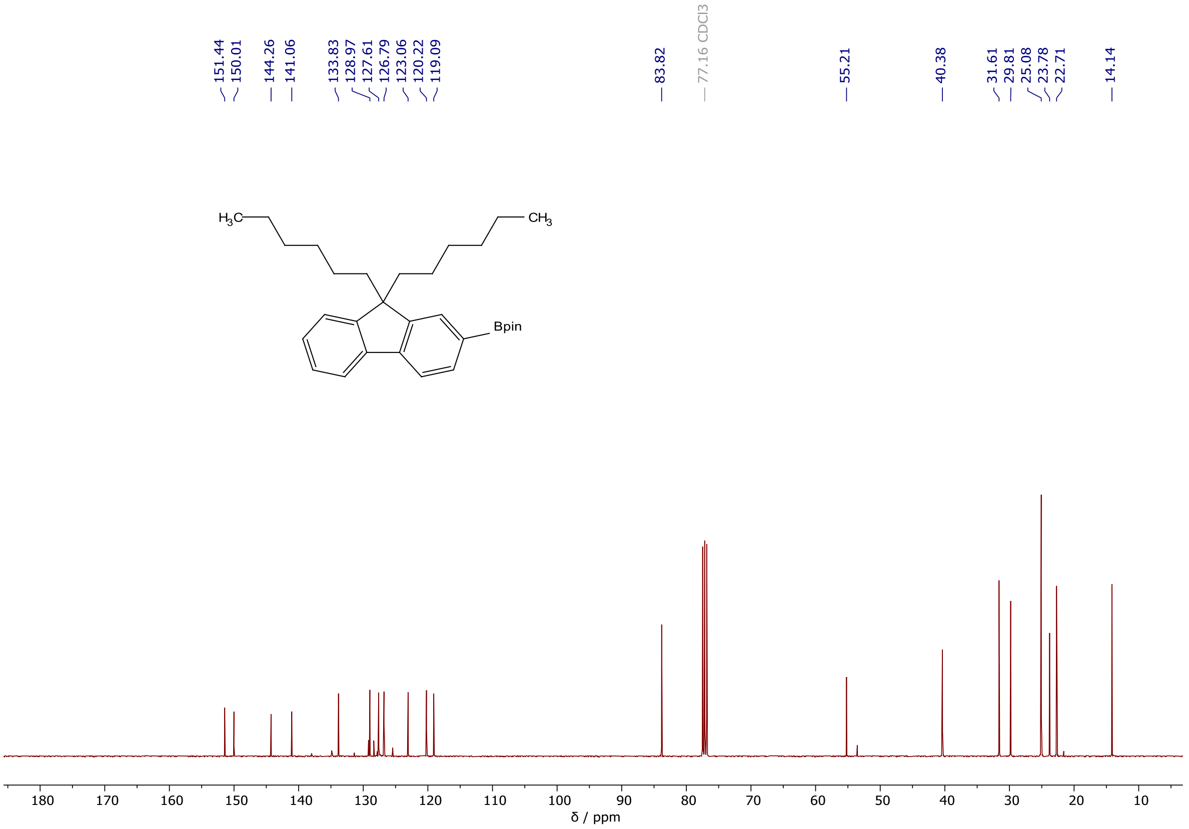

Figure S39: ^{13}C-NMR of 2-(9,9-dihexyl-9H-fluoren-2-yl)-4,4,5,5-tetramethyl-1,3,2-dioxaborolane in $CDCl_3$.

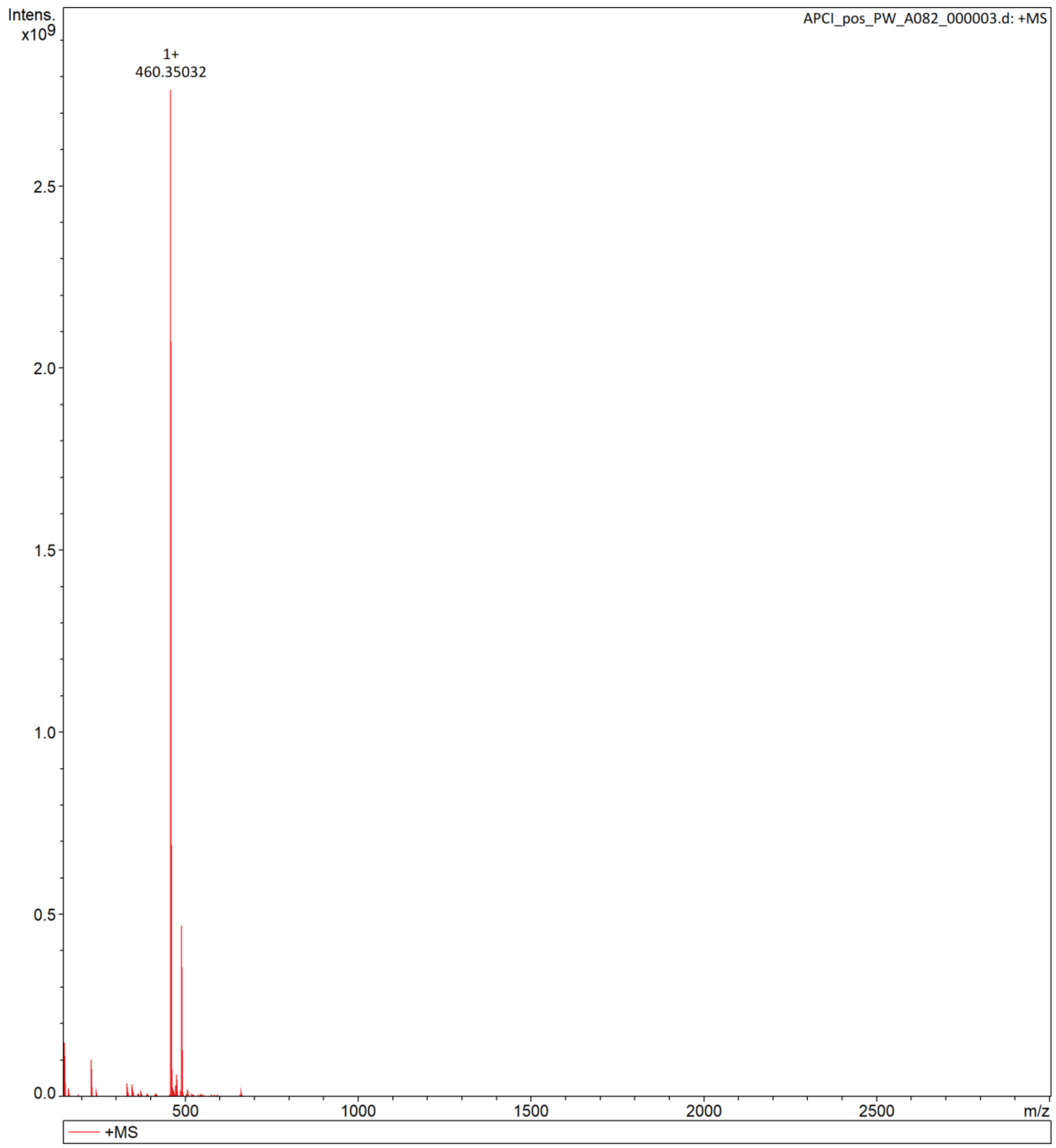

Figure S40: HR-APCI mass spectrum of 2-(9,9-dihexyl-9H-fluoren-2-yl)-4,4,5,5-tetramethyl-1,3,2-dioxaborolane.

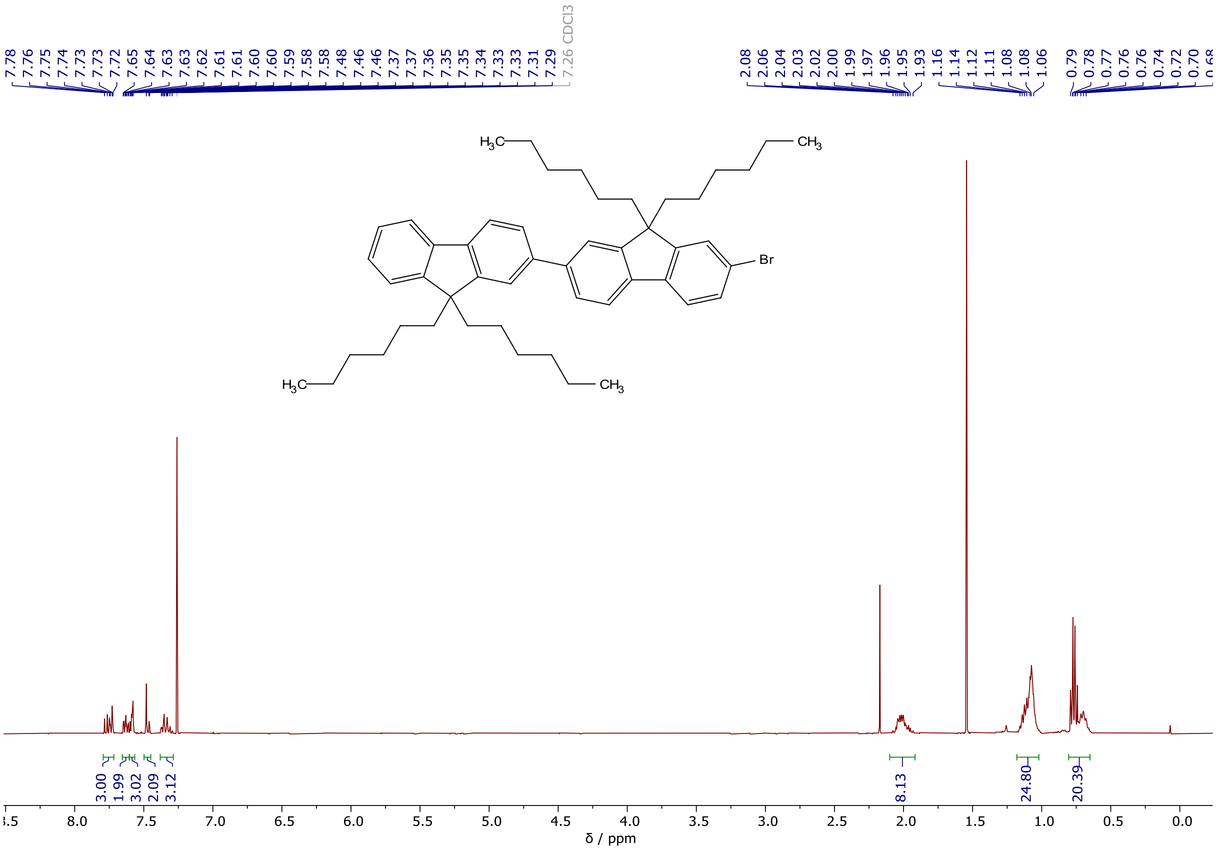

Figure S41: ^{1}H-NMR of 7-bromo-9,9,9',9'-tetrahexyl-9H,9H'-2,2'-bifluorene in $CDCl_3$.

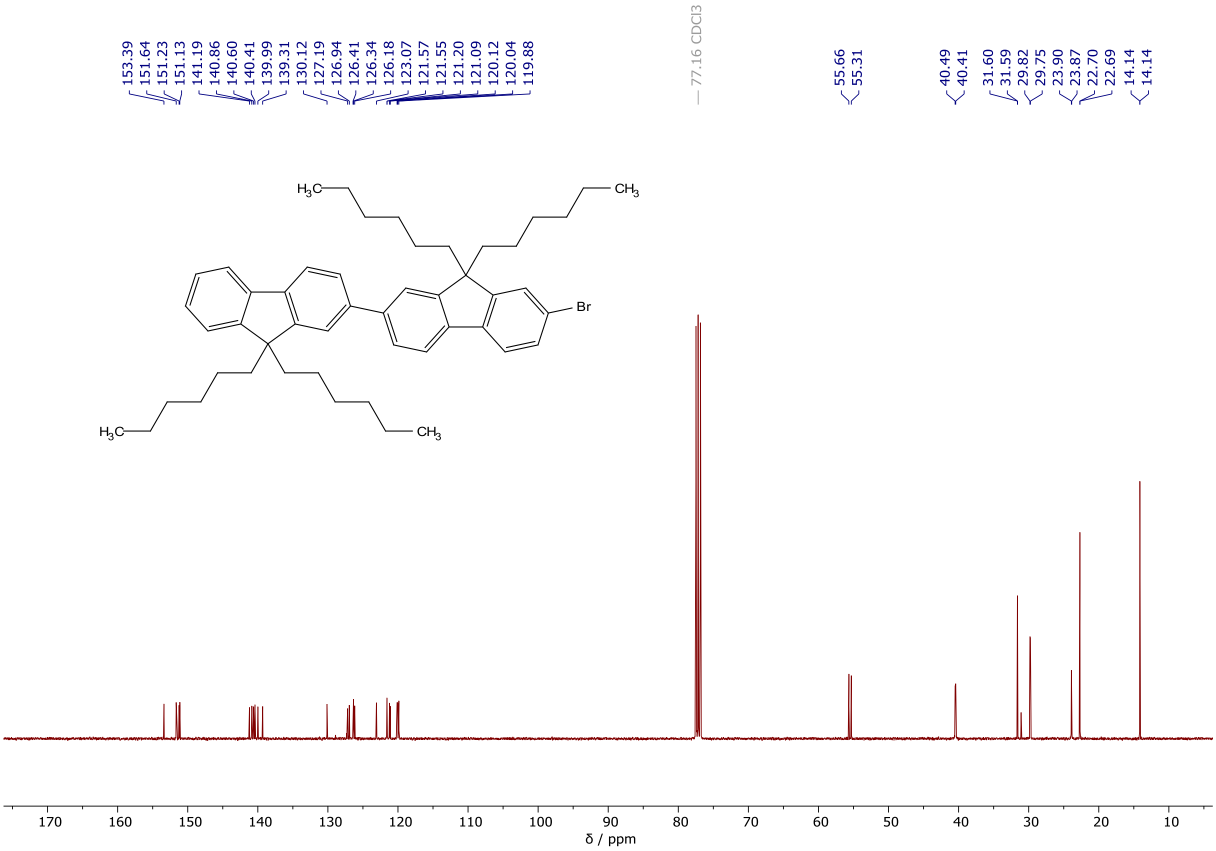

Figure S42: ^{13}C-NMR of 7-bromo-9,9,9',9'-tetrahexyl-9H,9H'-2,2'-bifluorene in $CDCl_3$.

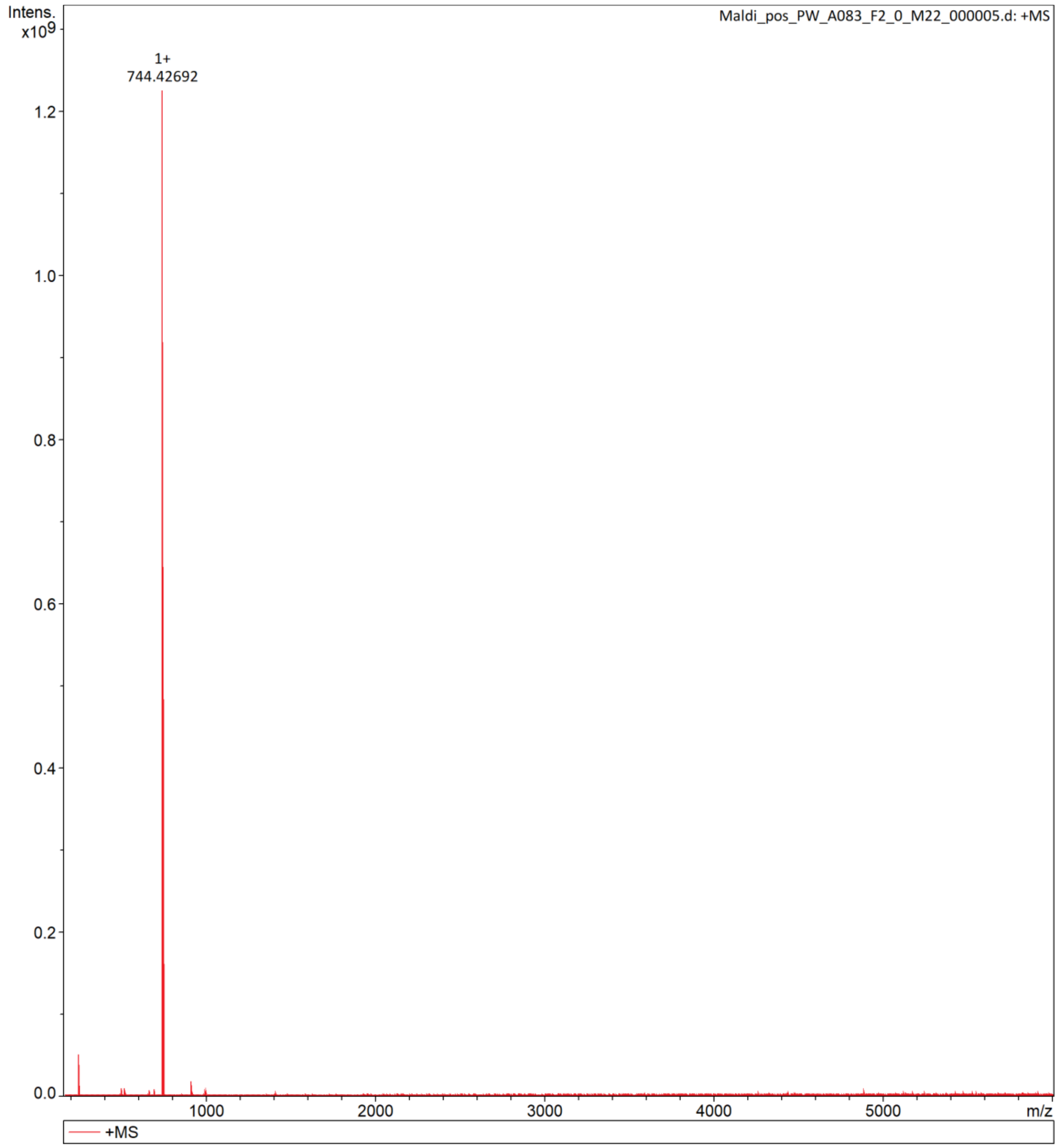

Figure S43: HR-MALDI mass spectrum of 7-bromo-9,9,9',9'-tetrahexyl-9H,9H'-2,2'-bifluorene with DCTB as matrix.

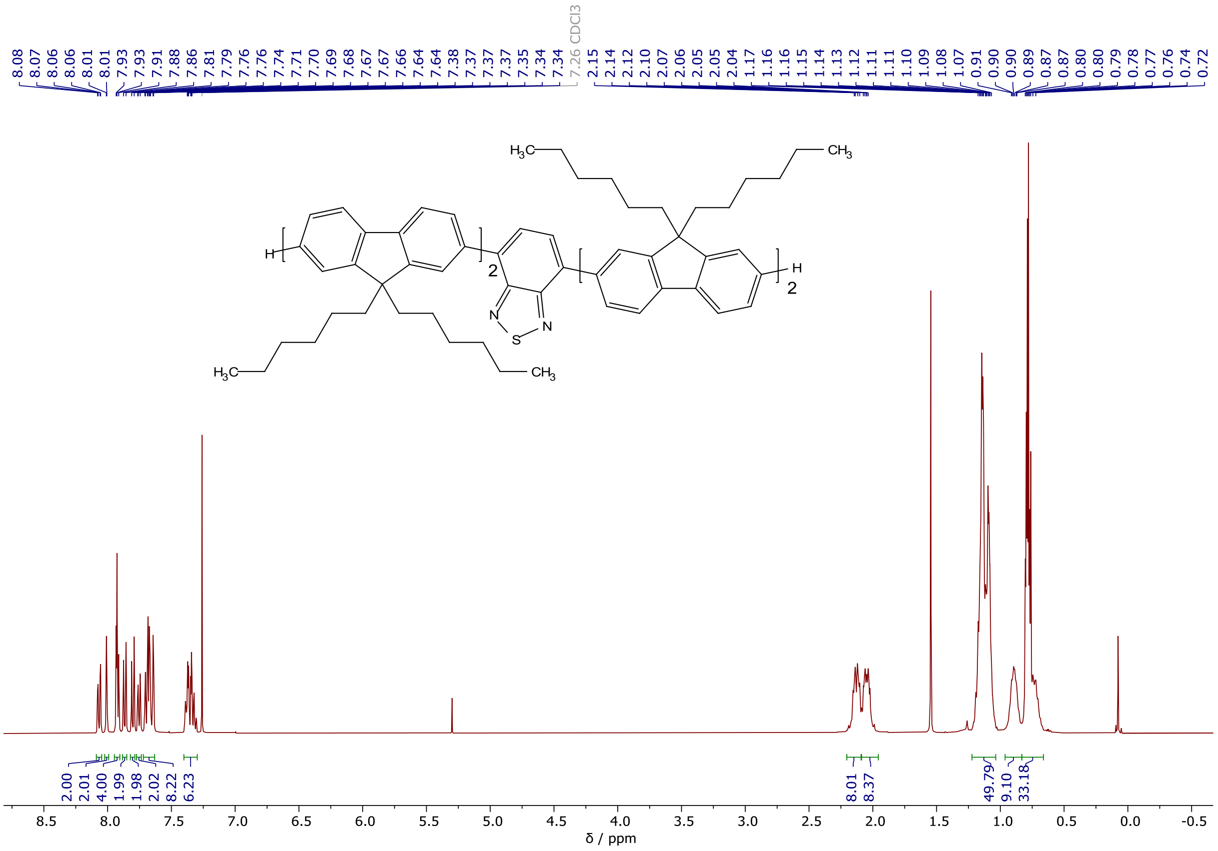

Figure S44: ^{1}H-NMR of 4,7-bis(9,9,9',9'-tetrahexyl-9H,9'H-[2,2'-bifluoren]-7-yl)benzo[c][1,2,5]thia-diazole (C6) in $CDCl_3$.

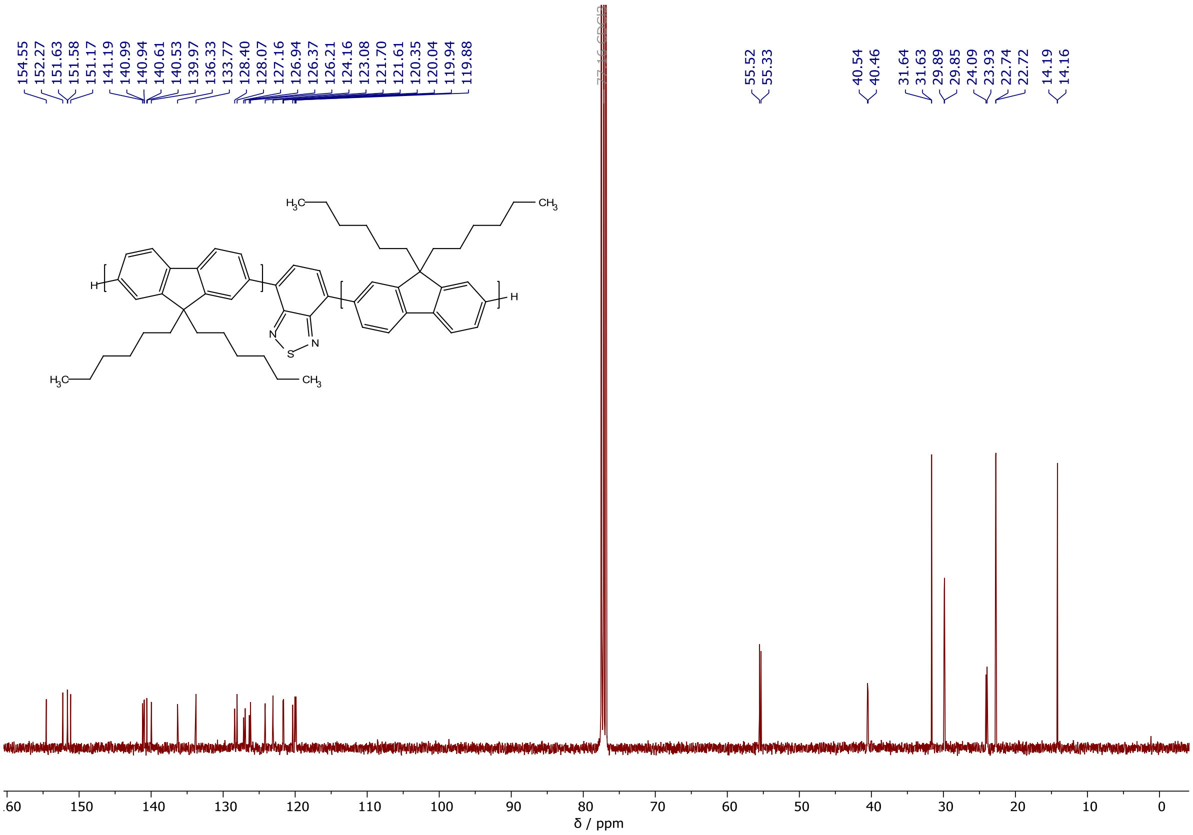

Figure S45: ^{13}C-NMR of 4,7-bis(9,9,9',9'-tetrahexyl-9H,9'H-[2,2'-bifluoren]-7-yl)benzo[c][1,2,5]thia-diazole (C6) in $CDCl_3$.

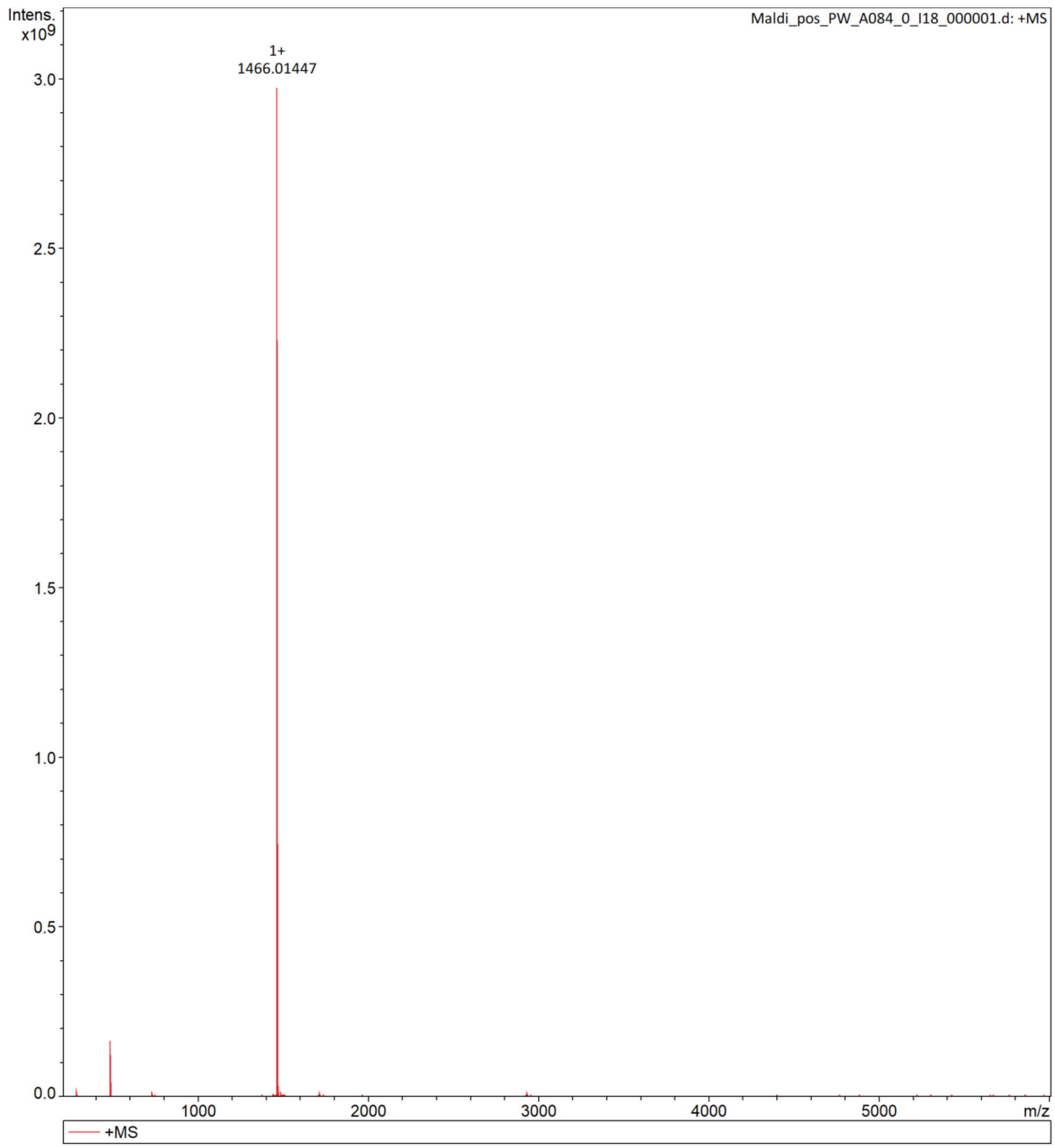

Figure S46: HR-MALDI mass spectrum of 4,7-bis(9,9,9',9'-tetrahexyl-9H,9'H-[2,2'-bifluoren]-7-yl)benzo[c][1,2,5]thiadiazole (C6) with DCTB as matrix.

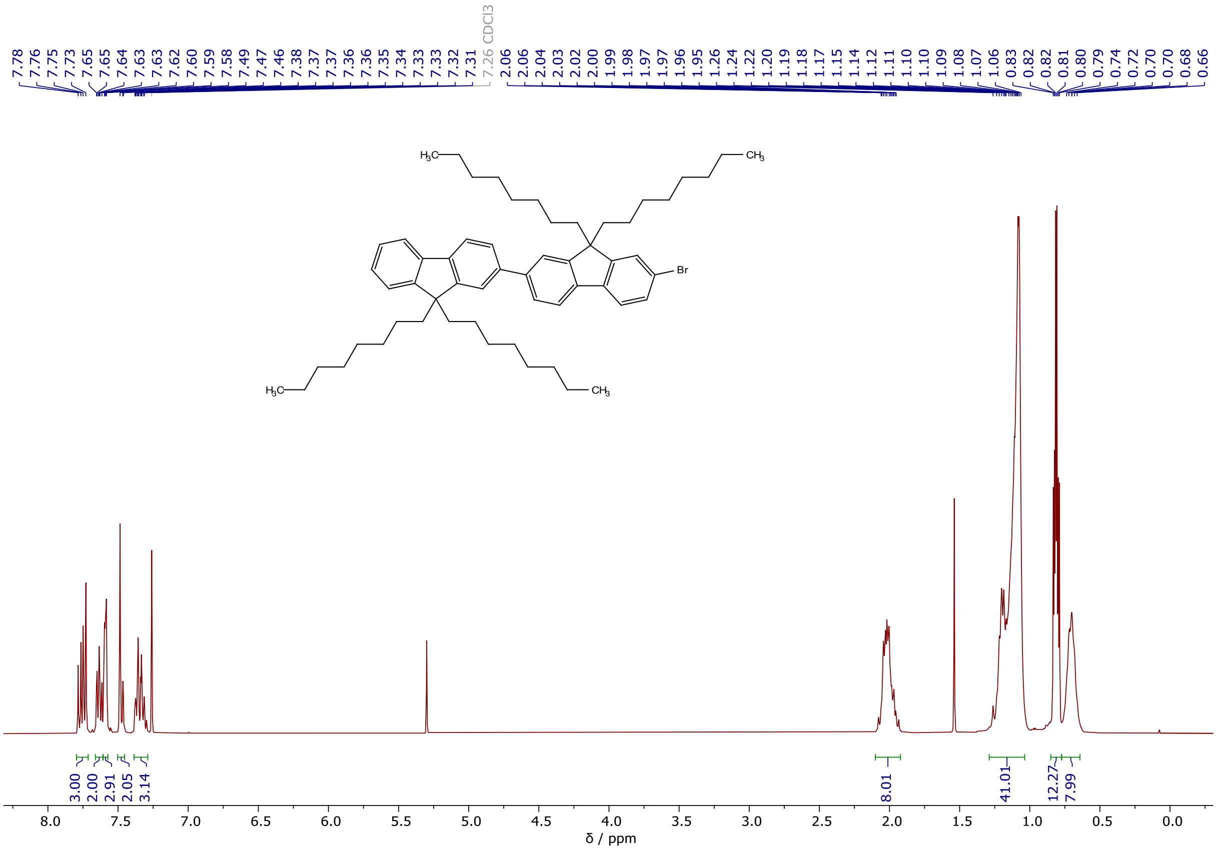

Figure S47: ^{1}H-NMR of 7-bromo-9,9,9’,9’-tetraoctyl-9H,9H’-2,2’-bifluorene in $CDCl_3$.

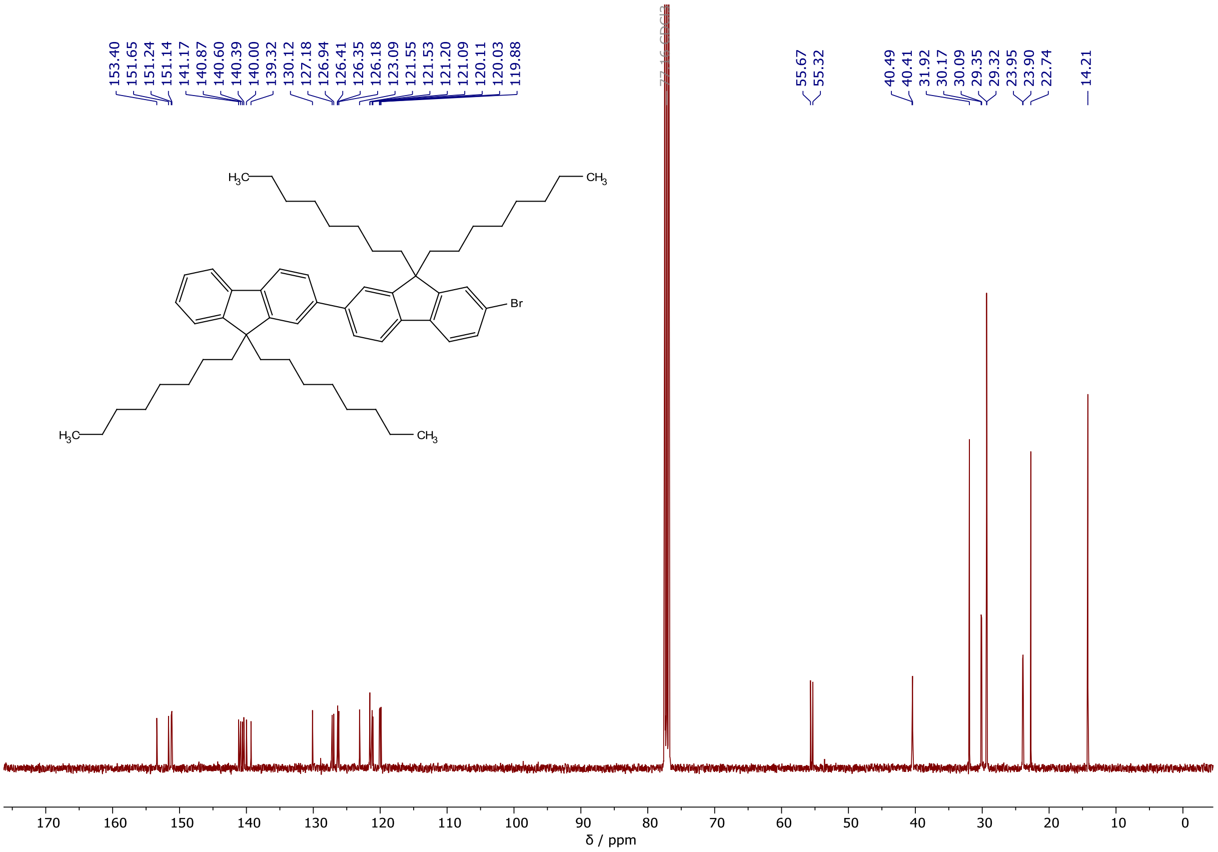

Figure S48: ^{13}C-NMR of 7-bromo-9,9,9’,9’-tetraoctyl-9H,9H’-2,2’-bifluorene in $CDCl_3$.

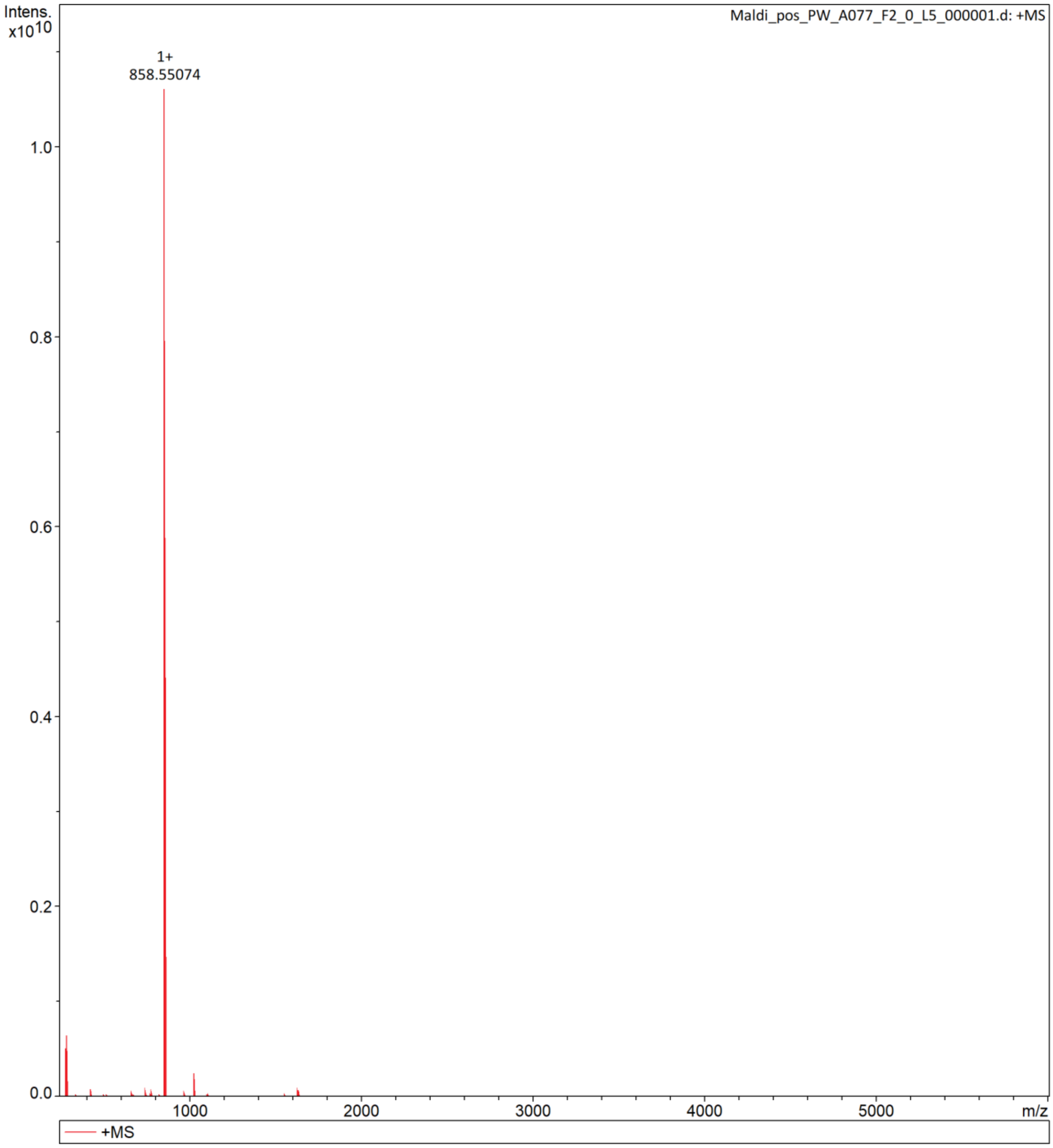

Figure S49: HR-MALDI mass spectrum of 7-bromo-9,9,9',9'-tetraoctyl-9H,9H'-2,2'-bifluorene with DCTB as matrix.

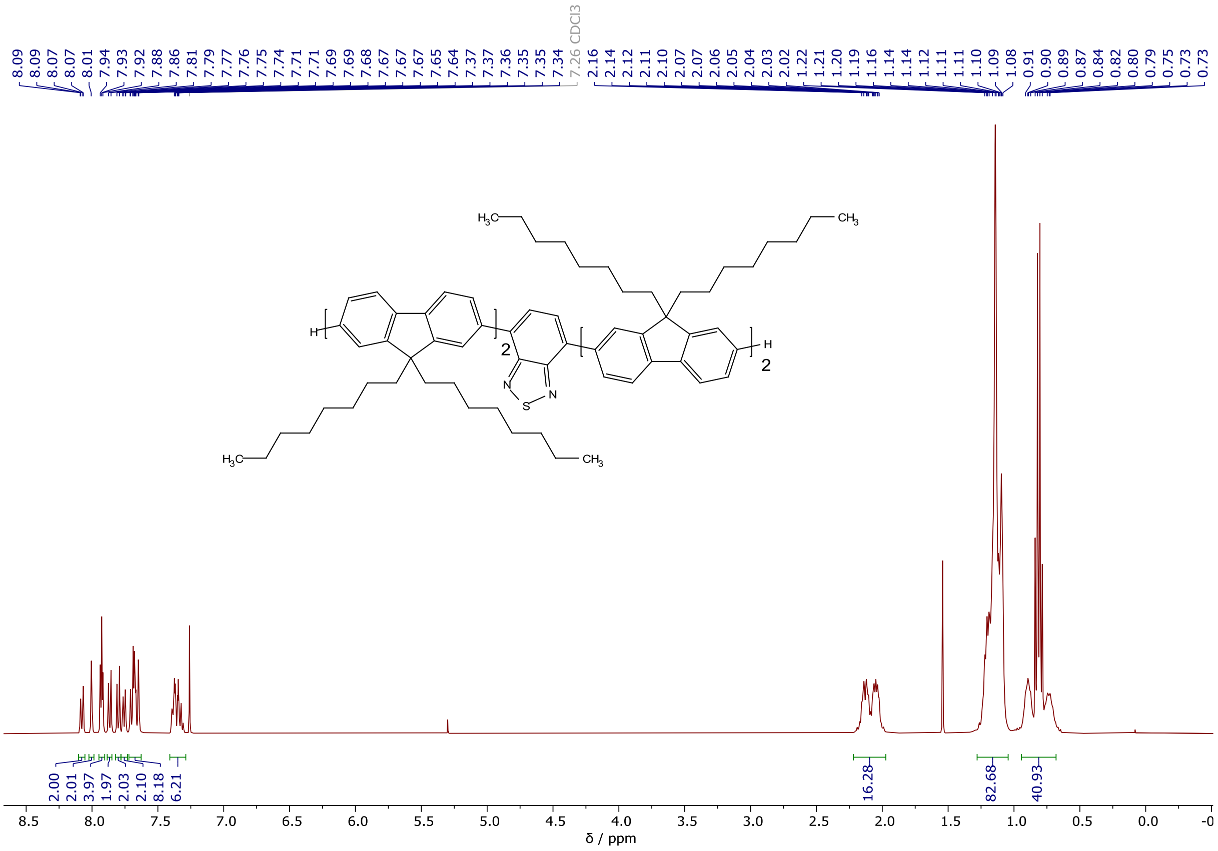

Figure S50: ^{1}H-NMR of 4,7-bis(9,9,9',9'-tetraoctyl-9H,9'H-[2,2'-bifluoren]-7-yl)benzo[c][1,2,5]thiadiazole (C8) in $CDCl_3$.

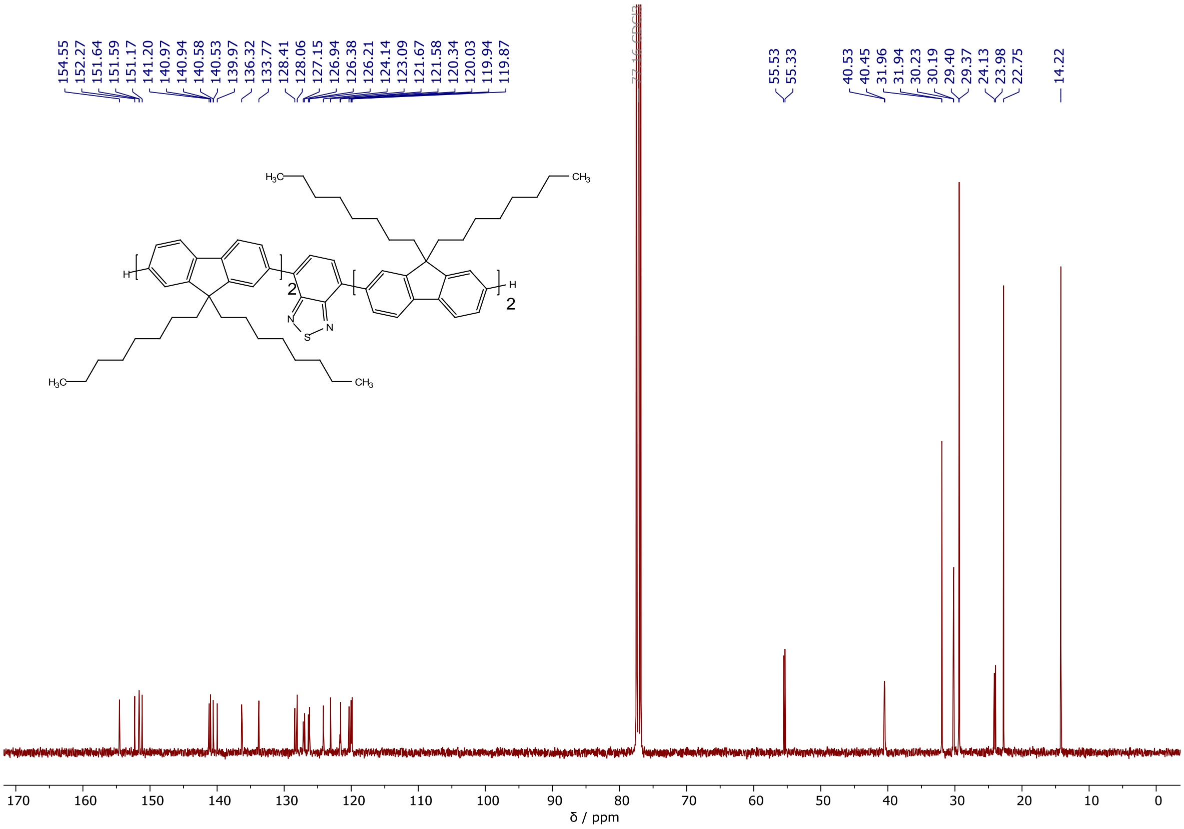

Figure S51: ^{13}C-NMR of 4,7-bis(9,9,9',9'-tetraoctyl-9H,9'H-[2,2'-bifluoren]-7-yl)benzo[c][1,2,5]thiadiazole (C8) in $CDCl_3$.

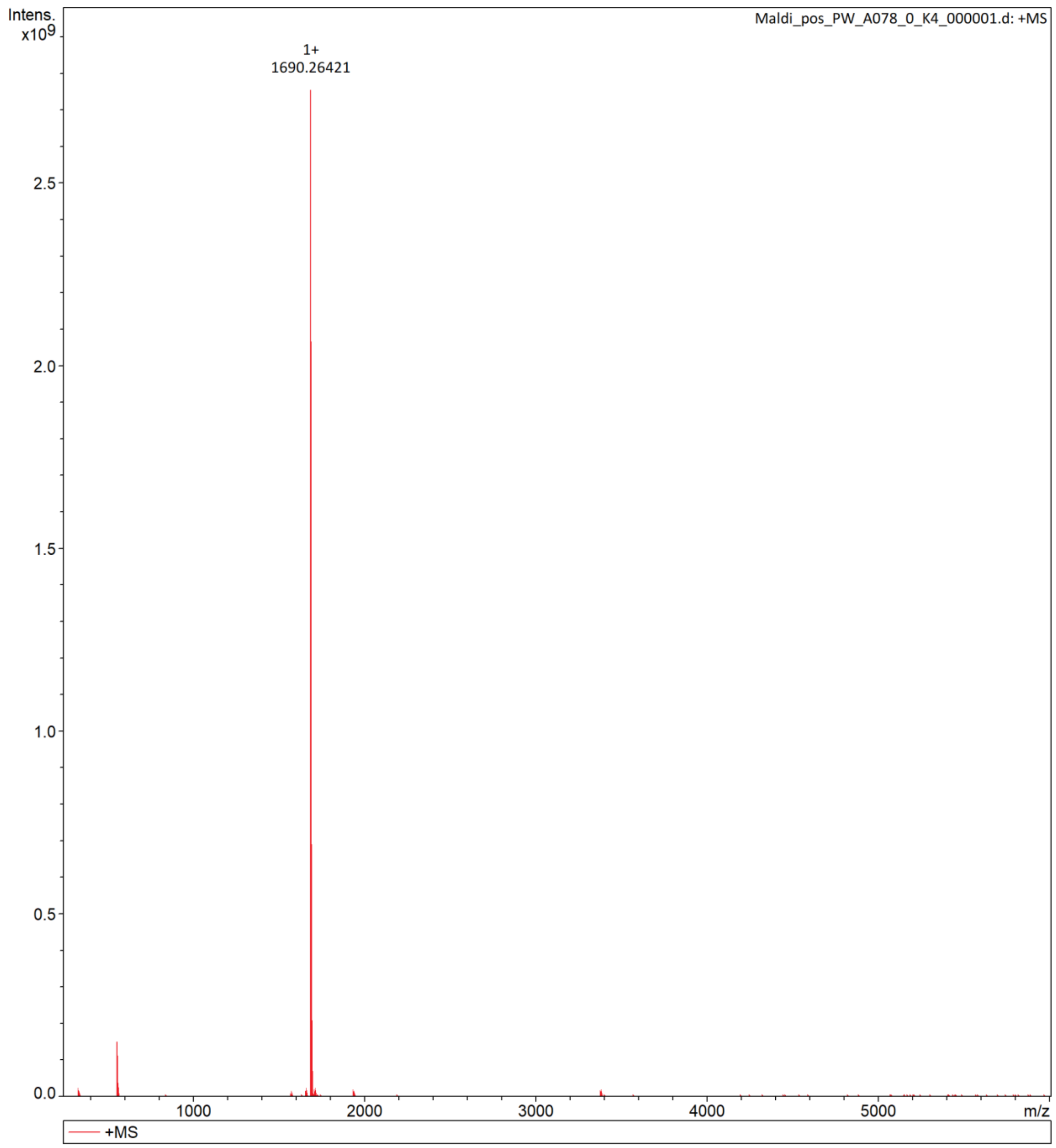

Figure S52: HR-MALDI mass spectrum of 4,7-bis(9,9,9',9'-tetraoctyl-9H,9'H-[2,2'-bifluoren]-7-yl)benzo[c][1,2,5]thiadiazole (C8) with DCTB as matrix.

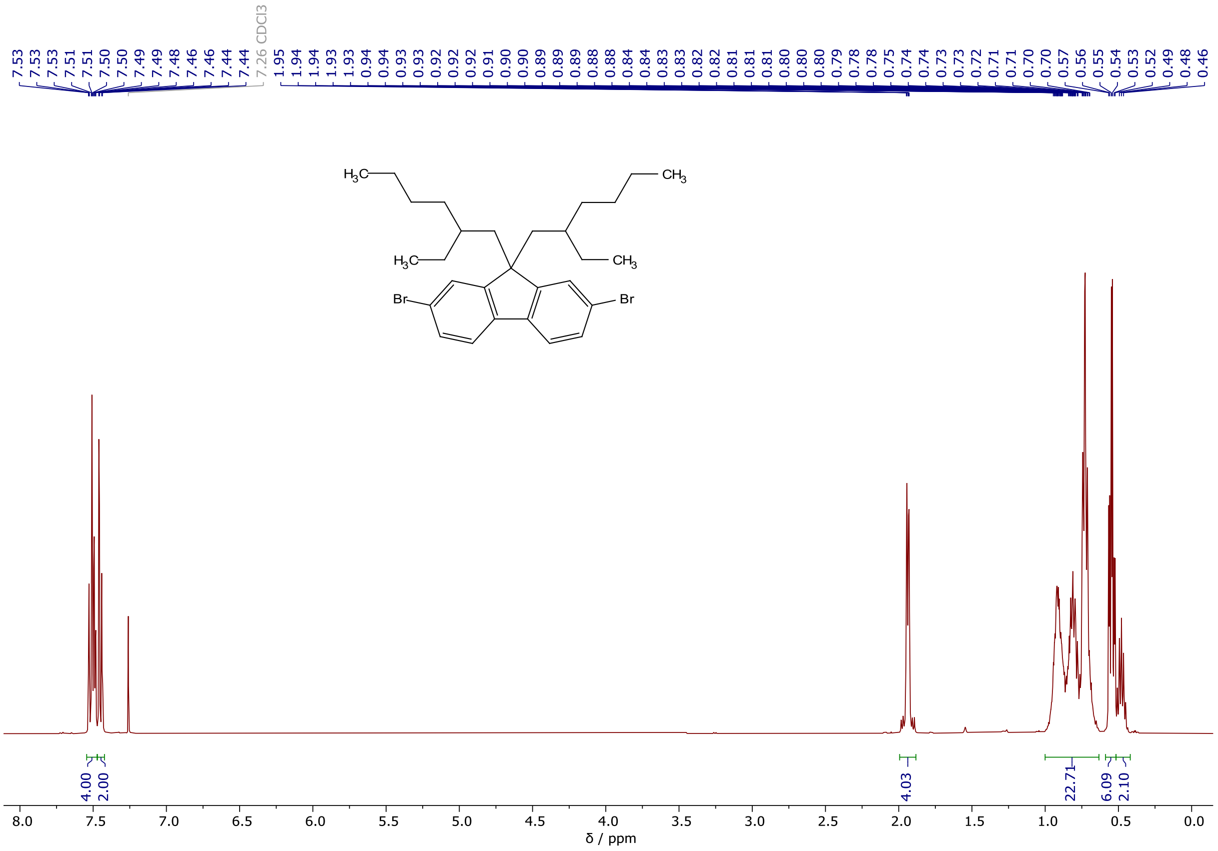

Figure S53: ^{1}H-NMR of 2,7-dibromo-9,9-bis(2-ethylhexyl)-9H-fluorene in $CDCl_3$.

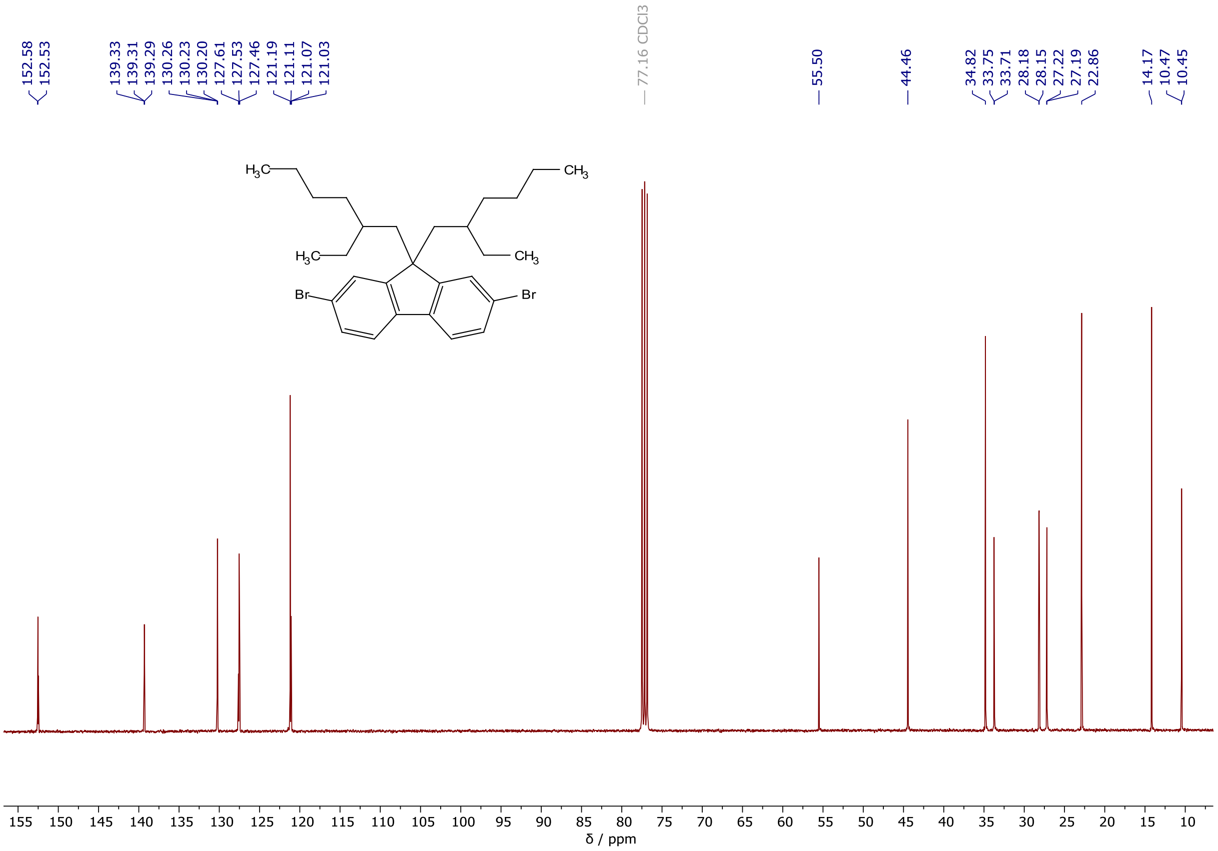

Figure S54: ^{13}C-NMR of 2,7-dibromo-9,9-bis(2-ethylhexyl)-9H-fluorene in $CDCl_3$.

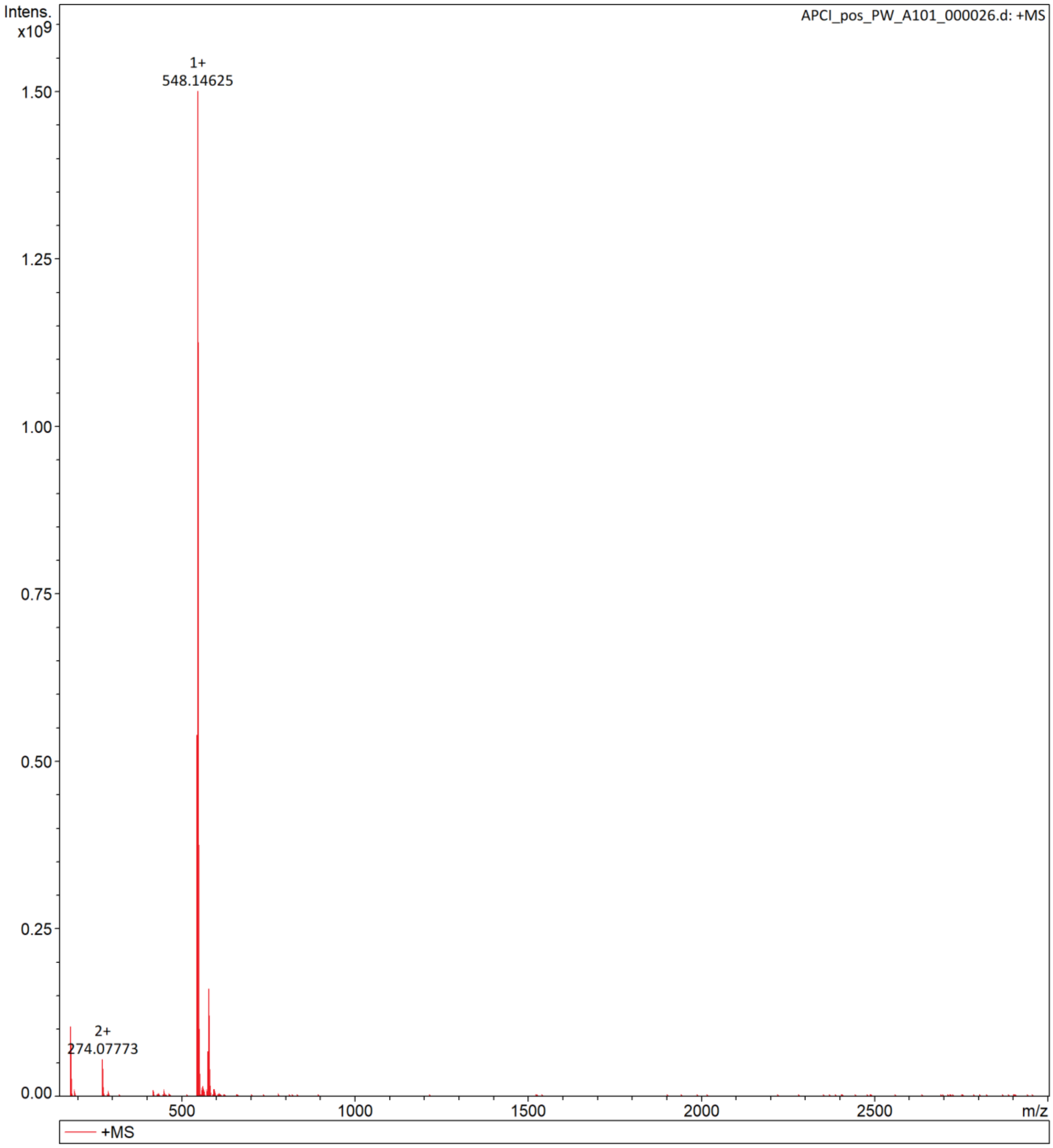

Figure S55: HR-APCI mass spectrum of 2,7-dibromo-9,9-bis(2-ethylhexyl)-9H-fluorene.

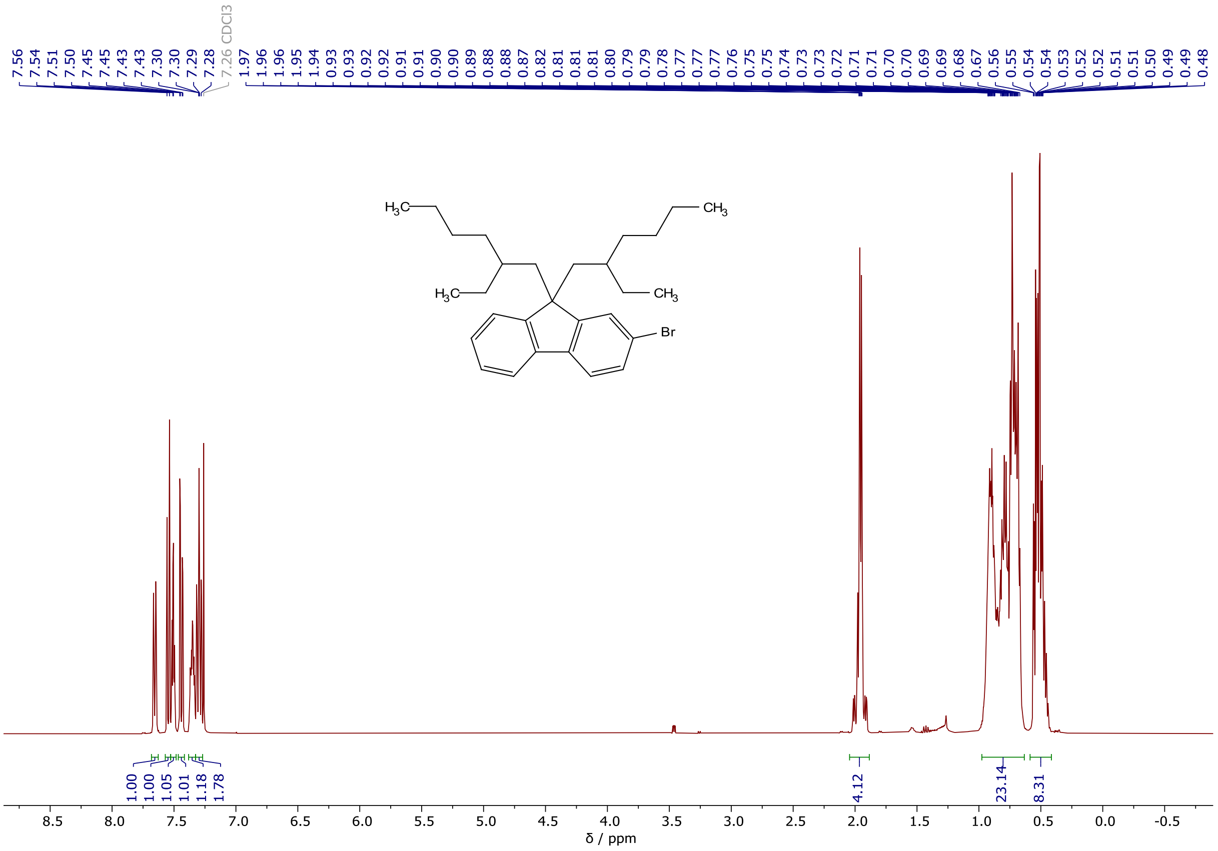

Figure S56: ^{1}H-NMR of 2-bromo-9,9-bis(2-ethylhexyl)-9H-fluorene in $CDCl_3$.

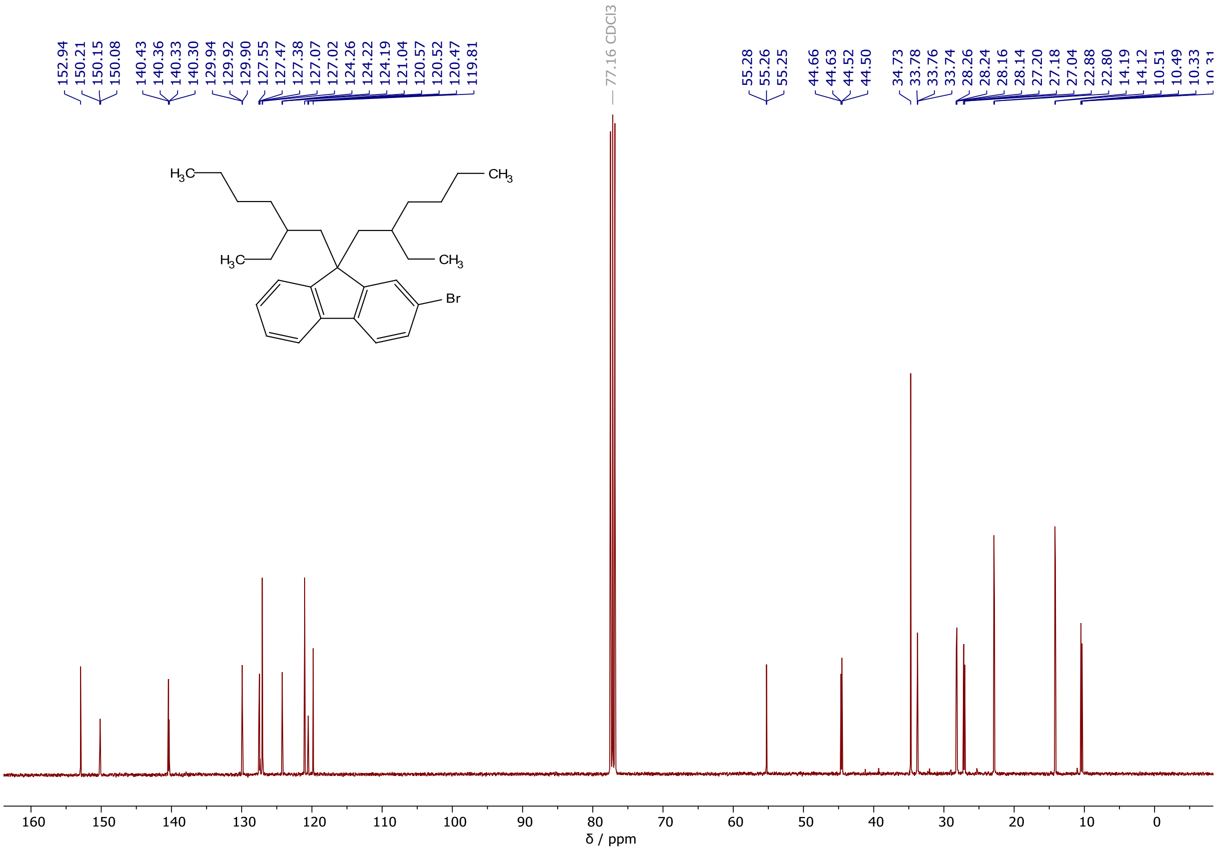

Figure S57: ^{13}C-NMR of 2-bromo-9,9-bis(2-ethylhexyl)-9H-fluorene in $CDCl_3$.

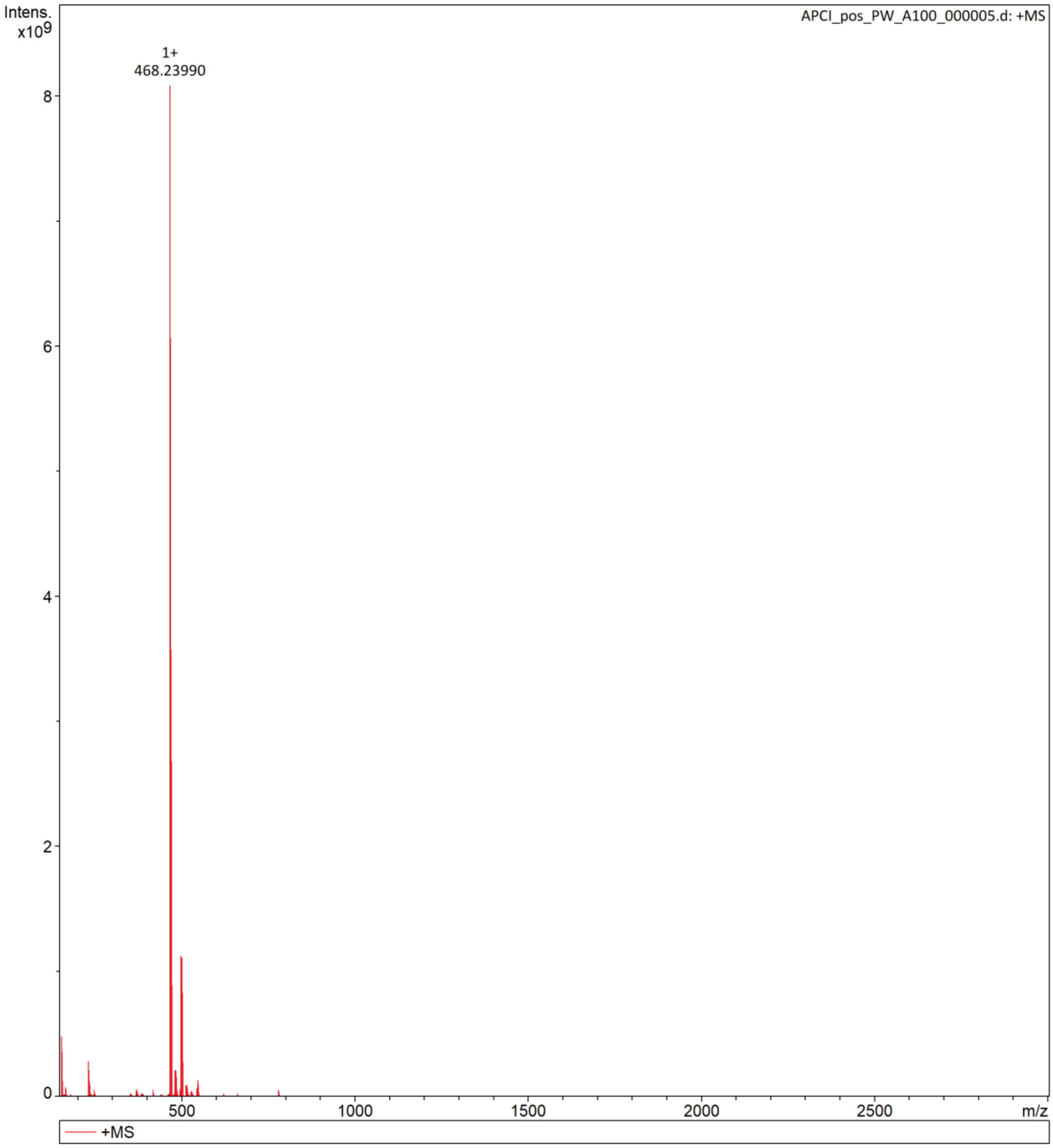

Figure S58: HR-APCI mass spectrum of 2-bromo-9,9-bis(2-ethylhexyl)-9H-fluorene.

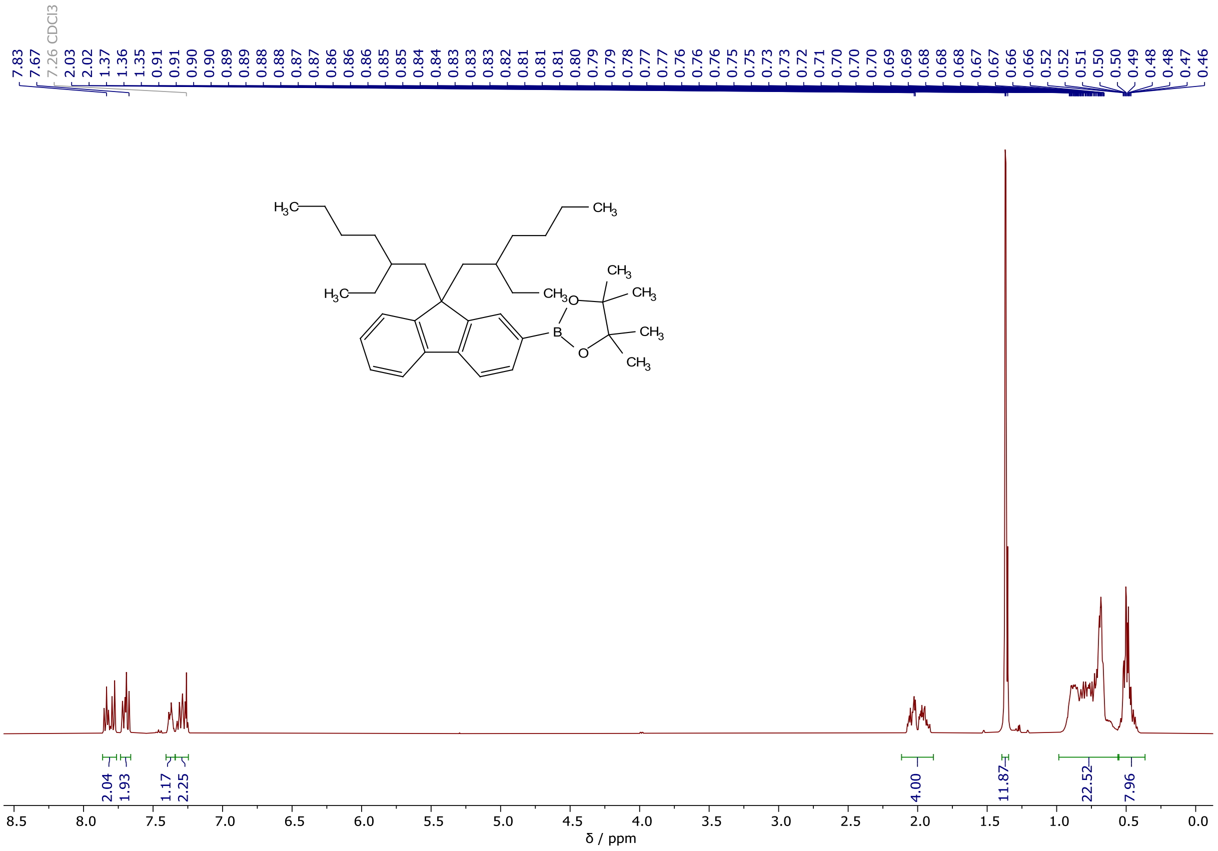

Figure S59: ^{1}H-NMR of 2-(9,9-bis(2-ethylhexyl)-9H-fluoren-2-yl)-4,4,5,5-tetramethyl-1,3,2-dioxaborolane in $CDCl_3$.

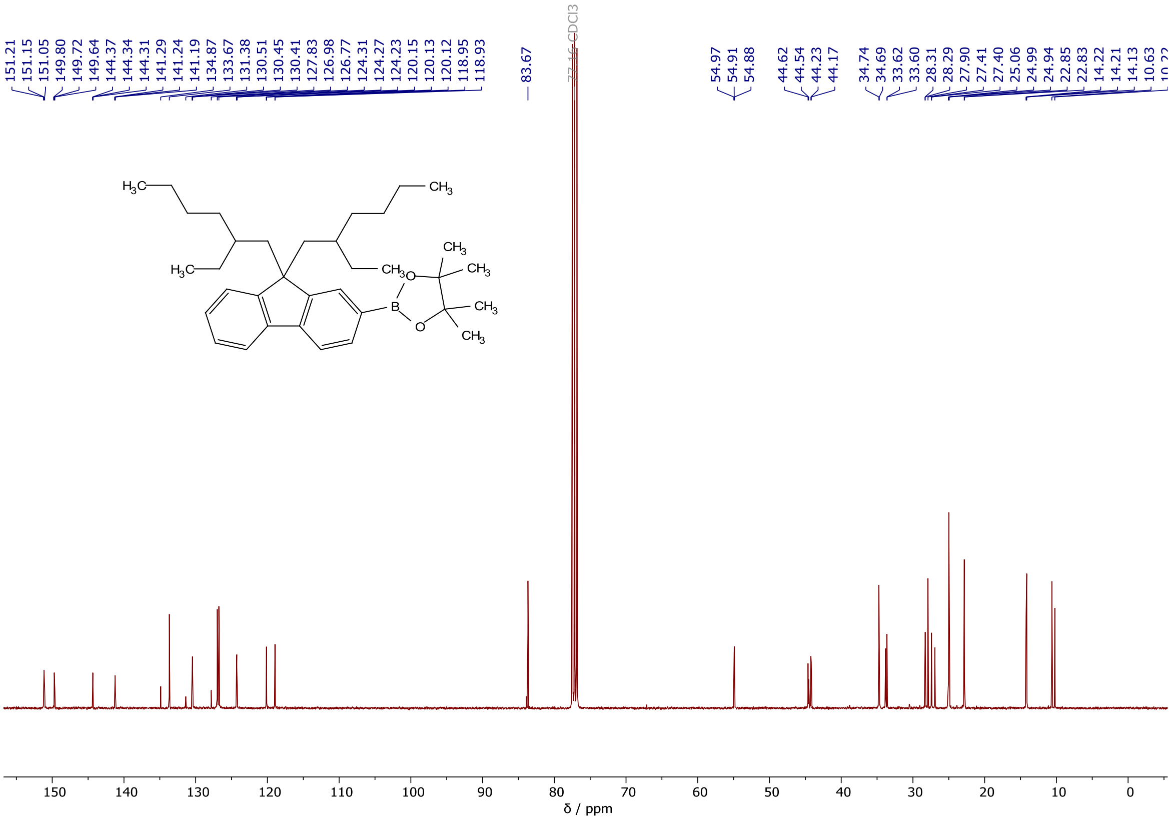

Figure S60: ^{13}C-NMR of 2-(9,9-bis(2-ethylhexyl)-9H-fluoren-2-yl)-4,4,5,5-tetramethyl-1,3,2-dioxaborolane in $CDCl_3$.

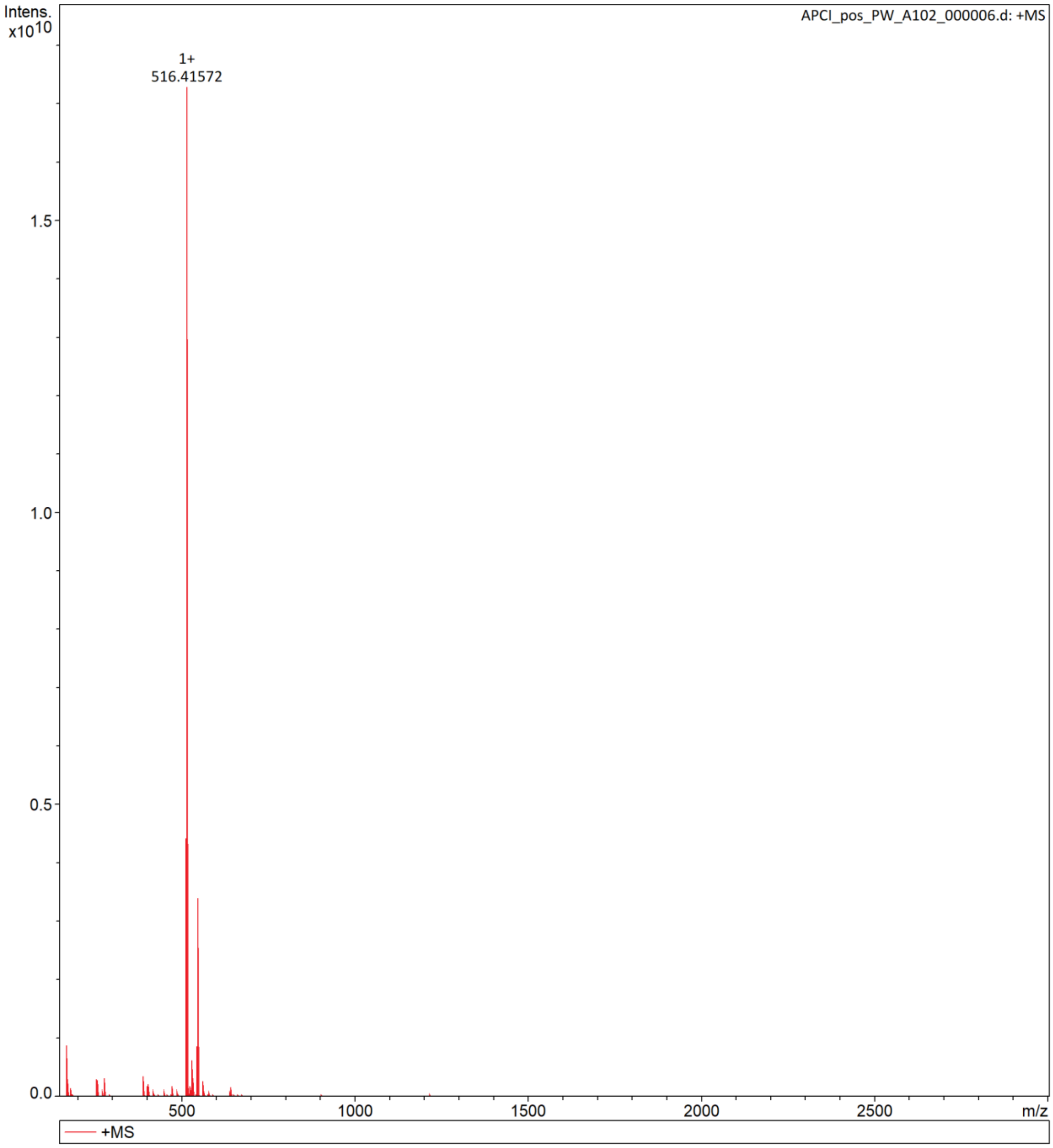

Figure S61: HR-APCI mass spectrum of 2-(9,9-bis(2-ethylhexyl)-9H-fluoren-2-yl)-4,4,5,5-tetramethyl-1,3,2-dioxaborolane**.**

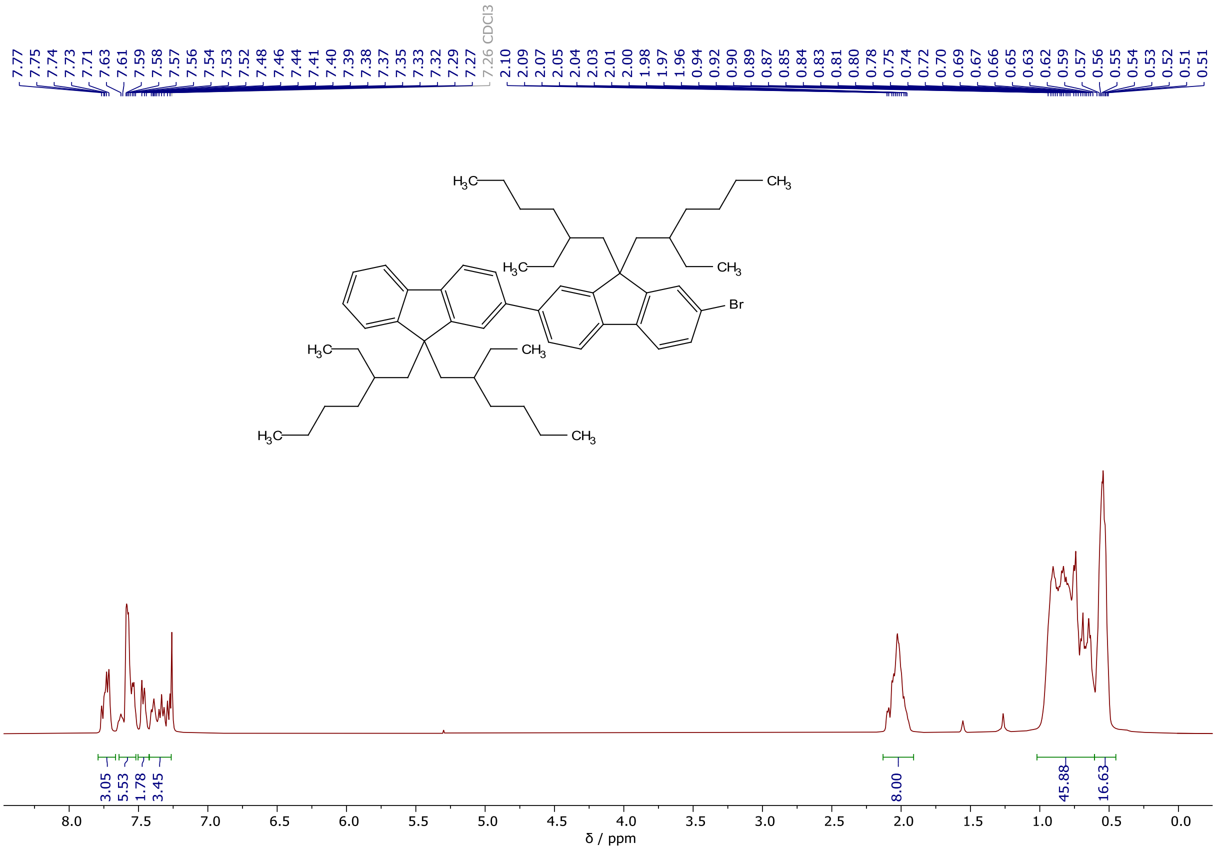

Figure S62: ^{1}H-NMR of 7-bromo-9,9,9',9'-tetrakis(2-ethylhexyl)-9H,9'H-2,2'-bifluorene in $CDCl_3$.

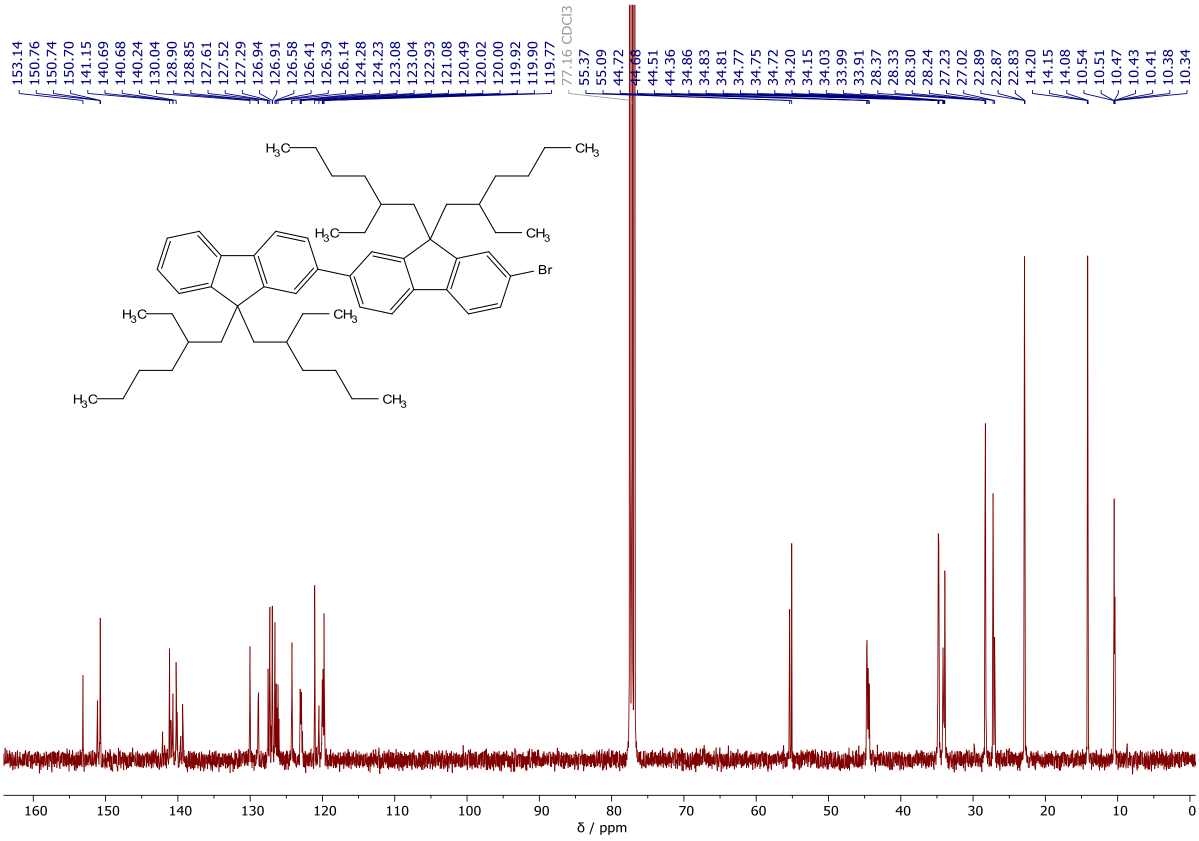

Figure S63: ^{13}C-NMR of 7-bromo-9,9,9',9'-tetrakis(2-ethylhexyl)-9H,9'H-2,2'-bifluorene in $CDCl_3$.

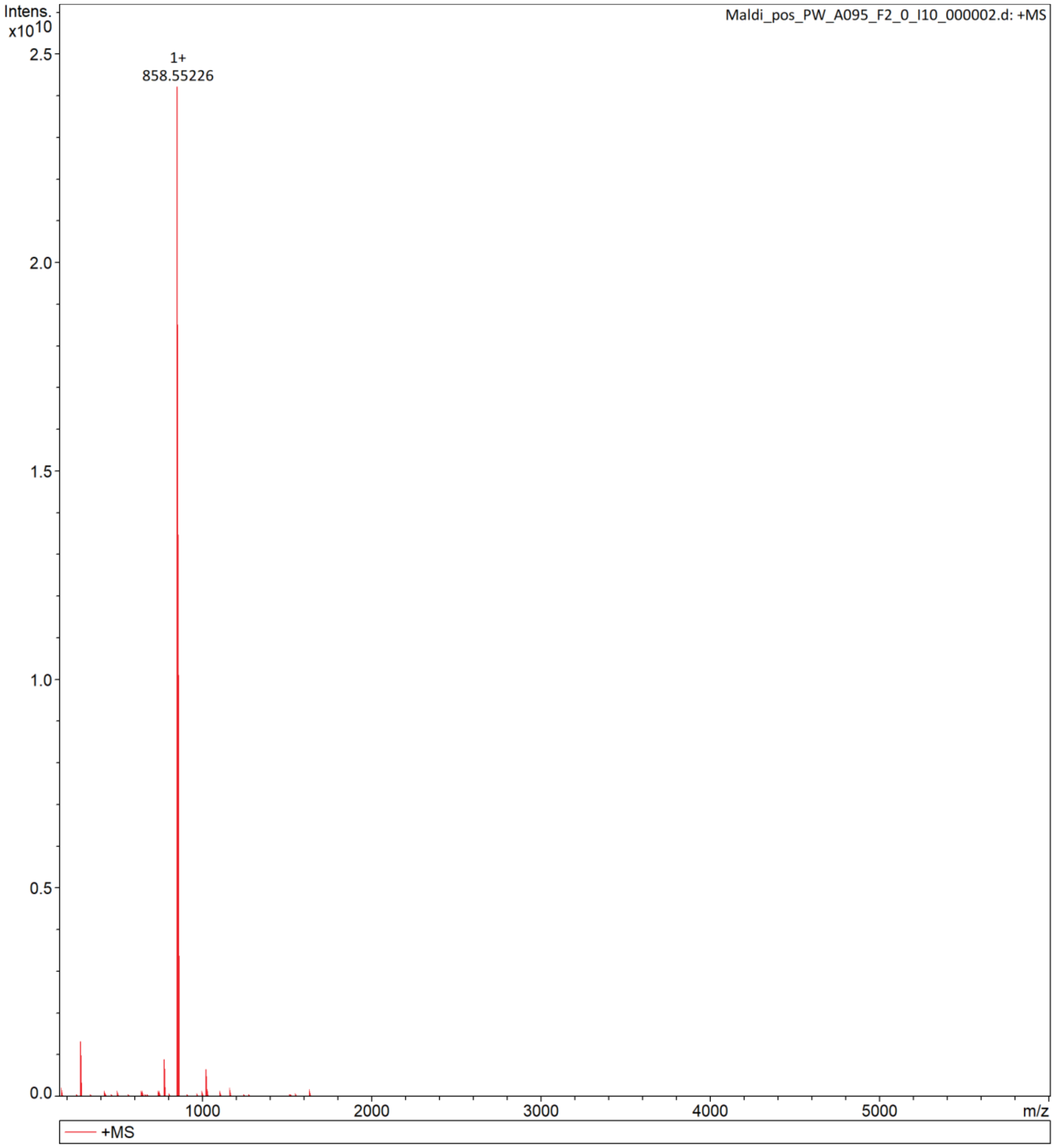

Figure S64: HR-MALDI mass spectrum of 7-bromo-9,9,9',9'-tetrakis(2-ethylhexyl)-9H,9'H-2,2'-bifluorene with DCTB as matrix.

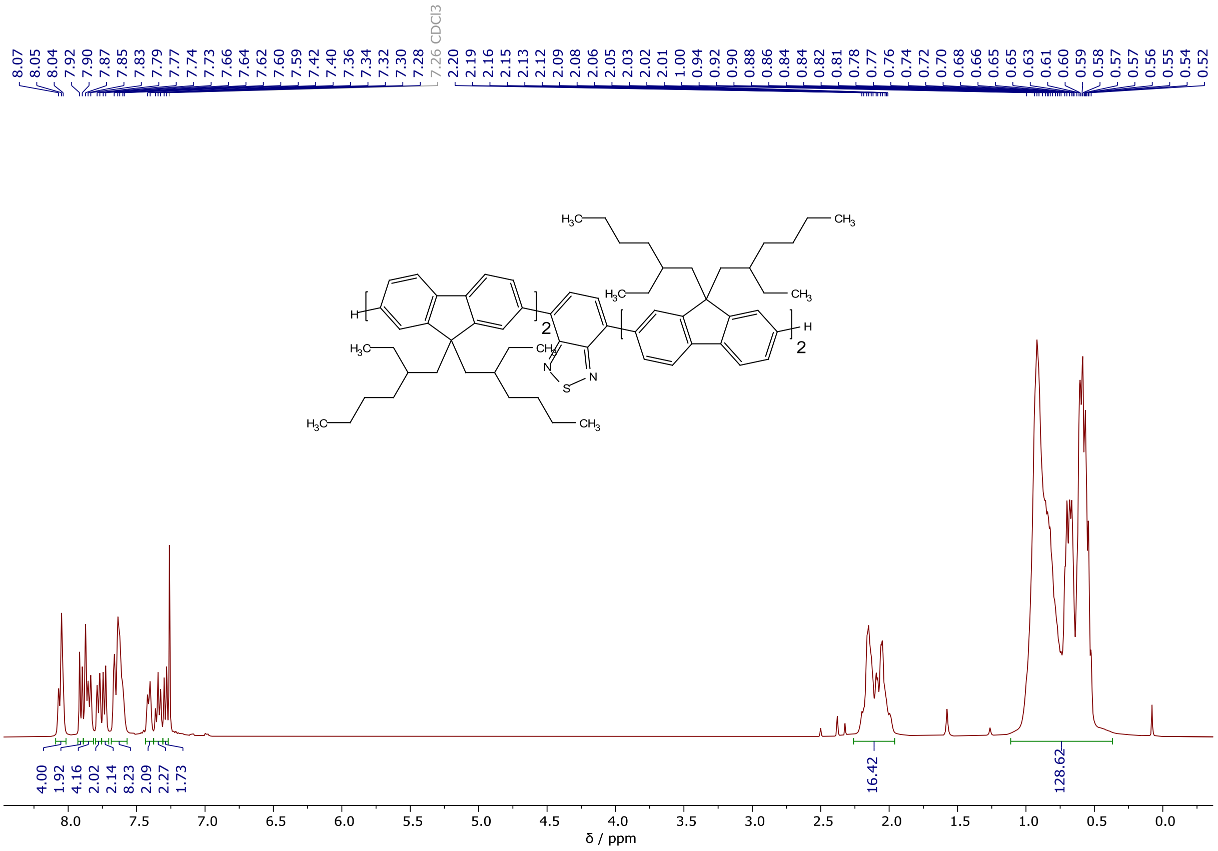

Figure S65: ^{1}H-NMR of 4,7-bis(9,9,9',9'-(2-ethylhexyl)-9H,9'H-[2,2'-bifluoren]-7-yl)benzo[c][1,2,5]thiadiazole (EH) in $CDCl_3$.

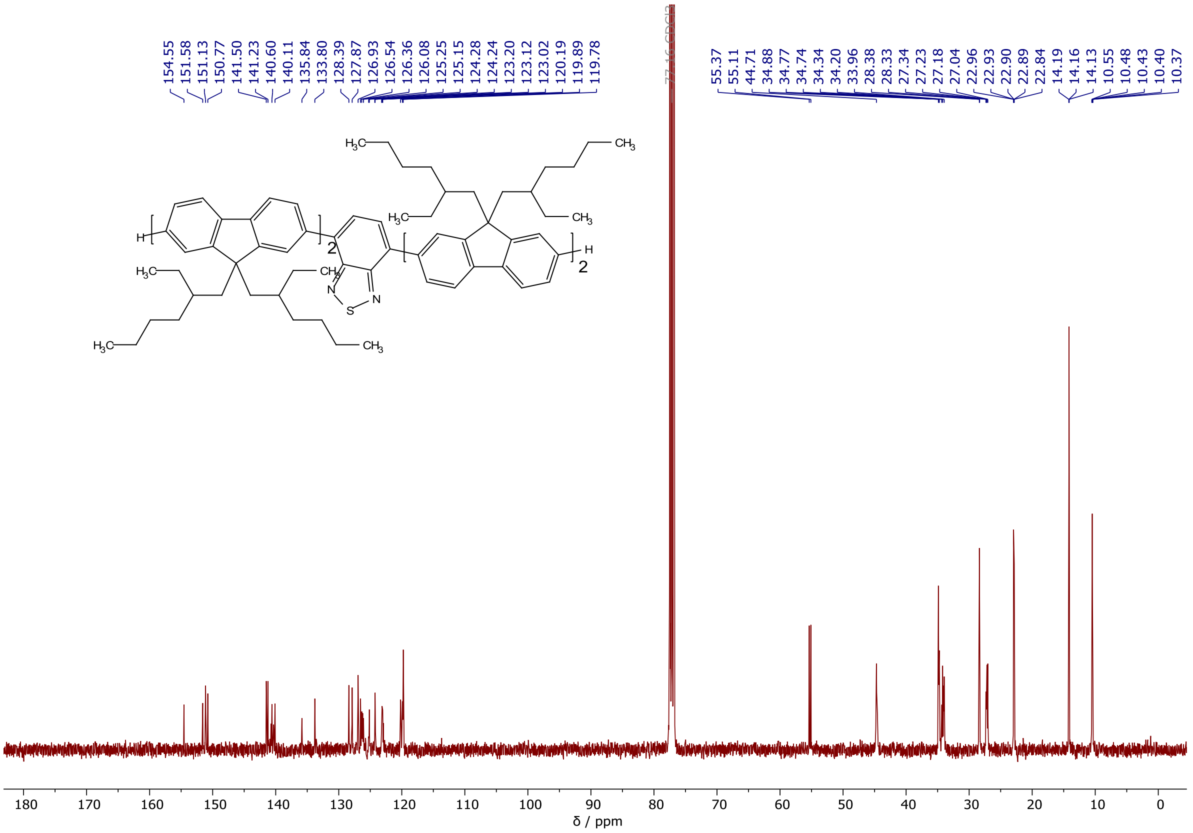

Figure S66: ^{13}C-NMR of 4,7-bis(9,9,9',9'-(2-ethylhexyl)-9H,9'H-[2,2'-bifluoren]-7-yl)benzo[c][1,2,5]thiadiazole (EH) in $CDCl_3$.

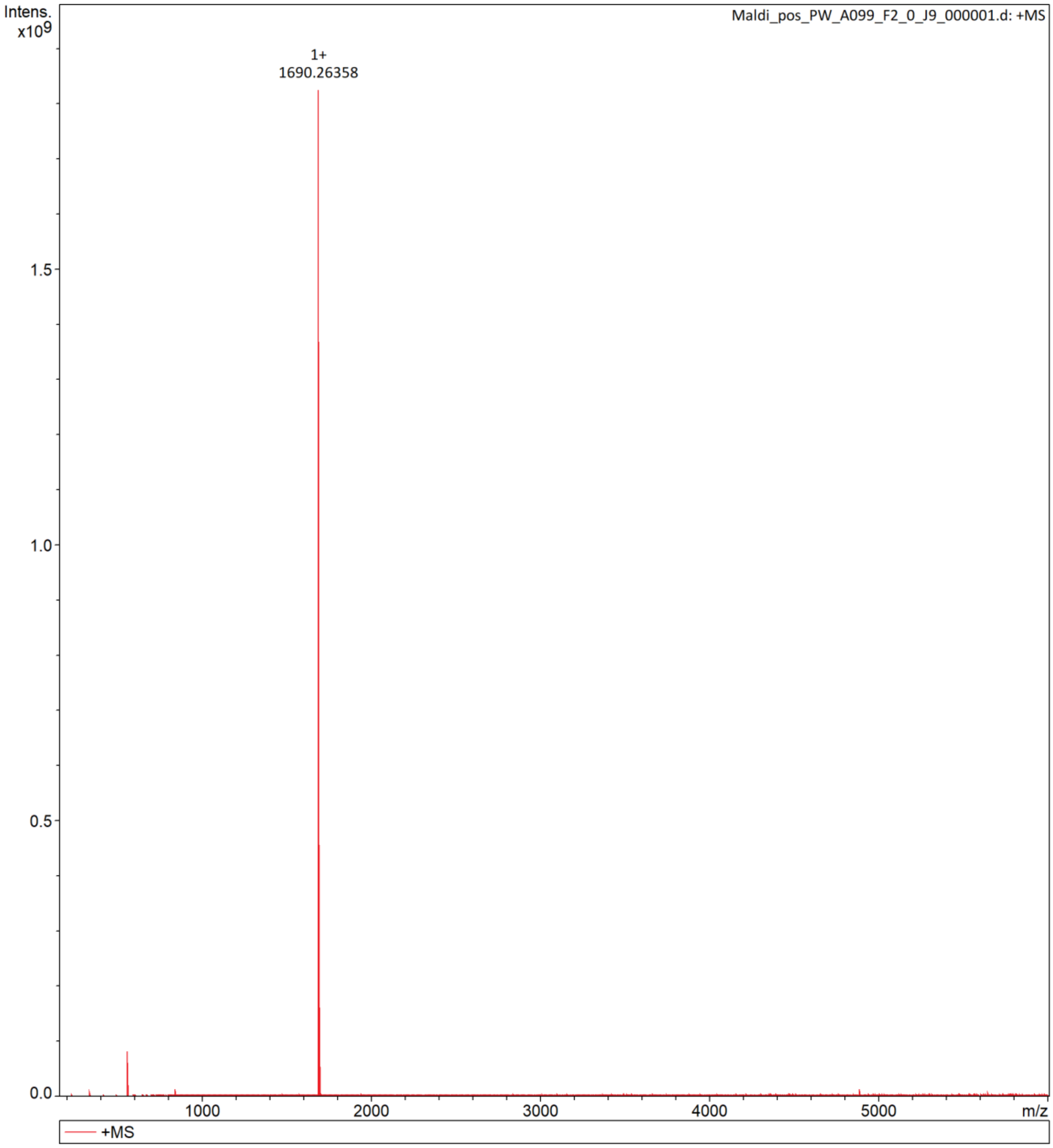

Figure S67: HR-MALDI mass spectrum of 4,7-bis(9,9,9',9'-(2-ethylhexyl)-9H,9'H-[2,2'-bifluoren]-7- yl)benzo[c][1,2,5]thiadiazole (EH) with DCTB as matrix.
